# Prediction and Analysis of Canonical EF Hand Loop and Qualitative Estimation of Ca^2+^ Binding Affinity

**DOI:** 10.1371/journal.pone.0096202

**Published:** 2014-04-23

**Authors:** Mohit Mazumder, Narendra Padhan, Alok Bhattacharya, Samudrala Gourinath

**Affiliations:** 1 School of Life Sciences, Jawaharlal Nehru University, New Delhi, India; 2 Department of Immunology, Genetics, and Pathology, Rudbeck Laboratory, Uppsala University, Uppsala, Sweden; 3 School of Computational and Integrative Sciences, Jawaharlal Nehru University, New Delhi, India; University of Hyderabad, India

## Abstract

The diversity of functions carried out by EF hand-containing calcium-binding proteins is due to various interactions made by these proteins as well as the range of affinity levels for Ca^2+^ displayed by them. However, accurate methods are not available for prediction of binding affinities. Here, amino acid patterns of canonical EF hand sequences obtained from available crystal structures were used to develop a classifier that distinguishes Ca^2+^-binding loops and non Ca^2+^-binding regions with 100% accuracy. To investigate further, we performed a proteome-wide prediction for *E. histolytica*, and classified known EF-hand proteins. We compared our results with published methods on the E. *histolytica* proteome scan, and demonstrated our method to be more specific and accurate for predicting potential canonical Ca^2+^-binding loops. Furthermore, we annotated canonical EF-hand motifs and classified them based on their Ca^2+^-binding affinities using support vector machines. Using a novel method generated from position-specific scoring metrics and then tested against three different experimentally derived EF-hand-motif datasets, predictions of Ca^2+^-binding affinities were between 87 and 90% accurate. Our results show that the tool described here is capable of predicting Ca^2+^-binding affinity constants of EF-hand proteins. The web server is freely available at http://202.41.10.46/calb/index.html.

## Introduction

Calcium signaling plays a major role in controlling most biological systems and many cellular functions, such as fertilization, motility, cell differentiation, proliferation and apoptosis, which are directly or indirectly regulated by Ca^2+^ [1]–[3]. In eukaryotes, there are elaborate mechanisms that are involved in maintaining Ca^2+^ homeostasis [4]. A defect in any of the components of the Ca^2+^ homeostasis/signaling system may have disastrous consequences including cell death. Recently many Ca^2+^-binding proteins have also been identified in bacteria and viruses, raising the possibility that the prokaryotes may also have a Ca^2+^ regulatory system, particularly in relation to host-pathogen interactions [5], [6].

Ca^2+^ is bound by a variety of proteins that are capable of binding with different affinities [7]–[9]. Such calcium binding proteins (CaBPs) can be classified into two categories, Ca^2+^ sensors and buffers. The major function of the first category of CaBPs is to sense the level of free intracellular Ca^2+^and then to activate a suitable signaling pathway [10].

In general, CaBPs contain two well-defined Ca^2+^-binding motifs: the EF hand and C2 domains [11]. The EF-hand motif is the most frequently occurring Ca^2+^-binding motif in eukaryotic systems [12]. There are more than 66 subfamilies [13] of EF–hand proteins and 3000 EF-hand related entries in the NCBI Data Bank [14]. An EF hand is composed of a typical helix-loop-helix structural unit. This group is the largest and includes well-known members, such as calmodulin, troponin C and S100B. These proteins typically undergo a calcium-dependent conformational change which opens a target binding site [13]. Proteins, such as calbindin D9k do not undergo calcium-dependent conformational changes [15]–[17].

EF-hand motifs are divided into two major structural groups: the canonical EF-hands as seen in calmodulin (CaM) and the prokaryotic CaM-like protein calerythrin, and the pseudo EF hands exclusively found in the N-termini of S100 and S100-like proteins [18]. In either structural group, a pair of EF-hand motifs or pseudo EF-hand motifs forms a structural domain and is the minimum requirement for Ca^2+^-dependent activation. In general, one of the EF-hand motifs has a higher Ca^2+^-binding affinity than the other. The canonical Ca^2+^-binding loop is characterized by a sequence of 12 amino acid residues. In an EF-hand loop the calcium ion is coordinated in a pentagonal bipyramidal configuration. The six residues involved in the binding are in positions 1, 3, 5, 7, 9 and 12; these residues are denoted by X, Y, Z, -Y, -X and -Z.

In general, affinity constants of EF-hand domains for Ca^2+^ vary from micromolar to millimolar, reflecting the diversity of functions carried out by these proteins in a range of Ca^2+^ concentrations. There is an increase in stability and change in conformation upon binding Ca^2+^. Several residues found in an EF-hand loop are highly conserved and contribute to the stabilization and proper folding of the binding site. Factors such as biological environment as well as the binding sequence have been shown to contribute to the calcium-binding affinity of these proteins [18]–[21].

A number of algorithms have been developed to computationally identify EF hand-containing CaBPs and Ca^2+^-binding regions, including statistical, machine learning and pattern search approaches [22]–[24]. Recently, Franke et al. (2010) [24]proposed a method to estimate Ca^2+^-binding affinity based on free energy calculations using crystal structures of CaBPs. However, this method has limited use due to unavailability of crystal structures in complex with calcium for large number of CaBPs. Moreover, no suitable method is available for the prediction of Ca^2+^-binding affinity from primary sequence information. There was an early attempt by Boguta et al (1988) [25] to estimate the binding affinity of calcium for troponin C (TnC) superfamily proteins based on the prediction of secondary structures. The results were convincing for some proteins which follow a typical TnC pattern [25] but not for any other protein family. Since it is not always possible to experimentally determine Ca^2+^-binding properties of EF hand-containing calcium-binding proteins, it is necessary to be able to predict this property from primary sequence. In this report we describe a method for computational prediction of Ca^2+^-binding loops and their affinities for Ca^2+^from amino acid sequences. This paper describes approaches to find a better correlation of sequence to binding affinities in order to predict the sequence to function (Ka) relationship.The results show that the tool (CAL-EF-AFi) described here is accurate and provides useful information about Ca^2+^-binding properties to experimental biologists for both characterized and uncharacterized proteins.

## Results

A few experimental methods based on biophysical techniques, such as Isothermal titration calorimetry (ITC) surface plasmon resonance (SPR) & fluorescence [26] are available for determination of Ca^2+^-binding parameters. However, these are expensive and time consuming. To the best of our knowledge, no prediction method has been developed so far that can be used to estimate Ca^2+^-binding properties of a protein from primary sequence. Therefore, a comprehensive study was carried out first to identify Ca^2+^-binding EF loops and then their Ca^2+^-binding affinities. In this study, we have constructed two support vector machines (SVM), one for prediction of loop regions and the other for estimation of binding affinity.

### Position-specific scoring matrix

After obtaining position-specific scoring matrix (PSSM) scores using equations (1) and (2) (described in Methods) for all the sequences obtained from the literature, we calculated the correlation coefficient between the experimental affinity constants (Ka) and PSSM to be 0.61 (Figure S1 in File S1). While this correlation is clearly positive, it was not possible to classify the affinity of all the sequences solely using PSSM scores. Therefore, a systematic attempt was made to first predict the presence of canonical EF-hand loops from amino acid sequence and then estimate the binding affinities qualitatively based on evolutionary information using SVMs.

### Amino acid composition distinguishes Ca^2+^-binding and non-binding regions

**Figure 1:**
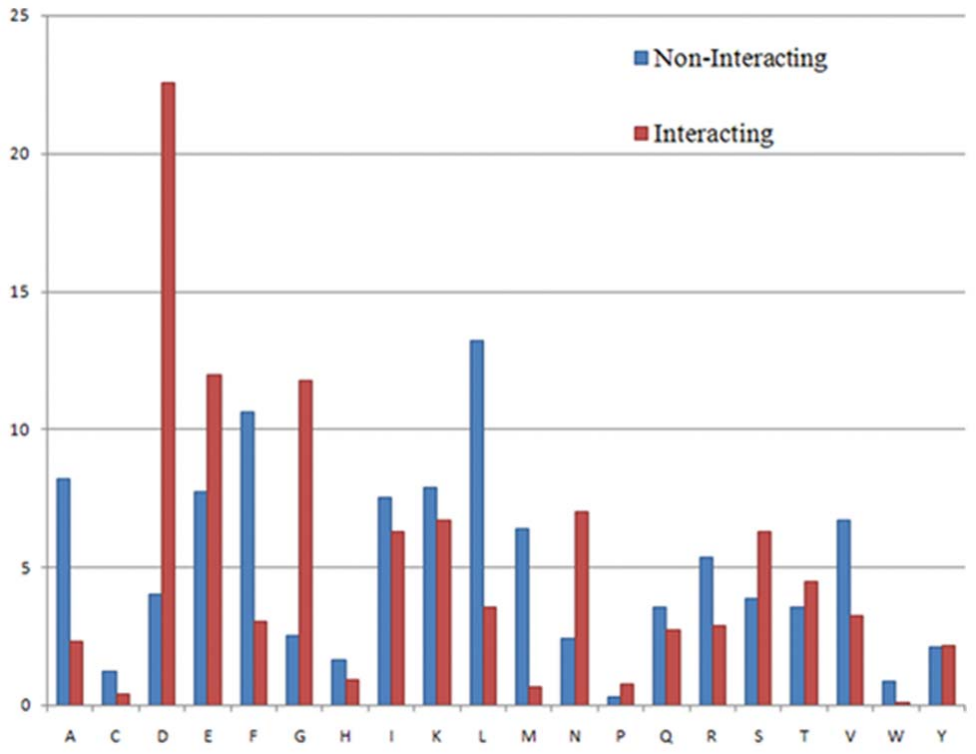
Amino acid composition of the 12-mer long Ca^2+^-binding region (“Interacting”) and the non-binding region (“Non-Interacting”) of EF-hand proteins.

A statistical analysis was carried out to determine which amino acids are found unusually frequently in EF hand-motif sequences using the entire PFAM EF-hand database. Glycine, glutamic acid, asparagine, and especially aspartate have been determined to occur more frequently in Ca^2+^-binding loop regions than in non-binding regions at a 99.9% confidence level. Alanine, phenylalanine, leucine, and especially methionine are overrepresented in non-binding regions (Figure 1). The relative frequency of amino acids at each position is listed in Table S1 in File S1. The analysis suggests that EF-hand Ca^2+^-binding loops have a specific amino acid composition, and that it is possible to identify these loops from the primary sequence.

### Experimental determination of Ca^2+^-binding properties of EhCaBPs

In order to validate the theoretical predictions, experiments were carried out to determine qualitative and quantitative aspects of the affinity of some EhCaBPs for Ca^2+^. Ca^2+^-binding properties of these proteins were tested by ^45^Ca^2+^ overlay assay on western blotted pure recombinant EhCaBP1, 3, 5, 6, and 7 proteins. All of these proteins were found to bind ^45^Ca^2+^ as observed by autoradiography (data not shown). ITC was used to determine the molar stoichiometry of the binding of the cations to these EhCaBPs, as well as the binding constants and associated thermodynamic parameters (Table 1). The sequences and binding affinities of these proteins were used in the validation dataset (D7) for validation of the classifier's efficiency on experimental data. The raw data obtained after ITC experiments are provided in the Figure S2 in File S1.

**Table 1:** Summary of macroscopic binding constants and thermodynamic parameters obtained from the ITC studies of Ca^2+^-binding isotherm of EhCaBPs at 25°C.

Ligand	Titrand	No of experimental Ca^2+^- binding sites (n)	KA (M-1)	Kd	ΔH (cal/mol)	ΔS (cal/mol)	ΔG (kcal/mol)
**Ca^2+^**	**EhCaBP1**	4	K1 = 5.25×10^3^±4.0×10^2^	130.72 µM	−1860±0	10.8	−4.84
			K2 = 1.41×10^4^±9.5×10^2^		2.3×10^5^±0	790	−4.6×10^2^
			K3 = 5.10×10^5^±2.8×10^4^		2.4×10^5^±1.82×10^3^	−780	−7.56
			K4 = 1.55×10^6^±7.3×10^4^		−7981±1.86×10^3^	1.56	−8.44
	**EhCaBP3**	2	K1 = 4.00×10^6^±5.3×10^5^	1.85 µM	−1.605×10^4^±86.6	−23.6	−9.0
			K2 = 7.28×10^4^±5.3×10^3^		−7573±10^4^	−3.16	−6.63
	**EhCaBP5**	2	K = 1.18×10^7^±1.47×10^6^	85 nM	−1.84×10^4^±61.79	−29.4	−9.64
	**EhCaBP6**	2	K1 = 1.07×10^5^±1.1×10^4^	46 µM	702±17.6	25.4	−6.86
			K2 = 4.44×10^3^±1.1×10^2^		5244±45.9	34.3	−4.97
	**EhCaBP7**	2	K1 = 1.04×10^6^±2.5×10^5^	3.12 µM	−1807±96.5	21.5	−8.2
			K2 = 9.86×10^4^±6.8×10^3^		−5413±96.5	4.69	−6.81

### SVM models predict the presence of EF loop regions

Two different models were generated using both binary pattern and amino acid composition (AAC) for loop identification. Both AAC and binary pattern were calculated, and used as input for classification of Ca^2+^-binding EF-hand loops and non-Ca^2+^-binding 12-mers in EF-hand proteins using SVM. The models were generated by using different types of kernels, such as polynomial, radial basis function (RBF), and linear. The performance of each kernel function was evaluated by five-fold cross validation. During model generation, the RBF kernel showed the best results.

The RBF kernel function using binary and AAC standalone features most accurately predicted the presence of EF-loop regions. An accuracy of 100% was achieved with D1 and D2. The remarkable performance of binary and AAC is due to the high conservation of sequence and structure among EF-hand loops that have been used in this study. Normally, the default threshold value (0) was used for the SVM classifier to discriminate between Ca^2+^-binding EF-hand loops and non-Ca^2+^-binding 12-mers in EF-hand proteins. The sites with a prediction score close to 1 are most likely to be an EF-hand calcium-binding loop region. All performance measures and the learning parameters for the RBF kernel are listed in Table 2.

**Table 2:** The Performance of SVM Models with different learning parameters on D1 and D2 dataset.

Features	C	g	SN	SP	ACC	MCC
Binary	8	0.008	100	100	100	1
AA	0.125	0.008	100	100	100	1

Using binary patterns and AA (amino acid) composition [γ **(g)** (in RBF kernel), c: parameter for trade-off between training error & margin] where SN–sensitivity, SP–specificity, ACC-accuracy, MCC–Matthews Correlation Coefficient.

### Accessibility and hydrophilic (AC&HC)-based classifier provides the best estimation of binding affinity

Various SVM models using a combination of features were developed to estimate the affinity of Ca^2+^ for the EF-hand loop. The predictions of binding constants were not as accurate as the predictions of EF-hand loops due to the limited availability of experimental data on binding constants and the high level of diversity in amino acid sequence with relation to binding affinity. In this study, we have developed a position-specific scoring matrix for EF-hand loop regions and scored (equation [1] and [2]) the sequences from the annotated data set using Perl scripts developed in-house. Based on the PSSM scores, we classified high (D3) and low (D4) binding groups for the 12-mer region to train the classifier. The binding constants, obtained from the literature (Table S2 in File S1), and data obtained from ITC studies of EhCaBPs were used as the test dataset and validation dataset (Table S3 in File S1) respectively. Since it is generally believed that different physico-chemical properties contribute to the structure and function of protein sequences, these properties should also contribute to Ca^2+^-binding affinity. Therefore, we have developed several SVM models (data not shown) to achieve better accuracy using combinations of several amino acid features, and have obtained the different physico-chemical properties using the amino acid index database (http://www.genome.jp/aaindex/).Only the best performing models are discussed here.

For the 24-dimension input vectors consisting of accessibility (AC) and charge (CC), the values of sensitivity, specificity and accuracy were 90.97, 87.10, 90.30 and 90.91, 75.00, 84.21 for training and test datasets respectively. We were also able to achieve a Matthews's correlation coefficient (MCC) of 0.78 for the training datasets (D3 & D4) and 0.67 for the test (D5) dataset.

The classifier consisting of concatenated features of accessibility (AC) and hydrophilic (HC) scores showed the best performance when tested on the training and the test datasets, achieving an MCC of 0.87 and 0.81 and an accuracy of 94.78 and 89.47 for D3–D4 and D5 datasets, respectively. The superior performance of this classifier compared to other hybrid models is also indicated by its values for sensitivity and specificity of 95.83 and 91.00 respectively for the training dataset, and 81.82 and 100.0 respectively for the test dataset.

Several other hybrid models (AC&CC, AC&HC&HYC, AC&HYC&CC and AC&HYC) were also generated with amino acid features-based classifiers; however their performances were not better than the AC&HC-based classifier. The list of figures of merit of all the classifiers used can be found in Tables 3 and 4.

**Table 3:** The Performance of SVM Models on PSSM based training dataset D3 & D4.

Features	C	g	SN	SP	ACC	MCC	AUC/ROC
AC&CC	32768	0	90.97	87.1	90.30	0.78	0.94
***AC&HC***	***8***	***0.03***	***95.83***	***91.0***	***94.78***	***0.87***	**0.97**
AC&HC&HYC	2	0.13	94.44	91.0	94.78	0.86	0.97
AC&HYC&CC	2048	0	91.67	90.32	91.42	0.82	0.96
AC&HYC	2048	0	91.67	88.7	91.04	0.8	0.95

The Performance of SVM Models on PSSM based training dataset D3 & D4 with different learning parameters on various hybrid models [γ (g) (in RBF kernel), c: parameter for trade-off between training error & margin] where SN–sensitivity, SP–specificity, ACC-accuracy, MCC–Matthews Correlation Coefficient, AUC/ROC-Area under curve/ Receiver Operating Curve.

**Table 4:** The Performance of SVM Models on test dataset D5.

Features	SN	SP	ACC	MCC
AC&CC	90.91	75.00	84.21	0.67
***AC&HC***	**81.82**	**100**	**89.47**	**0.81**
AC&HC&HYC	72.73	87.50	78.95	0.6
AC&HYC&CC	90.91	75.00	84.21	0.67
AC&HYC	90.91	75.00	84.21	0.67

The Performance of SVM Models on test dataset D5 (experimental binding affinities obtained from literature) with different learning parameters.

The quality of the performance of the AC&HC-based classifier is also indicated by receiver operating characteristic (ROC) plots, which we computed for all the models discussed in this study. ROC is commonly used to evaluate the discrimination ability of a classifier. If the area under the ROC curve is larger, it means the classifier has better discrimination ability. We were able to achieve an AUC of 0.97 with the training dataset and 0.903 with the experimental datasets (D5 & D7) using the AC&HC-based classifier (Figure S3 in File S1). A schematic representation for the data input, algorithm implementation and experimental strategy overview is shown in Figure S4 in File S1.

### Prediction of Ca^2+^binding of an independent dataset

After obtaining the best performing model, it was important to evaluate the performance of this classifier on a dataset that has not been used for training and testing. In order to check the unbiased prediction efficiency of the model, in addition to the test dataset, an independent dataset (D6) with 35 unique troponin C superfamily binding sites (Boguta et al 1988) and 15 unique sites (Table S4 in File S1) were tested using our classifier. The classifier predicted 21 high binders (true positives), 19 low binders (true negatives), and 10 high binders (false negatives) that were predicted as low binding sites. When using the diverse datasets and binding affinities obtained from different researchers working under different experimental conditions, the overall accuracy achieved was 80.0%.

### The validation dataset

The performance of AC&HC-based classier was even better when tested on the experimentally obtained binding affinities from EhCaBPs. We achieved an accuracy of 90.91 and MCC of 0.83. The performances of other classifiers for the validation dataset D7 are listed in table 5.

**Table 5:** The Performance of SVM Models on validation dataset with experimentally derived binding affinity from EhCaBPs (D7).

Features	SN	SP	ACC	MCC
AC&CC	83.33	60	72.73	0.45
***AC&HC***	**100**	**80**	**90.91**	**0.83**
AC&HC&HYC	83.33	80	81.82	0.63
AC&HYC&CC	83.33	60	72.73	0.45
AC&HYC	66.67	60	63.64	0.27

The Performance of SVM Models on validation dataset with experimentally derived binding affinity from EhCaBPs (D7)with different learning parameters on various hybrid models [γ (g) (in RBF kernel), c: parameter for trade-off between training error & margin] where SN–sensitivity, SP–specificity, ACC-accuracy, MCC–Matthews Correlation Coefficient, AUC/ROC-Area under curve/ Receiver Operating Curve.

### *E. histolytica* proteome analysis: Computational prediction of Ca^2+^-binding properties of EhCaBPs

In this section, we used ‘CAL-EF-AFi’ to scan the *E. histolytica* proteome in order to predict all Ca^2+^-binding canonical EF-hand loops in this organism. A previous computational study [27] showed that there are 27 CaBPs containing EF-hand motifs present in *E. histolytica*. Our scanning results picked all the known canonical EF hands with more than one EF-hand loop region. Apart from the sequences used in the test dataset (Ehcabp1, 3, 5–7); we also predicted the relative affinities of other EhCaBPs (8–27). In total, we predicted 36 Ca^2+^-binding sites (Table S5 in File S1) out of which 24 were predicted to be low-affinity sequences and the remaining 12 sites were predicted to have high affinity for Ca^2+^.

### Comparison with existing methods

The performance of the classifier was compared with PFAM based HMM profile search and Calpred [28] on the *E. histolytica* proteome. In light of earlier bioinformatics studies by Bhattacharya et al. and availability of *E.histolytica* strain HM-1: IMSS for wet lab experiments, we chose the *E.histolytica* proteome for comparison. Although this is not a benchmark dataset, it was important to validate our classifier's accuracy to find EF-hand containing Ca^2+^-bindingsites in large databases and proteomes. A total of 41 EF-hand protein sequences were predicted using the pattern search method whereas CAL-EF-AFi found 58 probable sequences with 153 binding loops.

Based on the results obtained by PFAM pattern search, few of the predictions with high threshold values (Table S6 in File S1) appear to be false positives. Note that the tertiary structures of all these proteins have not been determined yet, but lacks the number of amino acids required to form a typical EF hand structural motif. Similarly we scanned EhCaBPs with Calpred (using all the modules available), which identified EF-hand proteins but predicted false positives; all the residues in the full-length protein sequence were predicted as calcium binding (site). To investigate further we used sequences with known structures (D1 & D2) in Calpred and found similar false-positive predictions here as well. A thorough analysis (Table S6 in File S1) of the results from different methods for the identification of EF-hand Ca^2+^-binding sites suggests that the method proposed here to be most suitable for prediction of Ca^2+^-binding sites and relative affinity constants and is also useful for whole proteome scans.

### Availability

CAL-EF-AFi is available at http://202.41.10.46/calb/index.html and all the datasets used in the study as well as the proteome scan results are available at http://202.41.10.46/calb/dataset.html.

## Discussion

In the current era of high-throughput next generation sequencing, where a large amount of genomic data is generated each day, prediction of gene functions and detailed annotation have become key aspects of computational genomics. The focus of this study is to annotate Ca^2+^-binding EF hand motif-containing proteins and further classify these on the basis of their Ca^2+^-binding affinities.

Different Ca^2+^-binding proteins display different levels of affinities for Ca^2+^. The functions of these proteins in general depend on their affinity constants for Ca^2+^. Ca^2+^-sensor proteins such as calmodulin (CaM) display higher Ca^2+^ -binding affinities for their C-terminal domains than for their N-terminal domains [29]. Ca^2+^-buffer proteins, such as parvalbumin have high binding affinity [30] and there is little or no change in their conformation upon binding Ca^2+^. Hence it is possible to predict the probable function of the proteins from Ca^2+^-binding properties.

Many computational methods have been developed ever since identification of the first EF-hand domain as an approach for prediction of Ca^2+^-binding sites. These methods were based on similarity search, energy based calculations, Bayesian statistical methods, machine learning approaches and graph theory [22], [31]–[33], where the input is either a primary amino acid sequence or a three-dimensional structure. A comparison of CAL-EF-AFi with the existing methods for identifying Ca^2+^-binding sites is not suitable due to the dissimilarity in the prediction methods, input type and the datasets. One of the recently published machine learning approaches [28] to identify the calcium-binding region showed poor performance when compared with CAL-EF-AFi using a dataset of experimentally determined values. Some of the other methods, such as CaPS uses pattern search where EF-hand motif and Ca^2+^-binding loops are predicted on the basis of patterns generated using a Hidden Markov Model based on multiple sequence alignment of known EF-hand proteins. None of these methods, however, were able to predict the binding affinity of the identified Ca^2+^-binding motifs. We have trained the classifier using the sequences of EF hand motif binding and non-binding regions so that it could identify the Ca^2+^-binding region in the EF-hand motif.

The performance of the classifier was also tested by analysing the complete proteome of *E. histolytica*. Based on the scan results we found all of the reported Ca^2+^-binding proteins, and also identified new probable Ca^2+^-binding sites. Our tool appeared to give better results in terms of identification of CaBPs as it identified more proteins including all known CaBPs. Other methods, such as PFAM-based HMM profile search and Calpred showed a significant number of false predictions. Our results, using all of the sequences in the test (D5) affinity estimation data set, suggest that the PSSM scores and experimental binding affinities are broadly correlated. In our study, we have classified proteins on the basis of relative binding affinity for Ca^2+^ in a semi-quantitative manner. There are a number of reasons that a precise quantitative analysis is still intractable. For one, a 12-mer motif alone does not determine the affinity since there may be contributions from other parts of the protein. Also, there is a cooperative involvement of more than one EF-hand loop in the binding of Ca^2+^. This may be particularly important as a pair of EF hands occur together [14]. Two EF-hand motifs in a pair (with very few exceptions) are related by an approximate two-fold rotational axis, forming a hydrophobic cavity opening which is likely to influence the binding affinity. Since these properties are difficult to factor in a model, our efforts are limited to classification of high and low binders rather than predicting precise binding affinities.

Our initial datasets contained 19 binding sites with experimental binding affinity data. In order to circumvent the problems associated with limited data, we have generated training datasets based on the evolutionary information (PSSM) scores. A similar approach, where artificial datasets have been used in SVM, has been successful in greatly improving predictions [34], [35]. In these studies, researchers have mainly generated negative datasets artificially for SVM classification. Our test data set with 19 sequences, independent dataset with 50 sequences and the validation data set with 11 sequences representing experimentally determined affinity data have shown extremely good results.

The results from the test and validation datasets, which includes relative affinities of several EF-hand proteins, suggest that our proposed model based on the PSSM method for estimation of binding affinity can help researchers to predict site-specific binding affinity. Experimental determination of such binding affinity is a limiting factor in Ca^2+^-binding proteins because of the expense involved and time required carrying out the experiments. As mentioned above, the successful performance of the model with regards to prediction and estimation is attributed to the accurate training of the classifier on a small number of training examples and the use of PSSM generated datasets.

CAL-EF-AFi can therefore be used to accurately and precisely scan proteomes of organisms for potential Ca^2+^-binding sites of EF-hand proteins and estimate their probable relative binding affinities. Given the success of our classifier on the *E. histolytica* proteome scan, we expect its wider use in analysing proteomes of other organisms.

In conclusion, we have developed a unique method, CAL-EF-AFi for identification and estimation of Ca^2+^-binding sites and relative affinity. The program requires only the protein sequence for the prediction without prior knowledge of structural or biochemical information. The results predicted by the theoretical model were validated by experimental studies. Variation from the EF-hand consensus sequence can be used to predict qualitative Ca^2+^-binding features. However, this may not be sufficient to understand the overall characteristics of CaBPs. The EF-hand motifs assemble to form a lobe (one partner affects the binding affinity of the other) and the Mg^2+^ affinities are not considered in this work due to limitation of experimental data available to date. Future plans include developing an even better algorithm with more information available from the literature. We hope that an increase in the availability of experimental data will help generate a more robust model.

## Material and Methods

### Expression, Purification and Preparation of Metal-free Protein Solutions

Five different EhCaBPs (EhCaBP1, 3, 5, 6, and 7) were overexpressed and purified as described earlier [36], [37]. In order to obtain accurate measurements of Ca^2+^-binding energetics, it was essential to have the protein in its apo-form with no contamination of Ca^2+^ in the buffers. Hence, all of the buffers used for isothermal titration calorimetry (ITC) were decalcified using Chelex 100 resin (Bio-Rad). Decalcified ITC buffer (100 mMNaCl and 50 mM Tris-Cl, pH 7.0) was prepared by treatment with Chelex 100 resin (Bio-Rad). Each protein solution was treated with 5 mM EGTA and 2 mM EDTA to remove Ca^2+^ and Mg^2+^. The EDTA/EGTA bound to metal ions were removed from protein solution using Amicon ultra centrifugal filter devices (Millipore), through extensive buffer exchange (decalcified). Before the ITC experiment, the sample cell and injection syringe of the ITC machine (Microcal Inc.) were extensively cleaned using the decalcified buffer.

### Isothermal Titration Calorimetry (ITC)

All ITC experiments were performed on a MicroCal VP-ITC microcalorimeter at 25 C. Samples were decalcified, centrifuged, and degassed prior to titration. A typical titration consisted of injecting 2-µl aliquots of 10–20 mM CaCl_2_ solution (diluted from 1 M standard CaCl_2_ solution supplied by Sigma-Aldrich Chemicals) into 100–200 µM protein solution after every 3 min to ensure that the titration peak returned to the baseline prior to the next injection. A total of 70 injections were carried out. Aliquots of concentrated ligand solution were injected into the buffer solution (without the protein) in a separate ITC run, to subtract the heat of dilution. Two sets of titrations were carried out for each protein: (i) apo-EhCaBP in 50 mM Tris-Cl, pH 7.0 and 100 mMNaCl and (ii) holo-EhCaBP in 50 mM Tris-Cl, pH 7.0 and 100 mMNaCl. The ITC data were analysed using the software ORIGIN (supplied with Omega Microcalorimeter). The amount of heat released per addition of the titrant was fitted to the best least squares model as given by Wiseman et al. (1989). For each titration, the stoichiometry (n), association constant (Ka), and enthalpy change (ΔH) were obtained directly from the ITC data, and the changes in Gibbs free energy (ΔG), and entropy (ΔS), as well as the overall binding affinity or dissociation constant (Kd) were calculated according to Equations a, b, and c.
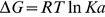
(a)




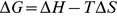
(b)




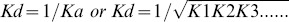
(c)


### Dataset for EF loop predictions

To predict the presence of EF-hand loops and estimate their affinities for Ca^2+^, the calcium-binding amino acid sequence pattern at PROSITE [38](http://prosite.expasy.org/PDOC00018) was used to retrieve sequences of the EF-hand family. In total 1379 different sequences were obtained. To further validate the reviewed sequences we used structures of proteins co-crystallized with calcium from the Protein Data Bank [39] (PDB, http://www.rcsb.org/pdb/). In total 1261 chains with EF-hand motifs were found. Once these sequences were downloaded, CD-HIT [40] was used to remove redundant sequences having more than 60% similarity. The PDB IDs are included in the supplementary data in File S1 (Tables S7–S10 in File S1) along with the sequences retrieved. We chose a relatively high because the aim of the study was to identify the binding loop, which is a highly conserved 12-residue sequence. With less than a 60% threshold, the numbers of sequences available for classification were not sufficient. The sequence classifications were also carried out using thresholds of 90%, 70%, 60%, 50% of CD-HIT data is also shown in Table S11 in File S1. Finally a dataset of 100 12-mer calcium-binding loop sequences for the positive training dataset (D1) was generated. Similarly a negative training dataset was built with 141 (D2) 12-mer sequences extracted from non-binding regions of EF-hand proteins.

### Dataset for binding affinity predictions

For the estimation of binding affinity, a novel method was developed on the basis of PSSM score pattern in which calcium-binding loops were classified into two groups. Based on the correlation obtained between the PSSM scores and experimental binding affinity (Figure S1 in File S1) a positive dataset with high PSSM scores (D3) (>5) consisting of 144 12-mer sequences and a negative dataset (D4) with low PSSM scores (<5) containing 124 sequences were generated using the sequences obtained from PROSITE [38].

To test the proposed model based on PSSM scores we used 19 EF loop sequences for which binding affinities were known from the literature (Table S2 in File S1) as Test dataset (D5). To evaluate the performance of this classifier on a dataset that has not been used for training and testing, an independent dataset (D6) of binding affinity observations was obtained from Boguta et al (1988) [25]and recently published literature. After removing redundant EF-loop sequences, 50 unique sequences were obtained from recently published data and the Ka values listed in Boguta et al (1988) [25].Furthermore, to check the performance and reliability of the classifier, we chose to perform ITC experiments on available EhCaBPs, to test our predictions on the datasets obtained from literature. We were able to obtain Ka values of EhCaBP1, 3, 5, 6, and 7; in total we listed affinities for 11 sites used here as a validation set (D7). The details of ITC experiments and results are also provided in supplementary datasets in File S1 as D5, D6 and D7 with their experimental binding affinities classified on the basis of a thorough review of published papers that reported the binding constants. The classification details with supportive binding constants are listed under “Author's Note” in Tables S2–S4 in File S1.

### Statistical Analysis

The expected (Exp) frequencies of amino acid residues were calculated from the average residue usage from the 1379 different sequences obtained from PROSITE [38]. The expected frequency for an amino acid residue of type A at position i will be Exp  =  *(NA/N) M*, where *NA* = total number of amino acid residues of type A in the analysed set of sequences, excluding position *i, N* = total number of all amino acid residues in the analysed set of sequences, excluding position *i,* and *M* = total number of sequences, i.e., the sum of *i*th positions in the analysed set of sequences. The expected frequencies for residues were calculated similarly. For each amino acid residue at a given position, the deviation of the observed (Obs) values from the Exp values was estimated by the χ^2^ criterion according to the formula (Obs – Exp)^ 2^/Exp. For each residue or codon, the χ^2^ value was estimated separately with one degree of freedom. The sums of all 20 (61) χ^2^ values for each residue (codon) at the given position gave the total deviation for the given position with 19 (60) degrees of freedom. To evaluate the range of differences between the C-terminal regions and the neighbouring fragments, a pairwise comparison between them was performed. For this purpose, each position in the sequence was treated as a set containing 20 groups of data and the difference between them was calculated by the χ^2^ criterion using the following formula:
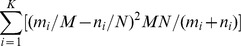
where m_i_ and n_i_are frequencies of amino acid residues in the two positions of the sequence under comparison, M and N are total numbers of amino acid residues in the compared positions, and K is equal to 20 because each position may be occupied by any of 20 different amino acids. At a significance level <0.001, Obs was considered to be different from Exp if the χ2 exceeded 10.8, 43.8 and 99.6 for one, 19 and 60 degrees of freedom, respectively.

### Generation of a position-specific scoring matrix

In this study, a simple position-specific scoring matrix (PSSM) was generated from the amino acid composition (AAC) of the calcium-binding loops in canonical EF hands. The standard amino acid frequencies, which show how often each residue was found in each site in the binding loop, was taken from Marsden et al., 1990 [41]. In this matrix, every column can be interpreted as a discrete probability distribution of the amino acid residues at that position and the values in the matrix can be inferred as probabilities of a given amino acid occurring at a given position. Therefore, for a sequence of length m, the product of the relative frequencies from the matrix corresponding to each amino acid in each position of the sequence is the probability of discovering such a sequence in the EF-hand loop. We generated two different scoring matrices, one with simple relative frequency of amino acids and the other with log likelihood frequency for the position-specific scoring matrix [42]–[44]. The log ratio matrix was generated using equation 1 and 2.
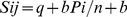
(1)




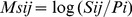
(2)Where Sij is the probability of amino acid i at position j in matrix S, q is the observed counts of amino acid type i at position j, Pi is the probability of amino acid type i, b is the pseudo count which is considered here as square root of the total number of training sequences and n is the number of training sequences. In equation (2) Msij represents the foreground model (representing true homology) and Pi is the background model (chance that a match occurs at random). The background probability or the chance of amino acid match occurrence at random was calculated using the BLOSUM62 substitution matrix [45].

### Support Vector Machine training for classification

SVM is a machine learning tool that is being extensively used for classification and optimization of complex problems. It is particularly attractive to biological sequence analysis due to its ability to handle noise, large datasets, large input spaces and high variability [46], [47]. In this study all of the SVM models have been developed using libSVM [48]. Parameter selection was carried out using grid search so that the classifier can accurately predict unknown test data from the model. In the radial basis function (RBF) kernel, there are two parameters, C and g, but it is not known *a priori* what values of these two parameters are best for a given problem [48]. To obtain the best parameters, a grid search was carried out using cross validation. A Perl script was written in-house to check combinations of features in an iterative manner using CUDA based libSVM [49]. A descriptive flowchart of the feature selection algorithm is provided in Figure S4 in File S1.

### Five-fold cross-validation

A standard five-fold cross-validation technique was used to evaluate the performance of models, where the data set was randomly divided into five sets. The classifier was trained on four sets and the performance was assessed on the remaining fifth set. The process was repeated five times so that each set could be used once for testing. Finally, the average of the five sets was calculated as the measure of the performance of the classifier.

### SVM model using binary and amino acid composition features

In this method, a Perl program was written to generate a window with 12 amino acids for negative and positive patterns. These sequence patterns were converted into binary patterns, where a pattern of length L was represented by a vector of dimension L×21 and each amino acid in that pattern was represented by a 21-feature vector (e.g. Asp by 1,0,0,0,0,0,0,0,0,0,0,0,0,0,0,0,0,0,0,0,X) containing 20 amino acids and a dummy X. Each sequence of twelve amino acids was represented by 252 input vectors during model generation. The binary profile has been used in a number of existing methods [50], [51]. The second feature used was AAC with an input vector of 20X12 dimensions. AAC is the fractional occurrence of each amino acid in the protein sequence.
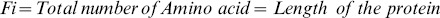
Where i can be any of the amino acids.

### Feature extraction and model generation for binding affinity estimation

It has been observed in different studies [52], [53] that SVM performs well when combinations of two or more features are used as input vectors. Hence, hybrid models have been developed using one or more combinations of features. After testing combination of features using CUDA-based libSVM [49] the best performing features were used for developing various SVM models. Feature selection was carried out by scanning amino acid indices and by performing 5-fold cross validation using the in-house CUDA script. The four best performing amino acid properties used further for analysis were net charge [54](CC), hydrophobicity [55] (HYC), hydrophilicity [56] (HC) and accessibility [57] (AC) which were thus used for further analysis. Only the better performing models(AC&CC, AC&HC, AC&HYC, AC&HC&HYC, and AC&HYC&CC), which use combinations of the four best performing amino acid properties, are discussed in this study.

### Classifier performance metrics

The performance of our method was computed and tested using the following figures of merit. As mentioned above, the performance has been evaluated by five-fold cross validation as follows:

1. Sensitivity (or recall) is the coverage of positives i.e. the percent of correctly predicted Ca^2+^-binding 12-mers and correct estimation of their affinity.
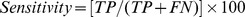

2. Specificity is the coverage of negatives, that is, the percent of correctly predicted Ca^2+^ non-binding 12-mers and correct estimation of their affinity.
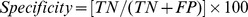

3. Accuracy is the percentage of correctly predicted positives and negatives.
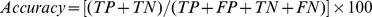

4. MCC – Matthews's correlation coefficient is the statistical parameter to assess the quality of the prediction and account for unbalancing in data [58]. An MCC equal to 1 is regarded as a perfect prediction, whereas that equal to 0 indicates a completely random prediction.

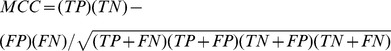
 [TP  =  true positive; FN  =  false negative; TN  =  true negative; FP  =  false positive]

1. AUC (Area under the ROC Curve) – Receiver Operating Curve (ROC) and AUC were computed using SPSS software. It generates ROC curves and calculates AUC by ranking the decision values.

## Supporting Information

File S1 **File S1 includes the following: Figure S1.** a) Plot of affinity vs. PSSM for the test data set (D5). The calculated correlation coefficient obtained was 0.61 using [41] amino acid frequencies. **Figure S2.** The isothermal titration calorimetric analysis of Ca^2+^-binding to apo-EhCaBPs.ITC experiments were carried out as described under “Materials and Methods”. Plot of heat absorbed/released (In kcal mol^−1^) per injection of CaCl_2_ as a function of molar ratio of Ca^2+^: protein at 25°C is shown. For all titrations, the top panels represent the raw data (power: time) and the bottom panels represent integrated binding isotherms. The solid line represents the best nonlinear fit to the experimental data. Binding isotherm for A: EhCaBP3; B: EhCaBP4; C: EhCaBP5; D: EhCaBP6 and E: EhCaBP7. Thermodynamic parameters obtained are summarized in Table 1. **Figure S3.** ROC plots of AC&CC, AC&HC, AC&HC&HYC, AC&HYC&CC and AC&HYC for the datasets D5–D7 set. Receiver operating characteristic (ROC) plot used for depicting relative trade-offs between true positive and false positives. The corresponding AUC value of each model is shown in brackets. **Figure S4.** Schematic representation of the procedure for model development and feature selection for EF-hand loop region prediction and estimation of binding affinity and its web implementation. The procedure is explained in detail in the “Methods” section. A). A group of sequences with known EF-hand structural motifs were downloaded and further classified into two groups after removing redundant sequences using CD-HIT. The sequences were further converted into binary and amino acid composition (AAC) profiles for SVM input. Models were generated using LIBSVM and were tested on all the datasets (D3–D6) and further validated by scanning the E. *histolytica* proteome. B). Non-redundant sequences of EF-hand loops from known structures were classified into two groups on the basis of scores obtained from position-specific scoring metrics. The sequences were then converted into binary, AAC and different amino acid indices patterns. We have generated both standalone and combinations of features (2, 3, 4, 5) using a Perl script written in-house. The input vectors were trained using LIBSVM and cudized LIBSVM and selected on the basis of their performance on experimental datasets using 5-fold cross validation accuracy threshold >70 %. The best performing models selected from screening were further validated using three different experimentally derived datasets on EF hand motifs. The final step involved web implementation of the best (AC&HC) model. **Table S1.** The χ^2^ value for each amino acid residue is estimated with one degree of freedom and significance level P = 0.001. The Σχ^2^ values are estimated with 19 degrees of freedom and significance level P<0.001. The expected (Exp) and observed (Obs) values and the corresponding χ^2^ values for amino acid residues and the Σχ^2^ values for those positions that do not reach 10.8 and 43.8 (for one and 19 degrees of freedom, respectively) are given more significance. **Table S2.** Test Dataset: Summary of EF hand loops obtained from the literature and their macroscopic binding constant along with CAL-EF-AFi predictions (D5). The classification details with supportive binding constants are listed under “Author's Note”. (Red-colored affinities are the false negative affinity predictions, and turquoise-colored sequences are the false negative EF loop predictions). **Table S3.** Independent dataset (D6) summary of EF hand loops obtained from Boguta, et al., 1988 [59]. The table contains average binding constants of Ca^2+^ for troponin C superfamily (TnC) proteins from experimental data reported by various laboratories. The classification details with supportive binding constants are listed under “Author's Note”. (Red-colored affinities are the false positive predictions). **Table S4.** Validation dataset summary of EF-hand loops obtained from ITC studies of CaBPs from *E. histolytica* and their macroscopic binding constant according to CAL-EF-AFi's predictions (D7). The classification details with supportive binding constants are listed under “Author's Note” (Red-colored affinities are the false positive predictions). **Table S5.** Predictions of putative EF hand-containing calcium-binding protein and their calcium-binding affinities from the *E. histolytica* proteome. **Table S6.** The performance and comparison of CAL-EF-AFi with PFAM and Calpred on the E. *histolytica* proteome. Listed are the sequences predicted by CAL-EF-AFi followed by PFAM-based HMM model prediction and CalPred's predictions. (Legends for CAL-EF-AFi's prediction: number of Ca^2+^-binding loop sequence prediction, residue number followed by sequence and SVM scores; Legends for PFAM predictions: red-colored region is the loop region predicted, followed by the E-value for the sequence; Legends for CalPred predictions: X: Non-Binding region C: Calcium Binding region). **Table S7.** Calcium-binding EF-hand protein sequences in FASTA format at 60% sequence redundancy with EF-hand loop region residues labeled in lower case letters. **(D1). Table S8.** The list of 12-mer sequences from non-binding regions of calcium-binding EF-hand proteins greater than 60% sequence redundancy. **Table S9.** The training data used for estimation of binding affinity were taken from the RCSB based on PSSM scores obtained from the EF-hand loop region. The positive dataset (**D3**) consisted of one hundred forty four 12-mer sequences and there were 124 sequences in the negative dataset (**D4**). **Table S10.** The redundant set of PDB ids of EF hand-containing calcium-binding proteins. The sequences taken from the RCSB were further processed using CD-HIT and the list if the sequences with different threshold are listed in Table S11. **Table S11.** The sequence-wise classification of data obtained from PROSITE and RCSB- The data was further processed by using CD-HIT at 90%, 70%, 60%, 50% sequence redundancy cutofffor classification of EF-hand loop Ca^2+^-binding and non-binding region.
(DOC)
