# Supplementary material for: Prediction and Analysis of Canonical EF Hand Loop and Qualitative Estimation of Ca2+ Binding Affinity
Source: PLoS One. 2014 Apr 23;9(4):e96202. doi: 10.1371/journal.pone.0096202 (PMC3997525; doi:10.1371/journal.pone.0096202)
Supplement: File S1 — includes the following: Figure S1. a) Plot of affinity vs. PSSM for the test data set (D5). The calculated correlation coefficient obtained was 0.61 using [41] amino acid frequencies. Figure S2. The isothermal titration calorimetric analysis of Ca2+-binding to apo-EhCaBPs.ITC experiments were carried out as described under “Materials and Methods”. Plot of heat absorbed/released (In kcal mol−1) per injection of CaCl2 as a function of molar ratio of Ca2+: protein at 25°C is shown. For all titrations, the top panels represent the raw data (power: time) and the bottom panels represent integrated binding isotherms. The solid line represents the best nonlinear fit to the experimental data. Binding isotherm for A: EhCaBP3; B: EhCaBP4; C: EhCaBP5; D: EhCaBP6 and E: EhCaBP7. Thermodynamic parameters obtained are summarized in Table 1. Figure S3. ROC plots of AC&CC, AC&HC, AC&HC&HYC, AC&HYC&CC and AC&HYC for the datasets D5–D7 set. Receiver operating characteristic (ROC) plot used for depicting relative trade-offs between true positive and false positives. The corresponding AUC value of each model is shown in brackets. Figure S4. Schematic representation of the procedure for model development and feature selection for EF-hand loop region prediction and estimation of binding affinity and its web implementation. The procedure is explained in detail in the “Methods” section. A). A group of sequences with known EF-hand structural motifs were downloaded and further classified into two groups after removing redundant sequences using CD-HIT. The sequences were further converted into binary and amino acid composition (AAC) profiles for SVM input. Models were generated using LIBSVM and were tested on all the datasets (D3–D6) and further validated by scanning the E. histolytica proteome. B). Non-redundant sequences of EF-hand loops from known structures were classified into two groups on the basis of scores obtained from position-specific scoring metrics. The sequences were the [file pone.0096202.s001.doc]

SUPPLEMENTARY INFORMATION

**Supplementary Figure S1** a) Plot of affinity vs. PSSM for the test data set (D5).The calculated correlation obtained was 0.61 using amino acid frequencies.

**Supplementary Figure S2** Isothermal titration calorimetric analysis of Ca^2+^-binding to apo-EhCaBPs**.** ITC experiments were carried out as described under “Materials and methods”. Plot of kcal mol^-1^ of heat absorbed/released per injection of CaCl_2_ as a function of molar ratio of Ca^2+^: protein at 25°C is shown. For all titrations, the top panels represent the raw data (power:time) and the bottom panels represent integrated binding isotherms. The solid line represents the best nonlinear fit to the experimental data. Binding isotherm for A: EhCaBP3; B: EhCaBP4; C: EhCaBP5; D: EhCaBP6 and E: EhCaBP7. Thermodynamic parameters obtained are summarized in the Table 1.

**Supplementary Figure S3** ROC plot of the best performing SVM classifiers. ROC plot of AC&CC, AC&HC, AC&HC&HYC, AC&HYC&CC and AC&HYC for the datasets D5-D7 set. Receiver operating characteristic (ROC) plot used for depicting relative trade-offs between true positive and false positives. The corresponding AUC value of each model is shown in brackets.

**Supplementary Figure S4** Schematic representation of the procedure for model development and feature selection for EF-Hand loop region prediction and estimation of binding affinity and its web implementation. The procedure is explained in details in the “Methods” section. A). A group of sequences with known EF-Hand structural motif were downloaded and further classified into two groups after removing the redundancy using CD-HIT. The sequences were further converted into binary and amino acid composition (AAC) profile for SVM input. Models were generated using LIBSVM and were tested on all the datasets (D3-D6) and further validated by comparing and scanning E. *histolytica* proteome. B). Non-redundant sequences of EF-hand loops from known structures were classified into two groups on the basis of scores obtained from position specific scoring metrics. The sequences were then converted into binary, AAC and different amino acid indices patterns. We have generated both standalone and combination of features (2, 3, 4, 5) using in house Perl script. The input vectors were trained using LIBSVM and cudized LIBSVM and selected on the basis of their performance on experimental dataset using 5- fold cross validation accuracy threshold > 70 %. The best performing models selected from screening were further validated using three different experimentally derived datasets on EF hand motifs. The final step involved web implementation of the best (AC&HC) model.


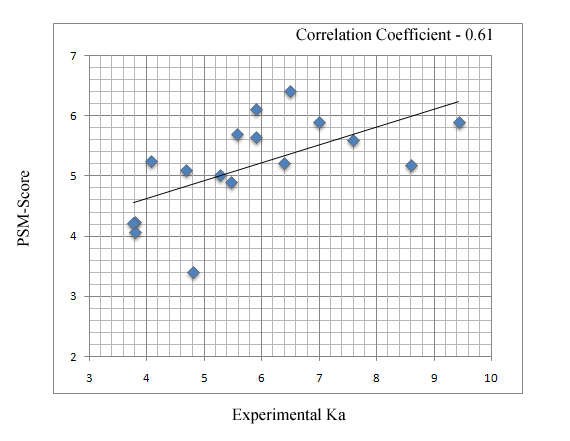


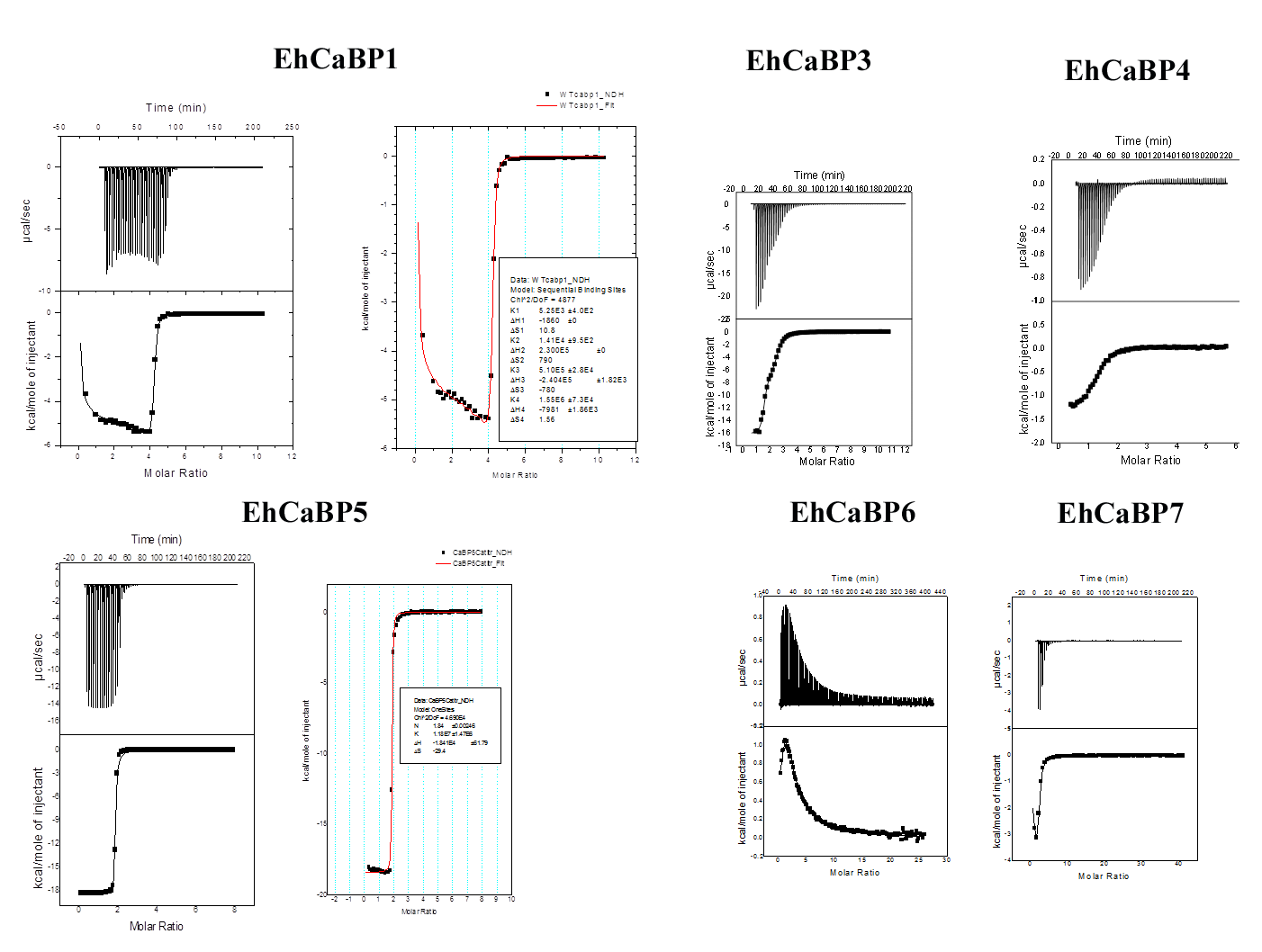


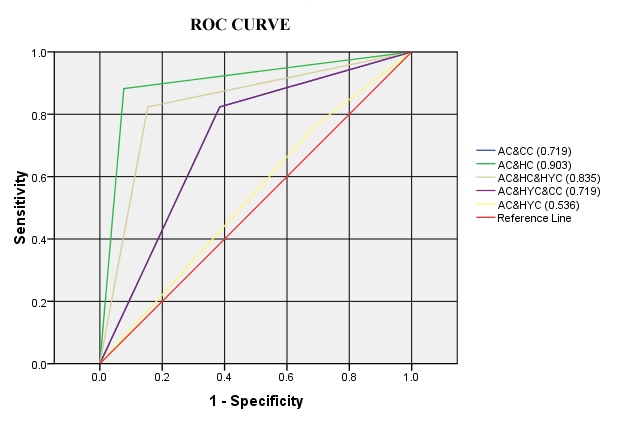


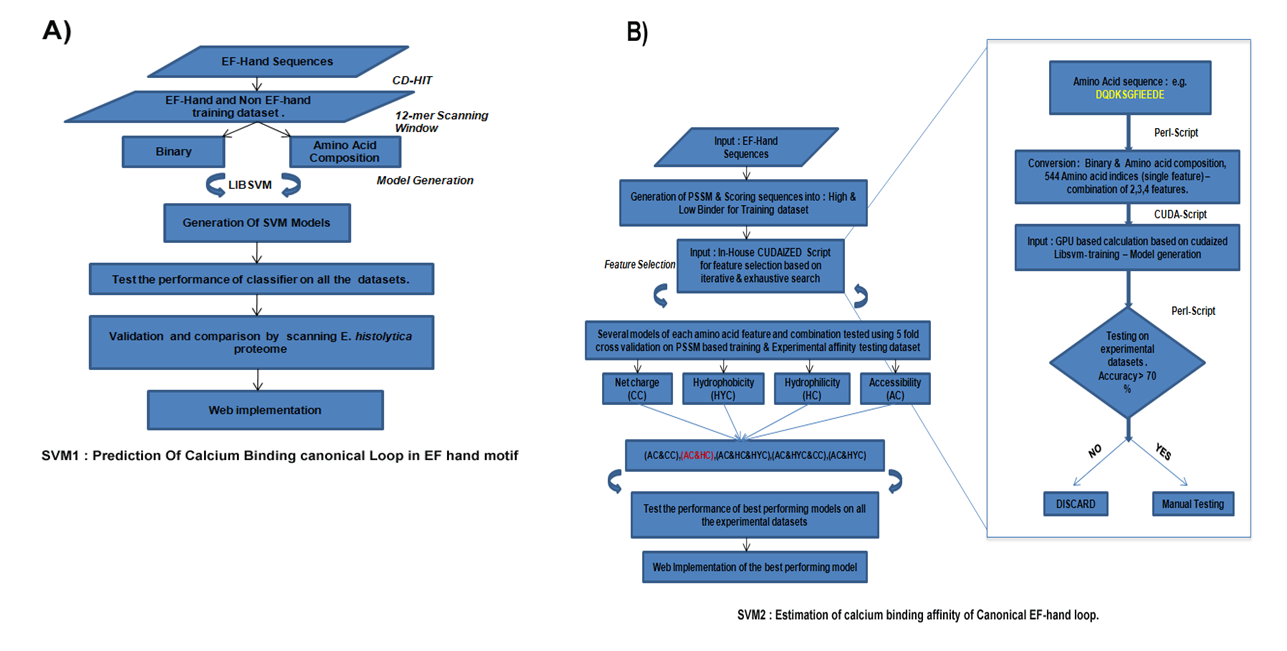


Supplementary Table S1. The χ2 value for each amino acid residue is estimated with one degree of freedom and signiﬁcance level P = 0.001. The Σχ2 values are estimated with 19 degrees of freedom and signiﬁcance level P < 0.001. The expected (Exp) and observed (Obs) values and the corresponding χ2 values for amino acid residues and the Σχ2 values for those positions that do not reach 10.8 and 43.8 (for one and 19 degrees of freedom, respectively) are given more significant.

| **POSITION** | AA RESIDUE | Number of Occ/Obs | Expected | chi-sq |
| --- | --- | --- | --- | --- |
| **1** | E | 279 | 76.73 | 533.22 |
|  | H | 25 | 11.34 | 16.47 |
|  | Q | 49 | 27.62 | 16.56 |
|  | R | 50 | 37.67 | 4.03 |
| **2** | A | 70 | 50.08 | 7.92 |
|  | C | 25 | 7.40 | 41.90 |
|  | F | 83 | 65.25 | 4.83 |
|  | I | 166 | 58.24 | 199.40 |
|  | L | 302 | 73.41 | 711.78 |
|  | V | 132 | 43.51 | 179.93 |
|  | W | 21 | 4.29 | 65.18 |
| **3** | D | 118 | 102.31 | 2.41 |
|  | E | 101 | 82.88 | 3.96 |
|  | H | 17 | 11.61 | 2.50 |
|  | K | 197 | 60.59 | 307.12 |
|  | Q | 71 | 26.86 | 72.57 |
|  | R | 118 | 35.32 | 193.51 |
| **4** | A | 72 | 50.01 | 9.67 |
|  | E | 231 | 78.39 | 297.11 |
|  | K | 130 | 62.90 | 71.57 |
|  | N | 50 | 37.78 | 3.95 |
|  | Q | 62 | 27.17 | 44.67 |
|  | R | 75 | 36.81 | 39.62 |
| **5** | A | 210 | 45.24 | 599.99 |
|  | I | 176 | 57.89 | 240.95 |
|  | L | 107 | 80.15 | 8.99 |
|  | M | 148 | 31.38 | 433.34 |
|  | V | 100 | 44.62 | 68.73 |
| **6** | F | 525 | 49.98 | 4514.94 |
|  | I | 146 | 58.93 | 128.65 |
|  | M | 78 | 33.80 | 57.79 |
|  | W | 31 | 3.94 | 185.84 |
| **7** | A | 85 | 49.56 | 25.34 |
|  | K | 158 | 61.94 | 148.99 |
|  | N | 65 | 37.26 | 20.66 |
|  | Q | 73 | 26.79 | 79.73 |
|  | R | 141 | 34.53 | 328.32 |
|  | S | 76 | 41.75 | 28.09 |
| **8** | E | 175 | 80.32 | 111.59 |
|  | H | 35 | 10.99 | 52.45 |
|  | K | 79 | 64.67 | 3.18 |
|  | L | 123 | 79.60 | 23.67 |
|  | M | 62 | 34.36 | 22.24 |
|  | V | 74 | 45.52 | 17.82 |
| **9** | A | 102 | 48.98 | 57.41 |
|  | F | 247 | 59.59 | 589.46 |
|  | I | 104 | 60.38 | 31.51 |
|  | L | 120 | 79.70 | 20.38 |
|  | M | 54 | 34.63 | 10.83 |
|  | V | 113 | 44.17 | 107.25 |
|  | Y | 92 | 16.18 | 355.44 |
| **10** | D | 896 | 75.42 | 8928.60 |
| **11** | A | 70 | 50.08 | 7.92 |
|  | K | 208 | 60.21 | 362.78 |
|  | P | 23 | 3.97 | 91.07 |
|  | Q | 50 | 27.58 | 18.22 |
|  | R | 76 | 36.77 | 41.84 |
|  | T | 123 | 31.69 | 263.04 |
|  | V | 77 | 45.42 | 21.97 |
| **12** | D | 536 | 87.86 | 2285.84 |
|  | N | 262 | 30.45 | 1760.79 |
| **13** | G | 358 | 45.31 | 2157.81 |
|  | H | 37 | 10.92 | 62.27 |
|  | K | 158 | 61.94 | 148.99 |
|  | N | 126 | 35.15 | 234.81 |
|  | Q | 41 | 27.89 | 6.16 |
|  | R | 67 | 37.09 | 24.13 |
| **14** | D | 463 | 90.38 | 1536.20 |
|  | N | 146 | 34.46 | 361.05 |
|  | S | 194 | 37.67 | 648.68 |
| **15** | G | 805 | 29.86 | 20120.37 |
| **16** | F | 105 | 64.49 | 25.44 |
|  | K | 160 | 61.87 | 155.66 |
|  | Q | 67 | 26.99 | 59.29 |
|  | R | 65 | 37.15 | 20.87 |
|  | T | 91 | 32.80 | 103.27 |
|  | Y | 104 | 15.76 | 494.03 |
| **17** | I | 559 | 44.66 | 5924.32 |
|  | L | 163 | 78.22 | 91.91 |
|  | V | 152 | 42.82 | 278.34 |
| **18** | D | 240 | 98.09 | 205.31 |
|  | N | 96 | 36.19 | 98.86 |
|  | S | 231 | 36.39 | 1040.58 |
|  | T | 163 | 30.31 | 580.84 |
| **19** | F | 196 | 61.35 | 295.54 |
|  | P | 35 | 3.56 | 277.66 |
|  | R | 50 | 37.67 | 4.03 |
|  | V | 65 | 45.83 | 8.02 |
|  | Y | 105 | 15.73 | 506.79 |
| **20** | D | 191 | 99.78 | 83.39 |
|  | E | 241 | 78.04 | 340.26 |
|  | N | 71 | 37.05 | 31.11 |
|  | P | 17 | 4.18 | 39.29 |
|  | Q | 68 | 26.96 | 62.48 |
|  | S | 60 | 42.30 | 7.40 |
| **21** | D | 84 | 103.48 | 3.67 |
|  | E | 755 | 60.28 | 8006.97 |
| **22** | F | 415 | 53.78 | 2426.20 |
|  | L | 315 | 72.96 | 802.92 |
|  | W | 16 | 4.46 | 29.88 |
|  | Y | 29 | 18.35 | 6.18 |
| **23** | C | 41 | 6.84 | 170.48 |
|  | K | 140 | 62.56 | 95.87 |
|  | L | 108 | 80.12 | 9.70 |
|  | Q | 57 | 27.34 | 32.18 |
|  | R | 107 | 35.70 | 142.37 |
|  | V | 105 | 44.45 | 82.49 |
| **24** | A | 112 | 48.63 | 82.58 |
|  | E | 107 | 82.67 | 7.16 |
|  | H | 39 | 10.85 | 73.00 |
|  | Q | 58 | 27.30 | 34.51 |
|  | R | 66 | 37.12 | 22.47 |
|  | S | 65 | 42.13 | 12.41 |
|  | T | 68 | 33.59 | 35.23 |
| **25** | A | 162 | 46.90 | 282.46 |
|  | G | 71 | 55.23 | 4.50 |
|  | I | 106 | 60.31 | 34.61 |
|  | L | 147 | 78.77 | 59.10 |
|  | M | 125 | 32.18 | 267.76 |
|  | V | 100 | 44.62 | 68.73 |
| **26** | C | 29 | 7.26 | 65.13 |
|  | I | 75 | 61.38 | 3.02 |
|  | L | 274 | 74.38 | 535.75 |
|  | M | 214 | 29.10 | 1174.75 |
|  | V | 89 | 45.00 | 43.02 |
| **27** | A | 84 | 49.60 | 23.86 |
|  | H | 24 | 11.37 | 14.03 |
|  | K | 170 | 61.52 | 191.27 |
|  | Q | 55 | 27.41 | 27.78 |
|  | R | 110 | 35.60 | 155.49 |
|  | S | 88 | 41.34 | 52.68 |
|  | T | 72 | 33.46 | 44.40 |
| **28** | A | 93 | 49.29 | 38.77 |
|  | H | 27 | 11.27 | 21.97 |
|  | K | 128 | 62.97 | 67.15 |
|  | N | 56 | 37.57 | 9.04 |
|  | R | 74 | 36.84 | 37.47 |
|  | S | 112 | 40.51 | 126.18 |
|  | T | 56 | 34.01 | 14.22 |
| **29** | I | 77 | 61.31 | 4.01 |
|  | L | 215 | 76.42 | 251.31 |
|  | M | 56 | 34.56 | 13.30 |
|  | T | 59 | 33.91 | 18.57 |
|  | V | 64 | 45.86 | 7.17 |

Supplementary Table S2. Test Dataset: Summary of EF hand loops obtained from literature and their macroscopic binding constant along with CAL-EF-AFi predictions (D5). The classification details with supportive binding constants are listed under “Author’s Note”. (RED colored affinities is the false negatives affinity predictions, Turquoise colored sequences are the false negatives EF loop predictions)

| **Protein** | **Organism** | **EF-Loop Prediction** | **K_A_ (M^-1^) from the whole protein** | **Authors Notes** | **Predicted**  **Affinity** | **References** |
| --- | --- | --- | --- | --- | --- | --- |
| **Parvalbumin** | *Cyprinus carpio* | DQDKSGFIEEDE  DSDGDGKIGVDE | K1= 2.7x10^9^  K2=2.7x10^9^ | The two metal sites of parvalbumin for Ca^2+^ with equilibrium constants of K_Ca_ = 2.7 X 10 ^9^M^-1^ | High Affinity  High Affinity |  |
| **Calmodulin** | *Bos taurus* | DKDGDGTITTKE  DADGNGTIDFPE  DKDGNGYISAAE  DIDGDGQVNYEE | K1 = 1 x 10^7^;  K2 = 3.98 x 10^7^;  K3 = 3.16 x 10^6^;  K4 = 2.5 x 10^6^ | Calmodulin contains four relatively high affinity Ca^2+^sites | High Affinity  High Affinity  High Affinity  High Affinity |  |
| **Caltractin** | *Chalmydomonas reinhardtii* | DTDGSGTIDAKE  DKDGSGTIDFEE  DDDNSGTITIKD  DRNDDNEIDEDE | K1 = 8.30 x 10^5^;  K2 = 8.30 x 10^5^,  K3 = 6.25 x 10^3^;  K4 = 6.25 x 10^3^ | Ca^2+^ binding mea-surements demo-nstrated the binding of four Ca^2+^ ions to caltractin with two higher affinity and two lower affinity sites. | High Affinity  High Affinity  Low Affinity  Low Affinity | , |
| **calmodulin-like protein** | *Homo sapiens* | DKDGDGCITTRE  DRDGNGTVDFPE  DKDGNGFVSAAE  DTDGDGQVNYEE | K1=3.80 x 10^5^,  K2=1.90 x 10^5^,  K3=4.90 x 10^4^,  K4=1.20 x 10^4^ | Four Ca^2+^-binding sites. Binding of the first two *Ca^2^*^+^occurs with somewhat higher affinity than that of the last two Ca^2+^. | High Affinity  Low Affinity  Low Affinity  Low Affinity |  |
| **Calbindin D9k** | *Bos taurus* | DKNGDGEVSFEE | K1 = 1.6 x 10^8^,  K2 = 4 x 10^8^ | Ca^2+^ ion binding to calbindin D9k wild type and with different set of mutants.(High Affinity ) | Low Affinity |  |
| **Calgranulin C** | *Sus scrofa* | DANQDEQVSFKE | K1=6.50 x 10^4^ | The protein binds one Ca^2+^/monomer with a binding constant of about 2 x 10^4^,a low affinity site | Low Affinity |  |
| **GF14-loop1** | *Arabidopsis* | ELDTLGEESYKD | K1=5.50 x 10^4^ | Low binding affinity exhibited by GF14 ω. | Low Affinity |  |
| **Calhepatin** | Lepidosiren paradoxa | DKDKSGTLSVDE  DTNKDGQVSWQE | K1=2.90 x 10^5^  K2=6.00 x 10^3^ | The affinity const-ants determined agree with the fact that S100 protein affinity for Ca^2+^ is low, the affinity of the C-terminal EF-hand being greater than that of the N-terminal EF-hand. | Low Affinity  Low Affinity |  |

| **Organism** | **Protein** | **EF-Loop Prediction** | **K_A_ (M^-1^)** | **Predicted Affinity** | **Authors Notes** | **Reference** |
| --- | --- | --- | --- | --- | --- | --- |
| *E. histolytica* | **ECaBP1 I**  **Ecabp1 II**  **Ecabp1 III**  **Ecabp1 IV** | DVNGDGAVSYEE  DADGNGEIDQNE  DVDGDGKLTKEE  DANGDGYITLEE | 5.25E+03  1.41E+04  5.10E+05  1.55E+06 | Low Affinity  High Affinity  High Affinity  High Affinity | EhCaBP1 has one high-affinity site for Ca^2+^ and Mg^2+^, one high affinity Ca^2+^-specific site and two low-affinity Ca^2+^-specific sites | *(*Table 5) |
|  | **ECaBP3 I**  **ECABP3 III** | DKDNDNKLTAEE  DKEKNGYISASE | 7.28E+04  4.00E+06 | Low Affinity  High Affinity | One binding site has affinity in the micromolar range, While the other has affinity in the sub-micromolar range. |  |
|  | **ECaBP5** | DGDGDGYLTLNE | 1.18E+07 | High Affinity | High Binding site. |  |
|  | **ECaBP6 I**  **ECaBP6 II** | DRDYDGKIDVKQ  DQDKDGKIKASD | 1.07E+05  4.44E+03 | High Affinity  Low Affinity | One high affinity Ca^2+^ binding site and one low binding site. |  |
|  | **Ecabp7 I**  **Ecabp7 III** | DKDKSGYLSPDE  DEDGDGKISFQE | 9.86E+04  1.04E+06 | Low Affinity  High Affinity | One high affinity Ca^2+^ binding site and one low binding site. |  |

Supplementary Table S3. Validation dataset Summary of EF hand loops obtained from ITC studies of CaBPs from *E. histolytica* and their macroscopic binding constant with CAL-EF-AFi’s predictions (D7). The classification details with supportive binding constants are listed under “Author’s Note” (RED colored affinities are the false positives predictions)

Supplementary Table S4. Independent dataset (D6) Summary of EF hand loops obtained from Boguta, et al., 1988 . The table contains average binding constants of Ca^2+^ for troponin C superfamily (TnC) proteins from experimental data reported by various laboratories. The classification details with supportive binding constants are listed under “Author’s Note” (RED colored affinities are the false positives predictions)

| **Protein** | **Sequences/Canonical EF loops Predicted** | **Authors Note** | **Predicted**  **Affinity** | **References** |
| --- | --- | --- | --- | --- |
| **Bovine chains αα** | I).DEDGDGEVDFQE | Contains low affinity calcium binding sites. The lower affinity calcium-binding sites titrated at a lower pH. | Low Affinity |  |
| **Bovine chains αβ** | I).DSDGDGECDFQE |  | Low Affinity |  |
| **Human chains**  **β β** | I).DNDGDGECDFQE | Six Ca^2+^-binding sites which assumed to represent three for each β-monomer. Each β subunit was shown to bind one calcium ion with rather high affinity and two other calcium ions with lower affinity. | Low Affinity |  |
| **Rat Chain β β** | I).DEDGDGECDFQE | Rat brain S100b protein is characterized by two high-affinity Ca^2+^ binding sites with a KD of 2 X 10(-5) M and four lower affinity sites with KD about 10(-4) M. | Low Affinity |  |
| **Frog pI 4-50**  **(FPV4- 50)** | II).DQDKSGFIEEDE  III).DSDGDGKIGVDE | Muscular parvalbumins from hake proteins have two high affinity sites | High Affinity  High Affinity |  |
| **Frog pl 4.88** | I).DQDQSGFIEKEE  II).DKDGDGKIGVDE | Parvalbumins exhibit two independent and equivalent high affinities Ca^2+^-Mg^2+^ sites. | High Affinity  High Affinity |  |
| **Pike pl 5.00** | I).DADASGFIEEEE | The intrinsic phenyl-alanine and tyrosine fluorescence of pike parvalbumins monitors the binding of Ca^2+^ ions to both their high affinity Ca^2+^ binding CD and EF sites. | High Affinity |  |
| **Rabbit (RPV)** | I).DKDKSGFIEEEE  II).DKDGDGKIGADE  I).DKDKSGFIEEDE  II).DKDGDGKIGVEE | α-parvalbumins from rabbit exhibit two independent and equivalent high- affinity Ca^2+^-Mg^2+^ sites. | High Affinity  High Affinity  High Affinity  High Affinity |  |
| **Rat (RTPV)** |  | Parvalbumins: Each of their two functional sites binds Ca (II) with an affinity of about 10^8^ M^-1^. |  |  |
| **Bovine cardics**  **(BCTNC)** | I).LGAEDGCISTKE  II).DEDGSGTVDFDE  III).DKNADGYIDLEE  IV).DKNNDGRIDYDE | The C-terminal peptide contains two Ca2+-binding sites. The third and fourth sites in cardiac-muscle troponin C are represented by the so-called high-affinity Ca^2+^/Mg^2+^- binding sites. | Low Affinity  Low Affinity  High Affinity  High Affinity |  |
| **Amphioxus** | I).DYNKDGSIQWED  II).DINKDDVVSWEE  III).DVSGDGIVDLEE | The two Amphioxus SCP's have three Ca-binding sites of high affinity: two calcium-specific ones and one Ca-Mg site. | **Low Affinity**  **Low Affinity**  **Low Affinity** |  |
| **Nereis** | I).DFDKDGAITRMD  II).DTNEDNNISRDE  III).DTNNDGLLSLEE | Ca2+ the three sites have the same intrinsic affinity (KCa = 1.7x10^8^ M-1) without co-operatively between the sites. | **Low Affinity**  **Low Affinity**  **Low Affinity** |  |
| **Rabbit(RSLC2)** | I).DQNRDGIIDKED  II).DPEGKGTIKKQF | Myosin contains two DTNB light chains and binds 2 mol of Ca (II) with high affinity. | **Low Affinity**  **Low Affinity** |  |
| **Scallop** | I).DVDRDGFVSKDD | Concluded that both RLC-a and RLC-b bind only one Ca^2+^ with similar affinities to each other. | Low Affinity |  |
| **Aequorin** | I).DVNHNGKISLDE  II).DKDQNGAITLDE  III).DIDESGQLDVDE | The Kca for one of the two Ca^2+^ is approx. 7x10^6^ M^-1^ | Low Affinity  High Affinity  Low Affinity |  |
| **Calcineurin B** | I).DLDNSGSLSVEE  II).DTDGNGEVDFKE  III).DMDKDGYISNGE  IV).DKDGDGRISFEE | Demonstrate that calcineurin is also a Ca^2+^-binding protein with a high affinity for Ca^2+^ (10^-6^ M) in the presence of physio-logical concentrations of Mg^2+^. | Low Affinity  Low Affinity  High Affinity  High Affinity |  |
| **Ca vector protein** | I).DANGDGVIDFDE  II).DEDGNGVIDIPE | CaVP binds 2 Ca^2+^ atoms in a non-cooperative way with intrinsic binding constant of 8.2x10^6^ forms a high affinity Ca^2+^-dependent complex. | High Affinity  High Affinity |  |
| **F. Hepatica FH8** | I).DRNGDGKVSAEE  II).DKNKDGKLDLKE | FH8 displays low affinity for Ca^2+^ | Low Binder  Low Binder |  |
| **Human S100A** | I).DANHDGRISFDE | Shows weak binding affinity for ca^2+^ One Ca^2+^-binding site with micromolar affinity | Low Binder |  |
| **Human Polycystin-2** | I).DQDGDQELTEHE |  | Low Binder |  |
| **Human Calnuc** | I).DINSDGVLDEQE  II).DTNQDRLVTLEE | Ca^2+^ binds with an affinity of 7 μM and causes structural changes. They showed that Ca^2+^ binds to both sites with equal affinity. | Low Binder  Low Binder |  |
| **Human Centrin3** | I).DTDKDEAIDYHE  II).DDDDSGKISLRN  III).DKDGDGEINQEE | Binds one Ca^2+^ with high and two Ca^2+^ with low aﬃnity. | Low Binder  Low Binder  High Binder |  |
| **Human Centrin2** | I).DRDGDGEVSEQE | Binds only one Ca^2+^ per molecule with a signiﬁcant aﬃnity | High Binder |  |
| **S. cerevisiae Centrin** | I).DMNNDGFLDYHE  II).DDDHTGKISIKN  III).DLDGDDEINENE | Cdc31 has one high aﬃnity Ca^2+^ - Mg ^2+^ and two lower aﬃnity Ca^2+^ sites. | Low Binder  Low Binder  High Binder |  |
| **Human Calsenilin** | I).DINKDGYITKEE  II).DRNQDGVVTIEE | Affinities for Ca^+2^ binding at these two sites are greater than 1 µM. | High Binder  High Binder |  |

Supplementary Table S5 Predictions of putative EF hand containing calcium binding protein and their calcium binding affinities from *E.Histolytica* proteome.

| **Protein** | Ca^2+^ Binding Sites | Predicted sites | SVM Scores | Predicted Ka |
| --- | --- | --- | --- | --- |
| **Cabp8 gi|169802036|gb|eal50453.2| ef-hand calcium-binding domain containing protein** | Site I | DEEHTGYIDISE | 0.27 | Low Affinity |
| **Cabp9 gi|56474642|gb|eal52004.1| ef-hand calcium-binding domain containing protein** | Site I | DLDKDGSVNVDE | 0.43 | Low Affinity |
|  | Site II | DLNDDGEIDIRQ | 0.25 | Low Affinity |
|  | Site III | DIKDQGKIGAPE | 0.14 | Low Affinity |
|  | Site IV | DQDLDGFISLKE | 0.55 | High Affinity |
| **Cabp10 gi|56472778|gb|EAL50237.1| calmodulin, putative** | Site I | DADGDKKIECME | 0.32 | Low Affinity |
|  | Site II | DPEEKGVIDSKE | 0.14 | Low Affinity |
| **Cabp11 gi|56472561|gb|eal50040.1| calcium-binding protein, putative** | Site I | DEDKDGYLKVRE | 0.28 | High Affinity |
|  | Site II | DQNKIGSITLTQ | 0.02 | Low Affinity |
| **Cabp12 gi|56474389|gb|eal51761.1| troponin-like protein, putative** | Site I | DTDHSGYLDIDE | 0.38 | Low Affinity |
|  | Site II | DENEDGKMDLNE | 0.29 | Low Affinity |
|  | Site III | DVNGDGVLDKKE | 0.42 | Low Affinity |
|  | Site IV | DTDKNGSLDFDE | 0.39 | Low Affinity |
| **Cabp13 gi|56471188|gb|eal48778.1| calcium-binding protein, putative** | Site I | DKDHSGTLEIDE | 0.31 | Low Affinity |
| **Cabp14 gi|56468461|gb|eal46305.1| EF-hand calcium-binding domain containing protein** | Site I | DTDRSGTIEINE | 0.49 | Low Affinity |
|  | Site II | DVDFNGRISFYE | 0.44 | High Affinity |
|  | Site III | DTNRSGTMEPHE | 0.16 | Low Affinity |
| **Cabp15 gi|56470174|gb|eal47854.1| grainin, putative** | Site I | DKDKSGTLELNE | 0.27 | Low Affinity |
|  | Site II | DMDLSGNIGFYE | 0.34 | Low Affinity |
|  | Site III | DADHSGTMDLNE | 0.30 | Low Affinity |
| **Cabp16 gi|56466987|gb|eal44984.1| grainin 1** | Site I | DKDKSGSLELDE | 0.26 | Low Affinity |
|  | Site II | DVDLSGSIGFYE | 0.35 | Low Affinity |
|  | Site III | DKDKSGNLDEQE | 0.30 | Low Affinity |
| **Cabp18 gi|169802082|gb|eal49043.2| actinin-like protein, putative** | Site I | DKDKSGTLELDE | 0.30 | Low Affinity |
|  | Site II | DADNNGSIGFYE | 0.47 | Low Affinity |
|  | Site III | DVDQSGSLDITE | 0.23 | Low Affinity |
| **Cabp19 gi|169800239|gb|eal42646.2| EF-hand calcium-binding domain containing protein** | Site I | DRDRSGTLEINE | 0.24 | Low Affinity |
|  | Site II | DTDFNGHISFYE | 0.49 | Low Affinity |
|  | Site III | DRNRSGTLEPHE | 0.14 | Low Affinity |
| **Cabp20 gi|56474807|gb|eal52163.1| calmodulin, putative** | Site I | DIDHDKKISRDQ | 0.04 | Low Affinity |
|  | Site II | DKEENGQIHEAE | 0.20 | Low Affinity |
| **Cabp21 gi|56467093|gb|eal45078.1| calcineurin b subunit, putative** | Site I | DVDNDGFISNPE | 0.61 | High Affinity |
|  | Site II | DKDRDGKISYEE | 0.76 | High Affinity |
| **Cabp22 gi|56467590|gb|eal45535.1| EF-hand calcium-binding domain containing protein** | Site I | DTNRTGKISFDV | 0.18 | Low Affinity |
|  | Site II | DVDNDGLLSYEE | 0.44 | High Affinity |
|  | Site III | DEDNSGSIEGEE | 0.45 | High Affinity |
| **Cabp23 gi|56465487|gb|eal43738.1| hypothetical protein, conserved** | Site I | DINGNGKISKEE | 0.63 | Low Affinity |
|  | Site II | DLNNDGKIPTDD | 0.31 | Low Affinity |
| **Cabp24 gi|56472021|gb|eal49541.1| hypothetical protein 40.t00032** | Site I | DKDKDELITIEE | 0.34 | High Affinity |
|  | Site II | DSNNDNKITCKE | 0.22 | Low Affinity |
|  | Site III | DLNQNGKIDIQE | 0.47 | Low Affinity |
|  | Site IV | DSDGDNELNLVE | 0.11 | Low Affinity |
|  | Site V | DIDGSGLIDRME | 0.56 | High Affinity |
| **Cabp25 gi|56467775|gb|eal45702.1| EF-hand calcium-binding domain containing protein** | Site I | DKDKDELITIEE | 0.34 | High Affinity |
|  | Site II | DSNNDNKITCKE | 0.22 | Low Affinity |
|  | Site III | DLNQNGKIDIQE | 0.47 | Low Affinity |
|  | Site IV | DSDGDNELNLVE | 0.11 | Low Affinity |
|  | Site V | DIDGSGLIDRME | 0.56 | High Affinity |
| **Cabp26 gi|56474460|gb|eal51827.1| EF-hand calcium-binding domain containing protein** | Site I | DSNGDGVLQIDE | 0.40 | Low Affinity |
|  | Site II | NINCDGYLDKEE | 0.10 | Low Affinity |
|  | Site III | DGDHDGLINSQE | 0.51 | High Affinity |
|  | Site IV | DKDYSKSIEYDE | 0.17 | Low Affinity |
| **Cabp27 gi|56473611|gb|eal51029.1| EF-hand calcium-binding domain containing protein** | Site I | DENGDGVLQLDE | 0.38 | Low Affinity |
|  | Site II | DENGDGVLQLDE | 0.38 | Low Affinity |
|  | Site III | DDNRNGLIDEDE | 0.46 | Low Affinity |
|  | Site IV | DTNRDGLLNETE | 0.27 | Low Affinity |

Supplementary Table S6. The performance and comparison of CAL-EF-AFi with PFAM and Calpred on E. *histolytica* proteome. Listed are the sequence predicted by CAL-EF-AFi followed by PFAM based HMM model prediction and CalPred’s predictions. (Legends for CAL-EF-AFi’s Prediction: number of Ca^2+^binding Loop sequence prediction, residue number followed by sequence and SVM scores; Legends for PFAM predictions red colored region is the loop region predicted, Followed by the Evalue for the sequence; Legends for CalPred predictions X: Non-Binding region C: Calcium Binding region).

**>GI|67477818|REF|XP_654345.1| CALCIUM-BINDING PROTEIN 1 (EHCBP1) [ENTAMOEBA HISTOLYTICA HM-1:IMSS]**

CAL-EF-AFi’s Prediction

| EF-LOOP1 9 DVNGDGAVSYEE SVM Score = 0.9641304 |
| --- |
| EF-LOOP2 45 DADGNGEIDQNE SVM Score = 0.9794917 |
| EF-LOOP3 84 DVDGDGKLTKEE SVM Score = 0.9573716 |
| EF-LOOP4 116 DANGDGYITLEE SVM Score = 0.99070771 |

PFAM based HMM model prediction

| gi|67477818|ref|XP_654345.1| 3 EALFKEIDVNGDGAVSYEEVKAFVSK 28 | 6.00E-009 |
| --- | --- |
| gi|67477818|ref|XP_654345.1| 38 LQLIFKSIDADGNGEIDQNEFAKFY 62 | 3.20E-008 |
| gi|67477818|ref|XP_654345.1| 77 LKVLYKLMDVDGDGKLTKEEVTSFFKK 103 | 1.30E-007 |
| gi|67477818|ref|XP_654345.1| 114 MKADANGDGYITLEEFLEF 132 | 1.10E-007 |

| Sequence | MAEALFKEIDVNGDGAVSYEEVKAFVSKKRAIKNEQLLQLIFKSIDADGNGEIDQNEFAK |
| --- | --- |
| SVM using PSSM model | CCCCCCCCCCCCCCCCCCCCCCCCCCCCCCCCCCCCCCCCCCCCCCCCCCCCCCCCCCCC |
| Sequence | FYGSIQGQDLSDDKIGLKVLYKLMDVDGDGKLTKEEVTSFFKKHGIEKVAEQVMKADANG |
| SVM using PSSM model | CCCCCCCCCCCCCCCCCCCCCCCCCCCCCCCCCCCCCCCCCCCCCCCCCCCCCCCCCCCC |
| Sequence | DGYITLEEFLEFSL |
| SVM using PSSM model | CCCCCCCCCCCCCC |

**CalPred’s predictions**

**>GI|67483720|REF|XP_657080.1| CALMODULIN [ENTAMOEBA HISTOLYTICA HM-1:IMSS]**

**CAL-EF-AFi’s Prediction**

| *EF-LOOP1 9 DANGDGSVSYEE SVM Score = 0.9717777* |
| --- |
| EF-LOOP2 45 DIDGNGEIDLAE SVM Score = 0.9730997 |
| EF-LOOP3 84 DADGDGKLTKEE SVM Score = 0.9638998 |
| EF-LOOP4 116 DANGDGYITLEE SVM Score = 0.99070771 |

**PFAM based HMM model prediction**

| *gi|67483720|ref|XP_657080.1| 3 EALFKQL****DANGDGSVSYEE****VKAFVSS 28* | *3.00E-008* |
| --- | --- |
| gi|67483720|ref|XP_657080.1| 38 LQLIFKAI**DIDGNGEIDLAE**FTKFA 62 | 3.80E-008 |
| gi|67483720|ref|XP_657080.1| 78 KILYKLM**DADGDGKLTKEE**VTTFFKKF 104 | 2.00E-007 |
| gi|67483720|ref|XP_657080.1| 113 IMKA**DANGDGYITLEE**FLA 131 | 1.00E-007 |

**CalPred’s predictions**

| *Sequence* | *MAEALFKQLDANGDGSVSYEEVKAFVSSKRPIKNEQLLQLIFKAIDIDGNGEIDLAEFTK* |
| --- | --- |
| SVM using PSSM model | CCCCCCCCCCCCCCCCCCCCCCCCCCCCCCCCCCCCCCCCCCCCCCCCCCCCCCCCCCCC |
| Sequence | FAAAVKEQDLSDEKVGLKILYKLMDADGDGKLTKEEVTTFFKKFGYEKVVDQIMKADANG |
| SVM using PSSM model | CCCCCCCCCCCCCCCCCCCCCCCCCCCCCCCCCCCCCCCCCCCCCCCCCCCCCCCCCCCC |
| Sequence | DGYITLEEFLAFNL |
| SVM using PSSM model | CCCCCCCCCCCCCC |

**>GI|67483860|REF|XP_657150.1| TROPONIN-LIKE PROTEIN [ENTAMOEBA HISTOLYTICA HM-1:IMSS]**

**CAL-EF-AFi’s Prediction**

| *EF-LOOP1 13 DTDHSGYLDIDE SVM Score = 0.8853862* |
| --- |
| EF-LOOP2 49 DENEDGKMDLNE SVM Score = 0.8162761 |
| EF-LOOP3 86 DVNGDGVLDKKE SVM Score = 0.9099356 |
| EF-LOOP4 122 DTDKNGSLDFDE SVM Score = 0.8901965 |

**PFAM based HMM model prediction**

| *gi|67483860|ref|XP_657150.1| 5 DIKHVFDMI****DTDHSGYLDIDE****FVKACSQ 32* | *2.50E-010* |
| --- | --- |
| gi|67483860|ref|XP_657150.1| 45 LFHMA**DENEDGKMDLNE**FKNMI 66 | 7.80E-007 |
| gi|67483860|ref|XP_657150.1| 82 LFKRC**DVNGDGVLDKKE**VFDILQS 105 | 2.10E-006 |
| gi|67483860|ref|XP_657150.1| 114 DINETFDIY**DTDKNGSLDFDE**YMKMVE 140 | 7.80E-009 |

**CalPred’s predictions**

| *Sequence* | *MTEADIKHVFDMIDTDHSGYLDIDEFVKACSQLIEGCEKESAVALFHMADENEDGKMDLN* |
| --- | --- |
| SVM using PSSM model | CCCCCCCCCCCCCCCCCCCCCCCCCCCCCCCCCCCCCCCCCCCCCCCCCCCCCCCCCCCC |
| Sequence | EFKNMIEFYLKNSEEEDPYVLLFKRCDVNGDGVLDKKEVFDILQSIDPNITIQDINETFD |
| SVM using PSSM model | CCCCCCCCCCCCCCCCCCCCCCCCCCCCCCCCCCCCCCCCCCCCCCCCCCCCCCCCCCCC |
| Sequence | IYDTDKNGSLDFDEYMKMVEDIKSQ |
| SVM using PSSM model | CCCCCCCCCCCCCCCCCCCCCCCCC |

**>GI|67465960|REF|XP_649138.1| EF-HAND CALCIUM-BINDING DOMAIN CONTAINING PROTEIN [ENTAMOEBA HISTOLYTICA HM-1:IMSS]**

CAL-EF-AFi’s Prediction

| *EF-LOOP1 13 DKDKSGYLSPDE SVM Score = 0.8889663* |
| --- |
| **No Hit Found** |
| **No Hit Found** |
| EF-LOOP2 99 DSNNSGKVNKKE SVM Score = 0.691678 |
| EF-LOOP3 135 DEDGDGKISFQE SVM Score = 0.9917197 |

PFAM based HMM model prediction

| *gi|67465960|ref|XP_649138.1| 6 IEFIFNLL****DKDKSGYLSPDE****FCEGLI 31* | *8.70E-010* |
| --- | --- |
| gi|67465960|ref|XP_649138.1| 48 FNDIFLLAD 56 | 9.5 |
| gi|67465960|ref|XP_649138.1| 64 KDNKITLREFKNICS 78 | 0.0064 |
| gi|67465960|ref|XP_649138.1| 95 AFRLV**DSNNSGKVNKKE**LTAFLKK 118 | 7.80E-008 |
| gi|67465960|ref|XP_649138.1| 127 EIDALMDSI**DEDGDGKISFQE**FMLL 151 | 3.70E-009 |

**CalPred’s predictions**

| *Sequence* | *MNNTQIEFIFNLLDKDKSGYLSPDEFCEGLIEFYHITDDKKESYREIFNDIFLLADGKGL* |
| --- | --- |
| SVM using PSSM model | CCCCCCCCCCCCCCCCCCCCCCCCCCCCCCCCCCCCCCCCCCCCCCCCCCCCCCCCCCCC |
| Sequence | FNSKDNKITLREFKNICSLLPTETKKTDVIIGTVAFRLVDSNNSGKVNKKELTAFLKKTG |
| SVM using PSSM model | CCCCCCCCCCCCCCCCCCCCCCCCCCCCCCCCCCCCCCCCCCCCCCCCCCCCCCCCCCCC |
| Sequence | VHLKKGEIDALMDSIDEDGDGKISFQEFMLLYDLN |
| SVM using PSSM model | CCCCCCCCCCCCCCCCCCCCCCCCCCCCCCCCCCC |

**>GI|67471513|REF|XP_651708.1| CALMODULIN [ENTAMOEBA HISTOLYTICA HM-1:IMSS]**

CAL-EF-AFi’s Prediction

| *EF-LOOP1 23 DKDNDNKLTAEE SVM Score = 0.675134* |
| --- |
| EF-LOOP2 59 DKDNSGKFDQET SVM Score = 0.45715 |
| EF-LOOP3 96 DKEKNGYISASE SVM Score = 0.90558 |
| **No Hit Found** |
| **No Hit Found** |

**PFAM based HMM model prediction**

| *gi|67471513|ref|XP_651708.1| 15 EYKEAFQLF****DKDNDNKLTAEE****LGTVMRAL 43* | *3.00E-010* |
| --- | --- |
| gi|67471513|ref|XP_651708.1| 52 ISEIVKDY**DKDNSGKFDQE**TFLTIM 76 | 2.00E-006 |
| gi|67471513|ref|XP_651708.1| 88 DIKKAFEIF**DKEKNGYISASE**LKHVLTTL 116 | 2.00E-010 |
| gi|67471513|ref|XP_651708.1| 119 KLTEQEVDDLLKE 131 | 9.9 |
| gi|67471513|ref|XP_651708.1| 137 GLINVDDFVKLITS 150 | 0.12 |

**CalPred’s predictions**

| *Sequence* | *MSEQKKVLTAEEQQEYKEAFQLFDKDNDNKLTAEELGTVMRALGANPTKQKISEIVKDYD* |
| --- | --- |
| SVM using PSSM model | CCCCCCCCCCCCCCCCCCCCCCCCCCCCCCCCCCCCCCCCCCCCCCCCCCCCCCCCCCCC |
| Sequence | KDNSGKFDQETFLTIMLEYGQEVDSTEDIKKAFEIFDKEKNGYISASELKHVLTTLGEKL |
| SVM using PSSM model | CCCCCCCCCCCCCCCCCCCCCCCCCCCCCCCCCCCCCCCCCCCCCCCCCCCCCCCCCCCC |
| Sequence | TEQEVDDLLKEIGVEEGLINVDDFVKLITSK |
| SVM using PSSM model | CCCCCCCCCCCCCCCCCCCCCCCCCCCCCCC |

**>GI|67483331|REF|XP_656940.1| EF-HAND CALCIUM-BINDING DOMAIN CONTAINING PROTEIN [ENTAMOEBA HISTOLYTICA HM-1:IMSS]**

CAL-EF-AFi’s Prediction

| *EF-LOOP1 14 DKTHSGRVSCDE SVM Score = 0.668888* |
| --- |
| EF-LOOP2 53 DVDNDNALNQKE SVM Score = 0.129422 |
| EF-LOOP3 100 DSDMNGTVSCSE SVM Score = 0.8395899 |
| EF-LOOP4 180 DNCQFGYINAKD SVM Score = 0.092114 |
| EF-LOOP5 263 DNNSNGELDGEE SVM Score = 0.720944 |

**PFAM based HMM model prediction**

| *gi|67483331|ref|XP_656940.1| 10 AFYIV****DKTHSGRVSCDE****VMNIM 31* | *8.90E-05* |
| --- | --- |
| gi|67483331|ref|XP_656940.1| 47 RFFFSLC**DVDNDNALNQKE**FEHFM 70 | 6.30E-05 |
| gi|67483331|ref|XP_656940.1| 94 IEIFRAI**DSDMNGTVSCSE**IAHTLSK 119 | 8.50E-06 |
| gi|67483331|ref|XP_656940.1| 175 ALFKKL**DNCQFGYINAKD**LVSHI 197 | 0.63 |
| gi|67483331|ref|XP_656940.1| 257 EAMFRLI**DNNSNGELDGEE**LVLLIE 281 | 5.00E-06 |
| gi|67483331|ref|XP_656940.1| 302 TITYSQFIDIMH 313 | 0.75 |

**CalPred’s predictions**

| *Sequence* | *MNFLFANKLAFYIVDKTHSGRVSCDEVMNIMIPLVGSTDPTTIDCMRFFFSLCDVDNDNA* |
| --- | --- |
| SVM using PSSM model | CCCCCCCCCCCCCCCCCCCCCCCCCCCCCCCCCCCCCCCCCCCCCCCCCCCCCCCCCCCC |
| Sequence | LNQKEFEHFMWCYNKCCEPKFVKIKADLVIVLFIEIFRAIDSDMNGTVSCSEIAHTLSKL |
| SVM using PSSM model | CCCCCCCCCCCCCCCCCCCCCCCCCCCCCCCCCCCCCCCCCCCCCCCCCCCCCCCCCCCC |
| Sequence | EESNVTMFGYKDMFEDIRDYAITKGPQTELTEFEFLCIAIKPEILFLHLEAKYNALFKKL |
| SVM using PSSM model | CCCCCCCCCCCCCCCCCCCCCCCCCCCCCCCCCCCCCCCCCCCCCCCCCCCCCCCCCCCC |
| Sequence | DNCQFGYINAKDLVSHIARFMFPRGCETKNALDLVISIITSTTQSEKIKIKQFGFLIELL |
| SVM using PSSM model | CCCCCCCCCCCCCCCCCCCCCCCCCCCCCCCCCCCCCCCCCCCCCCCCCCCCCCCCCCCC |
| Sequence | KDVSRSATYFTNPMLYEAMFRLIDNNSNGELDGEELVLLIETIKLSSKKKEIVMKAIKAK |
| SVM using PSSM model | CCCCCCCCCCCCCCCCCCCCCCCCCCCCCCCCCCCCCCCCCCCCCCCCCCCCCCCCCCCC |
| Sequence | STITYSQFIDIMHN |
| SVM using PSSM model | CCCCCCCCCCCCCC |

**>GI|183232350|REF|XP_648375.2| ACTININ-LIKE PROTEIN [ENTAMOEBA HISTOLYTICA HM-1:IMSS]**

CAL-EF-AFi’s Prediction

| *EF-LOOP1 301 DKDKSGTLELDE SVM Score = 0.8245922* |
| --- |
| EF-LOOP2 337 DADNNGSIGFYE SVM Score = 0.9354762 |
| EF-LOOP3 367 DVDQSGSLDITE SVM Score = 0.740728 |

**PFAM based HMM model prediction**

| *gi|183232350|ref|XP_648375.2| 293 ELQDWFKKI****DKDKSGTLELDE****LLK 316* | *5.10E-08* |
| --- | --- |
| gi|183232350|ref|XP_648375.2| 330 IKRLMLIF**DADNNGSIGFYE**FIALY 354 | 9.40E-07 |
| gi|183232350|ref|XP_648375.2| 363 TFKHF**DVDQSGSLDITE**LQVAL 384 | 7.20E-06 |

**CalPred’s predictions**

| *Sequence* | *MKEEKLNTSVSAENIVDGNRTYILGMVWTMILKYKINANQQKNVNAKEEVVENNALLDWV* |
| --- | --- |
| SVM using PSSM model | CCCCCCCCCCCCCCCCCCCCCCCCCCCCCCCCCCCCCCCCCCCCCCCCCCCCCCCCCCCC |
| Sequence | NSFGLNVSNFSSDWKDGVALVKLTEAVSAGQIKFEQFSGLDNTQMVIDCQKLAYEQFKIP |
| SVM using PSSM model | CCCCCCCCCCCCCCCCCCCCCCCCCCCCCCCCCCCCCCCCCCCCCCCCCCCCCCCCCCCC |
| Sequence | ILMDVKDLVCERPDPKSIMTYVSVYKERYEQLLVEKEQKEEQERIAREEQERKQKEEQER |
| SVM using PSSM model | CCCCCCCCCCCCCCCCCCCCCCCCCCCCCCCCCCCCCCCCCCCCCCCCCCCCCCCCCCCC |
| Sequence | LAREEQERLAREEQERLAREEQERLAREEQERKQKEEQERLAREEQERKQREEQERLNQQ |
| SVM using PSSM model | CCCCCCCCCCCCCCCCCCCCCCCCCCCCCCCCCCCCCCCCCCCCCCCCCCCCCCCCCCCC |
| Sequence | QPTSQQLTFFSVQAAADAWILQNIQAAYAQDPTIQFQWWYPLVQNLSANDFRELQDWFKK |
| SVM using PSSM model | CCCCCCCCCCCCCCCCCCCCCCCCCCCCCCCCCCCCCCCCCCCCCCCCCCCCCCCCCCCC |
| Sequence | IDKDKSGTLELDELLKAKWPKDMKMNNETIKRLMLIFDADNNGSIGFYEFIALYNWVKLC |
| SVM using PSSM model | CCCCCCCCCCCCCCCCCCCCCCCCCCCCCCCCCCCCCCCCCCCCCCCCCCCCCCCCCCCC |
| Sequence | VATFKHFDVDQSGSLDITELQVALPQLGFNLNKQSCDALIRANKNLIGKKKLNQVQFICA |
| SVM using PSSM model | CCCCCCCCCCCCCCCCCCCCCCCCCCCCCCCCCCCCCCCCCCCCCCCCCCCCCCCCCCCC |
| Sequence | TSYLAQCRTIYQLTFDTHRDQLDPVEFDKFINLVLGLI |
| SVM using PSSM model | CCCCCCCCCCCCCCCCCCCCCCCCCCCCCCCCCCCCCC |

**>GI|183229830|REF|XP_001913381.1| EF-HAND CALCIUM-BINDING DOMAIN CONTAINING PROTEIN [ENTAMOEBA HISTOLYTICA HM-1:IMSS]**

CAL-EF-AFi’s Prediction

| ***No Hit Found*** |
| --- |
| EF-LOOP1 54 DVDENKALDITE SVM Score = 0.095634 |
| EF-LOOP1 96 DSDGNASLSAEE SVM Score = 0.503052 |
| EF-LOOP2 175 DKDHSGMISVKE SVM Score = 0.929836 |
| EF-LOOP3 213 DETGKGEIDKNQ SVM Score = 0.413192 |
| EF-LOOP4 256 DSDKSGLLERDE SVM Score = 0.674488 |

**PFAM based HMM model prediction**

| *gi|183229830|ref|XP_001913381.1| 13 CDKDKDKLVRKEDL 26* | *1.3* |
| --- | --- |
| gi|183229830|ref|XP_001913381.1| 49 LFDLC**DVDENKALDITE**FLHLMKC 72 | 0.00034 |
| gi|183229830|ref|XP_001913381.1| 90 MALFRAI**DSDGNASLSAEE**ITKALDK 115 | 0.00071 |
| gi|183229830|ref|XP_001913381.1| 170 ALFEKG**DKDHSGMISVKE**LKTLFKQ 194 | 2.50E-07 |
| gi|183229830|ref|XP_001913381.1| 209 IIKLL**DETGKGEIDKNQ**FAT 228 | 0.004 |
| gi|183229830|ref|XP_001913381.1| 250 EAIFKIL**DSDKSGLLERDE**TTKLLKSM 276 | 1.60E-05 |

**CalPred’s predictions**

| *Sequence* | *MCEFAAHFVHGFCDKDKDKLVRKEDLFPLLIQFLQSEDPQYKIGFENYLFDLCDVDENKA* |
| --- | --- |
| SVM using PSSM model | CCCCCCCCCCCCCCCCCCCCCCCCCCCCCCCCCCCCCCCCCCCCCCCCCCCCCCCCCCCC |
| Sequence | LDITEFLHLMKCLEIFESQKGQSKNLTTMMALFRAIDSDGNASLSAEEITKALDKLPNDS |
| SVM using PSSM model | CCCCCCCCCCCCCCXXCCCCCCCCCCCCCCCCCCCCCCCCCCCCCCCCCCCCCCCCCCCC |
| Sequence | SVNEEIKSAVKKIKEYCGLLGGREINEMEFLGIVIPPSDFQQYNQQVFSALFEKGDKDHS |
| SVM using PSSM model | CCCCCCCCCCCCCCCCCCCCCCCCCCCCCCCCCCCCCCCCCCCCCCCCCCCCCCCCCCCC |
| Sequence | GMISVKELKTLFKQMYPDGVDERKAPEIIIKLLDETGKGEIDKNQFATFACAVAGNDVTI |
| SVM using PSSM model | CCCCCCCCCCCCCCCCCCCCCCCCCCCCCCCCCCCCCCCCCCCCCCCCCCCCCCCCCCCC |
| Sequence | RCLTKTRFYEAIFKILDSDKSGLLERDETTKLLKSMGLNEKKIAKWFEKIGEEPLTLNKF |
| SVM using PSSM model | CCCCCCCCCCCCCCCCCCCCCCCCCCCCCCCCCCCCCCCCCCCCCCCCCCCCCCCCCCCC |
| Sequence | LEIYLKK |
| SVM using PSSM model | CCCCCCC |

**>GI|183232352|REF|XP_654429.2| ACTININ-LIKE PROTEIN [ENTAMOEBA HISTOLYTICA HM-1:IMSS]**

CAL-EF-AFi’s Prediction

| *EF-LOOP1 365 DKDKSGTLELDE SVM Score = 0.8245922* |
| --- |
| EF-LOOP2 401 DADNNGSIGFYE SVM Score = 0.9354762 |
| EF-LOOP3 431 DVDQSGSLDITE SVM Score = 0.740728 |

**PFAM based HMM model prediction**

| *gi|183232352|ref|XP_654429.2| 357 ELQDWFKKI****DKDKSGTLELDE****LLK 380* | *6.00E-08* |
| --- | --- |
| gi|183232352|ref|XP_654429.2| 394 IKRLMLIF**DADNNGSIGFYE**FIALY 418 | 1.10E-06 |
| gi|183232352|ref|XP_654429.2| 427 TFKHF**DVDQSGSLDITE**LQVAL 448 | 8.50E-06 |
|  |  |

**CalPred’s predictions**

| *Sequence* | *MKEEKLNTSVSAENIVDGNRTYILGMVWTMILKYKINANQQKNVNAKEEVVENNALLDWV* |
| --- | --- |
| SVM using PSSM model | CCCCCCCCCCCCCCCCCCCCCCCCCCCCCCCCCCCCCCCCCCCCCCCCCCCCCCCCCCCC |
| Sequence | NSFGLNVSNFSSDWKDGVALVKLTEAVSAGQIKFEQFSGLDNTQMVIDCQKLAYEQFKIP |
| SVM using PSSM model | CCCCCCCCCCCCCCCCCCCCCCCCCCCCCCCCCCCCCCCCCCCCCCCCCCCCCCCCCCCC |
| Sequence | ILMDVKDLVCERPDPKSIMTYVSVYKERYEQLLVEKEQKEEQERIAREEQERKQKEEQER |
| SVM using PSSM model | CCCCCCCCCCCCCCCCCCCCCCCCCCCCCCCCCCCCCCCCCCCCCCCCCCCCCCCCCCCC |
| Sequence | LAREEQERLAREEQERLAREEQERLAREEQERKQKEEQERLAREEQERLAREEQERLARE |
| SVM using PSSM model | CCCCCCCCCCCCCCCCCCCCCCCCCCCCCCCCCCCCCCCCCCCCCCCCCCCCCCCCCCCC |
| Sequence | EQERKQKEEQERLAREEQERLAREEQERLAREEQERKQKEEQERLAREEQERKQREEQER |
| SVM using PSSM model | CCCCCCCCCCCCCCCCCCCCCCCCCCCCCCCCCCCCCCCCCCCCCCCCCCCCCCCCCCCC |
| Sequence | LNQQQPTSQQLTFFSVQAAADAWILQNIQAAYAQDPTIQFQWWYPLVQNLSANDFRELQD |
| SVM using PSSM model | CCCCCCCCCCCCCCCCCCCCCCCCCCCCCCCCCCCCCCCCCCCCCCCCCCCCCCCCCCCC |
| Sequence | WFKKIDKDKSGTLELDELLKAKWPKDMKMNNETIKRLMLIFDADNNGSIGFYEFIALYNW |
| SVM using PSSM model | CCCCCCCCCCCCCCCCCCCCCCCCCCCCCCCCCCCCCCCCCCCCCCCCCCCCCCCCCCCC |
| Sequence | VKLCVATFKHFDVDQSGSLDITELQVALPQLGFNLNKQSCDALIRANKNLIGKKKLNQVQ |
| SVM using PSSM model | CCCCCCCCCCCCCCCCCCCCCCCCCCCCCCCCCCCCCCCCCCCCCCCCCCCCCCCCCCCC |
| Sequence | FICATSYLAQCRTIYQLTFDTHRDQLDPVEFDKFINLVLGLI |
| SVM using PSSM model | CCCCCCCCCCCCCCCCCCCCCCCCCCCCCCCCCCCCCCCCCC |

**>GI|183234431|REF|XP_001914018.1| GRAININ [ENTAMOEBA HISTOLYTICA HM-1:IMSS]**

CAL-EF-AFi’s Prediction

| *EF-LOOP1 55 DKDKSGTLELTE SVM Score = 0.747126* |
| --- |
| EF-LOOP2 91 DVDFSGSISFYE SVM Score = 0.9128984 |
| EF-LOOP3 121 DVDKSGNLDLKE SVM Score = 0.68117 |

**PFAM based HMM model prediction**

| *gi|183234431|ref|XP_001914018.1| 48 LQNWFIKV****DKDKSGTLELTE****LRSA 71* | *3.30E-06* |
| --- | --- |
| gi|183234431|ref|XP_001914018.1| 84 CRHLMRIF**DVDFSGSISFYE**YLAMMK 109 | 1.30E-07 |
| gi|183234431|ref|XP_001914018.1| 117 VFRKY**DVDKSGNLDLKE**IQTAL 138 | 1.90E-06 |

**CalPred’s predictions**

| *Sequence* | *MSLFAIQQAADAWIVQNITATYQADENIKKEWWFPLACSISGTEFRNLQNWFIKVDKDKS* |
| --- | --- |
| SVM using PSSM model | CCCCCCCCCCCCCCCCCCCCCCCCCCCCCCCCCCCCCCCCCCCCCCCCCCCCCCCCCCCC |
| Sequence | GTLELTELRSARWPNGVKLDDETCRHLMRIFDVDFSGSISFYEYLAMMKFVELTTSVFRK |
| SVM using PSSM model | CCCCCCCCCCCCCCCCCCCCCCCCCCCCCCCCCCCCCCCCCCCCCCCCCCCCCCCCCCCC |
| Sequence | YDVDKSGNLDLKEIQTALPDLGFDLNTKSCQVITKLCGKGLFSKKIQLPQFIGCAAYLGQ |
| SVM using PSSM model | CCCCCCCCCCCCCCCCCCCCCCCCCCCCCCCCCCCCCCCCCCCCCCCCCCCCCCCCCCCC |
| Sequence | IRTIYQHGFKQAQGDFNRPVFAKFLNLVMSLVDDA |
| SVM using PSSM model | CCCCCCCCCCCCCCCCCCCCCCCCCCCCCCCCCCC |

**>GI|67475022|REF|XP_653241.1| GRAININ [ENTAMOEBA HISTOLYTICA HM-1:IMSS]**

CAL-EF-AFi’s Prediction

| *EF-LOOP1 55 DKDKSGTLELNE SVM Score = 0.786662* |
| --- |
| EF-LOOP2 91 DMDLSGNIGFYE SVM Score = 0.8548516 |
| EF-LOOP3 121 DADHSGTMDLNE SVM Score = 0.8208616 |

PFAM based HMM model prediction

| *gi|67475022|ref|XP_653241.1| 48 LQEWFEKT****DKDKSGTLELNE****LKS 70* | *6.00E-07* |
| --- | --- |
| gi|67475022|ref|XP_653241.1| 84 CRKLMKIF**DMDLSGNIGFYE**YLALMK 109 | 1.40E-05 |
| gi|67475022|ref|XP_653241.1| 114 VDKTFTHF**DADHSGTMDLNE**MMAAL 138 | 2.50E-07 |

**CalPred’s predictions**

| *Sequence* | *MSIFDIQAAQDAWIFQNITKTFQEDPLIKEQWWYPLCMSLRIEEFRPLQEWFEKTDKDKS* |
| --- | --- |
| SVM using PSSM model | CCCCCCCCCCCCCCCCCCCCCCCCCCCCCCCCCCCCCCCCCCCCCCCCCCCCCCCCCCCC |
| Sequence | GTLELNELKSAKFPGGLRLDEDTCRKLMKIFDMDLSGNIGFYEYLALMKFVDLVDKTFTH |
| SVM using PSSM model | CCCCCCCCCCCCCCCCCCCCCCCCCCCCCCCCCCCCCCCCCCCCCCCCCCCCCCCCCCCC |
| Sequence | FDADHSGTMDLNEMMAALPQLGFDITRKNCEALIRANARGLFSKKIKRSQFVGCVSFLGL |
| SVM using PSSM model | CCCCCCCCCCCCCCCCCCCCCCCCCCCCCCCCCCCCCCCCCCCCCCCCCCCCCCCCCCCC |
| Sequence | TRSIYQKAMNVKNENFKREDFSRFMNLVLLICDEL |
| SVM using PSSM model | CCCCCCCCCCCCCCCCCCCCCCCCCCCCCCCCCCC |

**>gi|67470235|ref|XP_651088.1| EF-hand calcium-binding domain containing protein [Entamoeba histolytica HM-1:IMSS]**

CAL-EF-AFi’s Prediction

| *EF-LOOP1 11 DKDKDELITIEE SVM Score = 0.8605703* |
| --- |
| EF-LOOP2 48 DSNNDNKITCKE SVM Score = 0.723732 |
| **No Hit Found** |
| EF-LOOP3 173 DLNQNGKIDIQE SVM Score = 0.934142 |
| EF-LOOP4 211 DSDGDNELNLVE SVM Score = 0.503644 |
| EF-LOOP5 235 DFSKGGEITKDH SVM Score = 0.14966 |
| EF-LOOP6 256 DIDGSGLIDRME SVM Score = 0.9623305 |
| **No Hit Found** |

**PFAM based HMM model prediction**

| *gi|67470235|ref|XP_651088.1| 6 NVFKE****IDKDKDELITIEE*** *23* | *3.80E-05* |
| --- | --- |
| gi|67470235|ref|XP_651088.1| 46 LII**DSNNDNKITCKE**FKQFFK 66 | 8.70E-06 |
| gi|67470235|ref|XP_651088.1| 104 TNKKGEITTEQILNW 118 | 0.49 |
| gi|67470235|ref|XP_651088.1| 168 NVFKEI**DLNQNGKIDIQE**IMY 188 | 8.10E-07 |
| gi|67470235|ref|XP_651088.1| 207 VIKLL**DSDGDNELNLVE**FVKYC 228 | 0.00054 |
| gi|67470235|ref|XP_651088.1| 234 LF**DFSKGGEITKDH**MLMVLY 253 | 0.81 |
| gi|67470235|ref|XP_651088.1| 253 YELV**DIDGSGLIDRME**MERYF 273 | 0.00032 |
| gi|67470235|ref|XP_651088.1| 295 GVIDFEVFKYL 305 | 3.8 |

**CalPred’s predictions**

| *Sequence* | *MISPTNVFKEIDKDKDELITIEEALKGVHYEIEEKEFKEFEENVLLIIDSNNDNKITCKE* |
| --- | --- |
| SVM using PSSM model | CCCCCCCCCCCCCCCCCCCCCCCCCCCCCCCCCCCCCCCCCCCCCCCCCCCCCCCCCCCC |
| Sequence | FKQFFKIIQIAQNIKICQKMNYTNIQIPFPETEKLKLYFRCFKTNKKGEITTEQILNWSR |
| SVM using PSSM model | CCCCCCCCCCCCCCCCCCCCCCCCCCCCCCCCCCCCCCCCCCCCCCCCCCCCCCCCCCCC |
| Sequence | KIKDPQQQVFIETSLRNINSNKCNEQLFTKCFISPDGYIMEYCPKYINVFKEIDLNQNGK |
| SVM using PSSM model | CCCCCCCCCCCCCCCCCCCCCCCCCCCCCCCCCCCCCCCCCCCCCCCCCCCCCCCCCCCC |
| Sequence | IDIQEIMYHISRIFTVPENMRDFFTIVIKLLDSDGDNELNLVEFVKYCLALNHLFDFSKG |
| SVM using PSSM model | CCCCCCCCCCCCCCCCCCCCCCCCCCCCCCCCCCCCCCCCCCCCCCCCCCCCCCCCCCCC |
| Sequence | GEITKDHMLMVLYELVDIDGSGLIDRMEMERYFNILHTPFETQETVRNIMKQCGGVIDFE |
| SVM using PSSM model | CCCCCCCCCCCCCCCCCCCCCCCCCCCCCCCCCCCCCCCCCCCCCCCCCCCCCCCCCCCC |
| Sequence | VFKYLYS |
| SVM using PSSM model | CCCCCCC |

**>GI|67483972|REF|XP_657206.1| EF-HAND CALCIUM-BINDING DOMAIN CONTAINING PROTEIN [ENTAMOEBA HISTOLYTICA HM-1:IMSS]**

CAL-EF-AFi’s Prediction

| *EF-LOOP1 120 GVDSNGLYSIDE SVM Score = 0.125214* |
| --- |
| **No Hit Found** |
| EF-LOOP2 280 DSNGDGVLQIDE SVM Score = 0.8998945 |
| EF-LOOP3 406 NINCDGYLDKEE SVM Score = 0.473998 |
| EF-LOOP4 448 DGDHDGLINSQE SVM Score = 0.9494684 |
| **No Hit Found** |
| EF-LOOP5 532 DKDYSKSIEYDE SVM Score = 0.625546 |
| EF-LOOP6 572 DVDENKSLDPNE SVM Score = 0.135982 |
| EF-LOOP7 647 DLNKKDFIQINE SVM Score = 0.08426 |

**PFAM based HMM model prediction**

| ***No Hit Found*** |  |
| --- | --- |
| gi|67483972|ref|XP_657206.1| 241 FRLLDNEYNGFLTLQKYSRFLQ 262 | 1.2 |
| gi|67483972|ref|XP_657206.1| 273 VKKSIKEL**DSNGDGVLQIDE**FIHV 296 | 1.20E-05 |
| gi|67483972|ref|XP_657206.1| 402 IFNVG**NINCDGYLDKE**EFIILMT 424 | 0.00049 |
| gi|67483972|ref|XP_657206.1| 444 FFHCV**DGDHDGLINSQE**VS 462 | 0.0022 |
| gi|67483972|ref|XP_657206.1| 477 EANDLIRTYDEAGKKELNLEEYLCYFM 503 | 0.52 |
| gi|67483972|ref|XP_657206.1| 525 IAKTFRLI**DKDYSKSIEYDE**VLNYV 549 | 0.0011 |
| gi|67483972|ref|XP_657206.1| 566 TMFYVC**DVDENKSLDPNE**FFQFY 588 | 0.016 |
| gi|67483972|ref|XP_657206.1| 642 NFMIKY**DLNKKDFIQINE**FVLA 663 | 0.01 |

**CalPred’s predictions**

| *Sequence* | *MAREIFIERFNKLSRTSTYESITNDINDYFFSKKSNFIEQLIPAIVKEKYDVIKYTRICQ* |
| --- | --- |
| SVM using PSSM model | CCCCCCCCCCCCCCCCCCCCCCCCCCCCCCCCCCCCCCCCCCCCCCCCCCCCCCCCCCCC |
| Sequence | FIDAFIFKYKTFTVAHIAAIVFLLLDPSKNLKVKRSDTSKYFTKTSKANSKKLKRLEKET |
| SVM using PSSM model | CCCCCCCCCCCCCCCCCCCCCCCCCCCCCCCCCCCCCCCCCCCCCCCCCCCCCCCCCCCC |
| Sequence | GVDSNGLYSIDEFISLYENVNEVQLDSSTVKQCILDWSFKSLFDQYAKGKSYISDSDVLE |
| SVM using PSSM model | CCCCCCCCCCCCCCCCCCCCCCCCCCCCCCCCCCCCCCCCCCCCCCCCCCCCCCCCCCCC |
| Sequence | GLQKQFKIKEHVNALKVVIRLMDTSRVHKFKLYEFNELCLQIAFVEQKVGINIFSLNVIS |
| SVM using PSSM model | CCCCCCCCCCCCCCCCCCCCCCCCCCCCCCCCCCCCCCCCCCCCCCCCCCCCCCCCCCCC |
| Sequence | FRLLDNEYNGFLTLQKYSRFLQKSGFDIKKDNVKKSIKELDSNGDGVLQIDEFIHVSNGL |
| SVM using PSSM model | CCCCCCCCCCCCCCCCCCCCCCCCCCCCCCCCCCCCCCCCCCCCCCCCCCCCCCCCCCCC |
| Sequence | CTSKIPDSFFNNLRARNQTDLKYDSEQPQNMRTTIQILRNDNIKKEKNCNLIRTSITSDE |
| SVM using PSSM model | CCCCCCCCCCCCCCCCCCCCCCCCCCCCCCCCCCCCCCCCCCCCCCCCCCCCCCCCCCCC |
| Sequence | YSKLFDELCPSHKMNMDSLRSALITVIGETNTQALRTALPVIFNVGNINCDGYLDKEEFI |
| SVM using PSSM model | CCCCCCCCCCCCCCCCCCCCCCCCCCCCCCCCCCCCCCCCCCCCCCCCCCCCCCCCCCCC |
| Sequence | ILMTPIGKCMKNKVITERDMYEVFFHCVDGDHDGLINSQEVSFLVQQIDGDTLTEEEAND |
| SVM using PSSM model | CCCCCCCCCCCCCCCCCCCCCCCCCCCCCCCCCCCCCCCCCCCCCCCCCCCCCCCCCCCC |
| Sequence | LIRTYDEAGKKELNLEEYLCYFMQLDEIKLDDSEEIKQEKMKRQIAKTFRLIDKDYSKSI |
| SVM using PSSM model | CCCCCCCCCCCCCCCCCCCCCCCCCCCCCCCCCCCCCCCCCCCCCCCCCCCCCCCCCCCC |
| Sequence | EYDEVLNYVINTVGQISQTNKICLSTMFYVCDVDENKSLDPNEFFQFYYLFEYYVDENGE |
| SVM using PSSM model | CCCCCCCCCCCCCCCCCCCCCCCCCCCCCCCCCCCCCCCCCCCCCCCCCCCCCCCCCCCC |
| Sequence | FDYVSIYVELFDMLDVKNEKILPRYIVDKLLEKIGRPESILNFMIKYDLNKKDFIQINEF |
| SVM using PSSM model | CCCCCCXXCXCCCCCCCCCCCCCCCCCCCCCCCCCCCCCCCCCCCCCCCCCCCCCCCCCC |
| Sequence | VLAFCGKSLDDGI |
| SVM using PSSM model | CCCCCCCCCCCCC |

**>GI|67468658|REF|XP_650357.1| GRAININ 2 [ENTAMOEBA HISTOLYTICA HM-1:IMSS]**

CAL-EF-AFi’s Prediction

| *EF-LOOP1 55 DKDKSGTLEIGE SVM Score = 0.783276* |
| --- |
| EF-LOOP2 91 DIDMSGSIGFFE SVM Score = 0.867242 |
| EF-LOOP3 121 DADKSGSLDVNE SVM Score = 0.780676 |

PFAM based HMM model prediction

| *gi|67468658|ref|XP_650357.1| 48 LQSWFISV****DKDKSGTLEIGE****LKKA 71* | *3.30E-05* |
| --- | --- |
| gi|67468658|ref|XP_650357.1| 84 IKRLMRVF**DIDMSGSIGFFE**FLALW 108 | 1.50E-06 |
| gi|67468658|ref|XP_650357.1| 114 CNETFKHF**DADKSGSLDVNE**LIKAL 138 | 3.80E-08 |

CalPred’s predictions

| *Sequence* | *MSLFAIQAAADAFVTQMIQAAVNSDPNLKFQWWFPLVERLDAKDLQNLQSWFISVDKDKS* |
| --- | --- |
| SVM using PSSM model | CCCCCCCCCCCCCCCCCCCCCCCCCCCCCCCCCCCCCCCCCCCCCCCCCCCCCCCCCCCC |
| Sequence | GTLEIGELKKAKFPGGIKVDDKTIKRLMRVFDIDMSGSIGFFEFLALWNFMNLCNETFKH |
| SVM using PSSM model | CCCCCCCCCCCCCCCCCCCCCCCCCCCCCCCCCCCCCCCCCCCCCCCCCCCCCCCCCCCC |
| Sequence | FDADKSGSLDVNELIKALPMLGFNCNKRSVDVLLKMNGSSLGSKKVSKNQFISTAAYLGQ |
| SVM using PSSM model | CCCCCCCCCCCCCCCCCCCCCCCCCCCCCCCCCCCCCCCCCCCCCCCCCCCCCCCCCCCC |
| Sequence | CRSIYQKTFNMKREEIDNAEFDKFVNLVLALSG |
| SVM using PSSM model | CCCCCCCCCCCCCCCCCCCCCCCCCCCCCCCCC |

**>GI|67480543|REF|XP_655621.1| CALMODULIN [ENTAMOEBA HISTOLYTICA HM-1:IMSS]**

CAL-EF-AFi’s Prediction

| ***No Hit Found*** |
| --- |
| EF-LOOP1 60 DADGDKKIECME SVM Score = 0.84389 |
| EF-LOOP2 97 DPEEKGVIDSKE SVM Score = 0.58013 |

**PFAM based HMM model prediction**

| *gi|67480543|ref|XP_655621.1| 16 ELKDAFDMF****DSSKKGYLDKDD****VKKLFKT 43* | *2.30E-07* |
| --- | --- |
| gi|67480543|ref|XP_655621.1| 52 DLDAAFKEA**DADGDKKIECME**FINMMT 78 | 2.30E-08 |
| gi|67480543|ref|XP_655621.1| 90 LTEAFKVF**DPEEKGVIDSKE**LTEAL 114 | 1.30E-05 |

CalPred’s predictions

| *Sequence* | *MSRRRDEEVDQELVGELKDAFDMFDSSKKGYLDKDDVKKLFKTNGIRVTDEDLDAAFKEA* |
| --- | --- |
| SVM using PSSM model | CCCCCCCCCCCCCCCCCCCCCCCCCCCCCCCCCCCCCCCCCCCCCCCCCCCCCCCCCCCC |
| Sequence | DADGDKKIECMEFINMMTGKMKTASTEQKLTEAFKVFDPEEKGVIDSKELTEALLNIGER |
| SVM using PSSM model | CCCCCCCCCCCCCCCCCCCCCCCCCCCCCCCCCCCCCCCCCCCCCCCCCCCCCCCCCCCC |
| Sequence | CTTSEVGELKTVAENQEGQIRYELFIQAVFAKK |
| SVM using PSSM model | CCCCCCCCCCCCCCCCCCCCCCCCCCCCCCCCC |

**>GI|67468717|REF|XP_650372.1| GRAININ 1 [ENTAMOEBA HISTOLYTICA HM-1:IMSS]**

CAL-EF-AFi’s Prediction

| *EF-LOOP1 55 DKDKSGSLELDE SVM Score = 0.775198* |
| --- |
| EF-LOOP2 91 DVDLSGSIGFYE SVM Score = 0.8683236 |
| EF-LOOP3 121 DKDKSGNLDEQE SVM Score = 0.8243236 |

**PFAM based HMM model prediction**

| *gi|67468717|ref|XP_650372.1| 47 ELQDFFVKT****DKDKSGSLELDE****LKKA 71* | *4.40E-06* |
| --- | --- |
| gi|67468717|ref|XP_650372.1| 84 CRHLMRIF**DVDLSGSIGFYE**YLALMK 109 | 8.10E-06 |
| gi|67468717|ref|XP_650372.1| 117 VFTKF**DKDKSGNLDEQE**IYAAL 138 | 3.00E-06 |

**CalPred’s predictions**

| *Sequence* | *MSLFAIQAAADAWVAQHITAAYQADPLIQREWWYPLATSISGTEFKELQDFFVKTDKDKS* |
| --- | --- |
| SVM using PSSM model | CCCCCCCCCCCCCCCCCCCCCCCCCCCCCCCCCCCCCCCCCCCCCCCCCCCCCCCCCCCC |
| Sequence | GSLELDELKKAKFPGGIKLDENACRHLMRIFDVDLSGSIGFYEYLALMKFVKLATAVFTK |
| SVM using PSSM model | CCCCCCCCCCCCCCCCCCCCCCCCCCCCCCCCCCCCCCCCCCCCCCCCCCCCCCCCCCCC |
| Sequence | FDKDKSGNLDEQEIYAALPELGFDLNMKACKILIQLCGKGLLTKKIQISQFISCAAYLGQ |
| SVM using PSSM model | CCCCCCCCCCCCCCCCCCCCCCCCCCCCCCCCCCCCCCCCCCCCCCCCCCCCCCCCCCCC |
| Sequence | IRTIYQHGFKQAQGDFNRATFSKFMNLTLMLMDDA |
| SVM using PSSM model | CCCCCCCCCCCCCCCCCCCCCCCCCCCCCCCCCCC |

**>GI|67484340|REF|XP_657390.1| EF-HAND CALCIUM-BINDING DOMAIN CONTAINING PROTEIN [ENTAMOEBA HISTOLYTICA HM-1:IMSS]**

CAL-EF-AFi’s Prediction

| *EF-LOOP1 18 DLDKDGSVNVDE SVM Score = 0.9131896* |
| --- |
| EF-LOOP2 57 DLNDDGEIDIRQ SVM Score = 0.762758 |
| EF-LOOP3 96 DIKDQGKIGAPE SVM Score = 0.568634 |
| EF-LOOP4 132 DQDLDGFISLKE SVM Score = 0.9602737 |

PFAM based HMM model prediction

| *gi|67484340|ref|XP_657390.1| 11 IEEFFAEI****DLDKDGSVNVDE****YFNGVK 36* | *4.10E-07* |
| --- | --- |
| gi|67484340|ref|XP_657390.1| 53 LFHLA**DLNDDGEIDIRQ**FARLI 74 | 0.00028 |
| gi|67484340|ref|XP_657390.1| 91 AVFRLL**DIKDQGKIGAPE**LERLLKKM 116 | 0.00051 |
| gi|67484340|ref|XP_657390.1| 125 IEGFMDQV**DQDLDGFISLKE**FLAHF 149 | 8.70E-06 |

CalPred’s predictions

| *Sequence* | *MPEDKPQGKTIEEFFAEIDLDKDGSVNVDEYFNGVKMWRNDITDDDKPSQALLFHLADLN* |
| --- | --- |
| SVM using PSSM model | CCCCCCCCCCCCCCCCCCCCCCCCCCCCCCCCCCCCCCCCCCCCCCCCCCCCCCCCCCCC |
| Sequence | DDGEIDIRQFARLIAILNRGFGKDIKSVFTAVFRLLDIKDQGKIGAPELERLLKKMGLQI |
| SVM using PSSM model | CCCCCCCCCCCCCCCCCCCCCCCCCCCCCCCCCCCCCCCCCCCCCCCCCCCCCCCCCCCC |
| Sequence | ESDDIEGFMDQVDQDLDGFISLKEFLAHFVKESKDGQ |
| SVM using PSSM model | CCCCCCCCCCCCCCCCCCCCCCCCCCCCCCCCCCCCC |

**>GI|67468945|REF|XP_650464.1| CALCINEURIN B SUBUNIT [ENTAMOEBA HISTOLYTICA HM-1:IMSS]**

CAL-EF-AFi’s Prediction

| ***No Hit Found*** |
| --- |
| EF-LOOP1 105 DVDNDGFISNPE SVM Score = 0.9735738 |
| EF-LOOP2 146 DKDRDGKISYEE SVM Score = 0.98974648 |

**PFAM based HMM model prediction**

| *gi|67468945|ref|XP_650464.1| 26 ELRRLFRR 33* | *3.4* |
| --- | --- |
| gi|67468945|ref|XP_650464.1| 98 LRFAFQVY**DVDNDGFISNPE**LFKVLKM 124 | 9.20E-09 |

**CalPred’s predictions**

| *Sequence* | *MGNTLSGLKQEDVEQMMATTNFTESELRRLFRRFKKLGMKAKEGASEEYDDLAELTSNPV* |
| --- | --- |
| SVM using PSSM model | CCCCCCCCCCCCCCCCCCCCCCCCCCCCCCCCCCCCCCCCCCCCCCCCCCCCCCCCCCCC |
| Sequence | LQRLLEIFNRYENEEVQFSQFVATLSTLSDKGSQEAKLRFAFQVYDVDNDGFISNPELFK |
| SVM using PSSM model | CCCCCCCCCCCCCCCCCCCCCCCCCCCCCCCCCCCCCCCCCCCCCCCCCCCCCCCCCCCC |
| Sequence | VLKMIIGSSFTDEQLQQVVDKTIIEADKDRDGKISYEEFCSIILKNSENIGEKLTINWQ |
| SVM using PSSM model | CCCCCCCCCCCCCCCCCCCCCCCCCCCCCCCCCCCCCCCCCCCCCCCCCCCCCCCCCCC |

**>GI|67482131|REF|XP_656415.1| EF-HAND CALCIUM-BINDING DOMAIN CONTAINING PROTEIN [ENTAMOEBA HISTOLYTICA HM-1:IMSS]**

**CAL-EF-AFi’s Prediction**

| *EF-LOOP1 130 DENGDGVLQLDE SVM Score = 0.88277* |
| --- |
| **No Hit Found** |
| EF-LOOP2 294 DENGDGVLQLDE SVM Score = 0.88277 |
| EF-LOOP3 398 DIHNQGFITKTQ SVM Score = 0.03084 |
| EF-LOOP4 473 DDNRNGLIDEDE SVM Score = 0.9307262 |
| EF-LOOP5 520 DTNRDGLLNETE SVM Score = 0.788812 |

**PFAM based HMM model prediction**

| *gi|67482131|ref|XP_656415.1| 123 IKEYIKQN****DENGDGVLQLDE****FLGAF 147* | *0.00016* |
| --- | --- |
| gi|67482131|ref|XP_656415.1| 255 FRTLDKSCSASLDNNDLTRMLKA 277 | 0.98 |
| gi|67482131|ref|XP_656415.1| 291 INDL**DENGDGVLQLDE**MLSILK 312 | 0.00014 |
| gi|67482131|ref|XP_656415.1| 391 FKFLFFVA**DIHNQGFITKTQ**FSLIMK 416 | 0.013 |
| gi|67482131|ref|XP_656415.1| 472 MY**DDNRNGLIDEDE**FVYLMTE 492 | 1.10E-05 |
| gi|67482131|ref|XP_656415.1| 515 KLFRVY**DTNRDGLLNETE**VGEIF 537 | 1.80E-05 |

**CalPred’s predictions**

| *Sequence* | *MSRESFIIQFNGASSMIKKNKIPVARCVNLTMETFSNKTPNCEELLSMLWRITSKSDDVS* |
| --- | --- |
| SVM using PSSM model | CCCCCCCCCCCCCCCCCCCCCCCCCCCCCCCCCCCCCCCCCCCCCCCCCCCCCCCCCCCC |
| Sequence | VETFVKTMQHLDNLYFKTGELNGQVIITAAFFSLDSSYDYSLDSKEVSDFFKRIGDKKNS |
| SVM using PSSM model | CCCCCCCCCCCCCCCCCCCCCCCCCCCCCCCCCCCCCCCCCCCCCCCCCCCCCCCCCCCC |
| Sequence | KKIKEYIKQNDENGDGVLQLDEFLGAFDSIYKINSIPINDYLKVIEFTDFSKRYDLLPKK |
| SVM using PSSM model | CCCCCCCCCCCCCCCCCCCCCCCCCCCCCCCCCCCCCCCCCCCCCCCCCCCCCCCCCCCC |
| Sequence | KGKALQEDVVQELIKDIPLNERKGIETQLSVLIFLSSKDKKTISREEYVKVRREINYIKT |
| SVM using PSSM model | CCCCCCCCCCCCCCCCCCCCCCCCCCCCCCCCCCCCCCCCCCCCCCCCCCCCCCCCCCCC |
| Sequence | KIGRITTEVLMTCTFRTLDKSCSASLDNNDLTRMLKASGMESKKKVVLQYINDLDENGDG |
| SVM using PSSM model | CCCCCCCCCCCCCCCCCCCCCCCCCCCCCCCCCCCCCCCCCCCCCCCCCCCCCCCCCCCC |
| Sequence | VLQLDEMLSILKVFVNKHNFDIEKYLKNLDARTPADIVECSFKEPIKIPNNNFIVNDHLT |
| SVM using PSSM model | CCCCCCCCCCCCCCCCCCCCCCCCCCCCCCCCCCCCCCCCCCCCCCCCCCCCCCCCCCCC |
| Sequence | SNDSEQITFALFDATIKCKLGESYSTSQETFKFLFFVADIHNQGFITKTQFSLIMKFLDS |
| SVM using PSSM model | CCCCCCCCCCCCCCCCCCCCCCCCCCCCCCCCCCCCCCCCCCCCCCCCCCCCCCCCCCCC |
| Sequence | TNGKIENDPALCRICFQLQGKKEIGVDDLQTLCSKMGQPFSSEQEAINELVMYDDNRNGL |
| SVM using PSSM model | CCCCCCCCCCCCCCCCCCCCCCCCCCCCCCCCCCCCCCCCCCCCCCCCCCCCCCCCCCCC |
| Sequence | IDEDEFVYLMTEDDELKMDDSIEEHLRKNARKAIKLFRVYDTNRDGLLNETEVGEIFIAQ |
| SVM using PSSM model | CCCCCCCCCCCCCCCCCCCCCCCCCCCCCCCCCCCCCCCCCCCCCCCCCCCCCCCCCCCC |
| Sequence | WHQCSPNNQKAIHIGFLKNQQNDFIDENRFVVLCQELEASMNEDDSGEINIDKLLTNFFY |
| SVM using PSSM model | CCCCCCCCCCCCCCCCCCCCCCCCCCCCCCCCCCCCCCCCCCCCCCCCCCCCCCCCCCCX |
| Sequence | FYVPNGSNLMDKETLEKVLIQFKLPSSPVLIQKLLTSSNSFNMITFENFSKYISQHLQ |
| SVM using PSSM model | XCCCCCCCCCCCCCCCCCCCCCCCCCCCCCCCCCCCCCCCCCCCCCCCCCCCCCCCCC |

**>GI|183235954|REF|XP_648032.2| EF-HAND CALCIUM-BINDING DOMAIN CONTAINING PROTEIN [ENTAMOEBA HISTOLYTICA HM-1:IMSS]**

CAL-EF-AFi’s Prediction

| *EF-LOOP1 67 DRDRSGTLEINE SVM Score = 0.749556* |
| --- |
| EF-LOOP2 103 DTDFNGHISFYE SVM Score = 0.9413556 |
| EF-LOOP3 133 DRNRSGTLEPHE SVM Score = 0.58264 |

**PFAM based HMM model prediction**

| *gi|183235954|ref|XP_648032.2| 60 IYQWFMGV****DRDRSGTLEINE****LM 81* | *6.20E-05* |
| --- | --- |
| gi|183235954|ref|XP_648032.2| 97 LRMMRIF**DTDFNGHISFYE**FMAMYK 121 | 6.30E-09 |
| gi|183235954|ref|XP_648032.2| 128 NLFVMN**DRNRSGTLEPHE**ILPALQ 151 | 0.0018 |

**CalPred’s predictions**

| *Sequence* | *MQFGYPPMQPPVANFCLWNLQPIQGSWMGAACIYQMPPSVRNTWWFPLLNTIPLDQYTRI* |
| --- | --- |
| SVM using PSSM model | CCCCXCCCXCXXCCXXCCCCCCCCCCCCCCCCCCCCCCCCCCCCCCCCCCCCCCCCCCCC |
| Sequence | YQWFMGVDRDRSGTLEINELMMGQFPGGIRLSPQTALRMMRIFDTDFNGHISFYEFMAMY |
| SVM using PSSM model | CCCCCCCCCCCCCCCCCCCCCCCCCCCCCCCCCCCCCCCCCCCCCCCCCCCCCCCCCCCC |
| Sequence | KFMELAYNLFVMNDRNRSGTLEPHEILPALQQLGFYINQRTSLLLHRLFARGMAFCDLNC |
| SVM using PSSM model | CCCCCCCCCCCCCCCCCCCCCCCCCCCCCCCCCCCCCCCCCCCCCCCCCCCCCCCCCCCC |
| Sequence | WIAICAFAAQTRSAYQMIFMNPYYGPMKPFNPMEFGKFLDVVTSLLE |
| SVM using PSSM model | CCCCCCCCCCCCCCCCCCCCCCCXCCCCCCCCCCCCCCCCCCCCCCC |

**>GI|67469899|REF|XP_650921.1| EF-HAND CALCIUM-BINDING DOMAIN CONTAINING PROTEIN [ENTAMOEBA HISTOLYTICA HM-1:IMSS]**

CAL-EF-AFi’s Prediction

| *EF-LOOP1 17 DTNRTGKISFDV SVM Score = 0.661328* |
| --- |
| EF-LOOP2 56 DVDNDGLLSYEE SVM Score = 0.9224257 |
| **No Hit Found** |
| **No Hit Found** |
| **No Hit Found** |
| EF-LOOP3 266 DEDNSGSIEGEE SVM Score = 0.9235164 |

**PFAM based HMM model prediction**

| *gi|67469899|ref|XP_650921.1| 12 FAFYSL****DTNRTGKISFDV****L 30* | *0.00023* |
| --- | --- |
| gi|67469899|ref|XP_650921.1| 54 MLC**DVDNDGLLSYE**EFLHLF 73 | 2.50E-06 |
| gi|67469899|ref|XP_650921.1| 104 DRKCQGYVERDDLVYALRKL 123 | 0.15 |
| gi|67469899|ref|XP_650921.1| 138 FQKLINYCDSKGKGT 152 | 8.9 |
| gi|67469899|ref|XP_650921.1| 191 FMTKQEFSNYLK 202 | 3.9 |
| gi|67469899|ref|XP_650921.1| 262 FFRLV**DEDNSGSIEGEE**LTMMVNA 285 | 3.50E-06 |

**CalPred’s predictions**

| *Sequence* | *MSVLKSSFGTRFAFYSLDTNRTGKISFDVLNKYIYNLLSIEEDADKELCIFLCMLCDVDN* |
| --- | --- |
| SVM using PSSM model | CCCCCCCCCCCCCCCCCCCCCCCCCCCCCCCCCCCCCCCCCCCCCCCCCCCCCCCCCCCC |
| Sequence | DGLLSYEEFLHLFYCYSIHTQKDFFKIQVDIIIAVFIKLSRGTDRKCQGYVERDDLVYAL |
| SVM using PSSM model | CCCCCCCCCCCCCCCCCCCCCCCCCCCCCCCCCCCCXXXCXCCCCCCCCCCCCCCCCCCC |
| Sequence | RKLKSYDFKILNQYDAIFQKLINYCDSKGKGTTLSEYEFITYVVKPEYMNLHYSSHYETL |
| SVM using PSSM model | CCCCCCCCCCCCCCCCCCCCCCCCCCCCCCCCCCCCCCCCCCCCCCCCCCCCCCCCCCCC |
| Sequence | FNKWIKGKKDFMTKQEFSNYLKFRFPDGIGVKKSDEIIFTLIATDDKFTKTDLNMFGRLV |
| SVM using PSSM model | CCCCCCCCCCCCCCCCCCCCCCCCCCCCCCCCCCCCCCCCCCCCCCCCCCCCCCCCCCCC |
| Sequence | SLLNDLCGKSHCLDKNQCYELFFRLVDEDNSGSIEGEELTMMVNALKLGLVKNIKLLKEV |
| SVM using PSSM model | CCCCCCCCCCCCCCCCCCCCCCCCCCCCCCCCCCCCCCCCCCCCCCCCCCCCCCCCCCCC |
| Sequence | AEGTSLTKEQFVKYLTK |
| SVM using PSSM model | CCCCCCCCCCCCCCCCC |

**>GI|183230806|REF|XP_001913488.1| CALMODULIN [ENTAMOEBA HISTOLYTICA HM-1:IMSS]**

CAL-EF-AFi’s Prediction

| *EF-LOOP1 17 DKDQDGFITIDE SVM Score = 0.98682387* |
| --- |
| EF-LOOP2 53 NPDADGYIDYKA SVM Score = 0.386706 |

**PFAM based HMM model prediction**

| *gi|183230806|ref|XP_001913488.1| 10 LIEAFYNF****DKDQDGFITIDE****LKSILMD 36* | *3.00E-10* |
| --- | --- |
| gi|183230806|ref|XP_001913488.1| 45 EADEAVKEA**NPDADGYIDYKA**FARFLV 71 | 3.00E-05 |

**CalPred’s predictions**

| *Sequence* | *MSLENEISQLIEAFYNFDKDQDGFITIDELKSILMDQGMCISNEEADEAVKEANPDADGY* |
| --- | --- |
| SVM using PSSM model | CCCCCCCCCCCCCCCCCCCCCCCCCCCCCCCCCCCCCCCCCCCCCCCCCCCCCCCCCCCC |
| Sequence | IDYKAFARFLVEACRE |
| SVM using PSSM model | CCCCCCCCCCCCCCCC |

**>GI|67478507|REF|XP_654645.1| CALMODULIN [ENTAMOEBA HISTOLYTICA HM-1:IMSS]**

**CAL-EF-AFi’s Prediction**

| *EF-LOOP1 17 DKDQDGFITIDE SVM Score = 0.98682387* |
| --- |
| EF-LOOP2 53 NPDADGYIDYKA SVM Score = 0.386706 |

**PFAM based HMM model prediction**

| *gi|67478507|ref|XP_654645.1| 10 LIEAFYNF****DKDQDGFITIDE****LKSILMD 36* | *3.00E-10* |
| --- | --- |
| gi|67478507|ref|XP_654645.1| 45 EADEAVKEA**NPDADGYIDYKA**FARFLV 71 | 3.00E-05 |

**CalPred’s predictions**

| *Sequence* | *MSLENEISQLIEAFYNFDKDQDGFITIDELKSILMDQGMCISNEEADEAVKEANPDADGY* |
| --- | --- |
| SVM using PSSM model | CCCCCCCCCCCCCCCCCCCCCCCCCCCCCCCCCCCCCCCCCCCCCCCCCCCCCCCCCCCC |
| Sequence | IDYKAFARFLVEACRE |
| SVM using PSSM model | CCCCCCCCCCCCCCCC |

**>GI|67471481|REF|XP_651692.1| EF-HAND CALCIUM-BINDING DOMAIN CONTAINING PROTEIN [ENTAMOEBA HISTOLYTICA HM-1:IMSS]**

CAL-EF-AFi’s Prediction

| *EF-LOOP1 104 DTDRSGTIEINE SVM Score = 0.9423979* |
| --- |
| EF-LOOP2 140 DVDFNGRISFYE SVM Score = 0.9221756 |
| EF-LOOP3 170 DTNRSGTMEPHE SVM Score = 0.616102 |

**PFAM based HMM model prediction**

| *gi|67471481|ref|XP_651692.1| 100 WFLSV****DTDRSGTIEINE****LMM 119* | *1.70E-05* |
| --- | --- |
| gi|67471481|ref|XP_651692.1| 135 RLMRIF**DVDFNGRISFYE**YMGM 156 | 6.70E-06 |
| gi|67471481|ref|XP_651692.1| 165 NVFIQC**DTNRSGTMEPHE**IIPALR 188 | 0.0022 |

**CalPred’s predictions**

| *Sequence* | *MYGYPVGGMNLSGYPMNGICPIQPMGSVQVGVPVITNTPGMVTSVPVSALCLWNLVPSSN* |
| --- | --- |
| SVM using PSSM model | CXXCCCCCCCCCCCCCCCCCCCCCCCCCCCCCCCCCCCCCCCCCCCCCCCCCCCCCCCCC |
| Sequence | SWMNSRVQCQIMLDPMLRNNWWWNLVQSVSVTQFNSVYMWFLSVDTDRSGTIEINELMMA |
| SVM using PSSM model | CCCCCCCCCCCCCCCCCCCCCCCCCCCCCCCCCCCCCCCCCCCCCCCCCCCCCCCCCCCC |
| Sequence | QFPGGITLTPQTALRLMRIFDVDFNGRISFYEYMGMHKFLEICYNVFIQCDTNRSGTMEP |
| SVM using PSSM model | CCCCCCCCCCCCCCCCCCCCCCCCCCCCCCCCCCCCCCCCCCCCCCCCCCCCCCCCCCCC |
| Sequence | HEIIPALRILGFFVNQRTAIVLHRLFAHGSTICDLNCWIALCAFAAQTRTAYQLITMNPY |
| SVM using PSSM model | CCCCCCCCCCCCCCCCCCCCCCCCCCCCCCCCCCCCCCCCCCCCCCCCCCCCCCCCCCCC |
| Sequence | YGIIQPFNPIEFGKFLDIITSLLE |
| SVM using PSSM model | CCCCCCCCCCCCCCCCCCCCCCCC |

**>GI|67465932|REF|XP_649124.1| HYPOTHETICAL PROTEIN [ENTAMOEBA HISTOLYTICA HM-1:IMSS]**

CAL-EF-AFi’s Prediction

| *EF-LOOP1 71 DINGNGKISKEE SVM Score = 0.9754222* |
| --- |
| EF-LOOP2 113 DLNNDGKIPTDD SVM Score = 0.8284777 |
| **No Hit Found** |

**PFAM based HMM model prediction**

| *gi|67465932|ref|XP_649124.1| 72* ***DINGNGKISKEE****FKRT 87* | *1.70E-06* |
| --- | --- |
| gi|67465932|ref|XP_649124.1| 109 LFCIL**DLNNDGKIPTDD**MICFIVK 132 | 3.90E-05 |
| gi|67465932|ref|XP_649124.1| 148 SIIDPSHKGYVELKDFIKAMN 168 | 0.0017 |

**CalPred’s predictions**

| *Sequence* | *MNSTSNENTFKILAQGNDYLSIEATVSAINNVIYSASHPEETHGPSRKELLSSFFNSAIT* |
| --- | --- |
| SVM using PSSM model | CCCCCCCCCCCCCCCCCCCCCCCCCCCCCCCCCCCCCCCCCCCCCCCCCCCCCCCCCCCC |
| Sequence | TLYTPLMAVATDINGNGKISKEEFKRTLDIIQQYGLNKNSELNLANAVLFCILDLNNDGK |
| SVM using PSSM model | CCCCCCCCCCCCCCCCCCCCCCCCCCCCCCCCCCCCCCCCCCCCCCXCCCCCCCCCCCCC |
| Sequence | IPTDDMICFIVKMSQQQPSEESKEEVRSIIDPSHKGYVELKDFIKAMNM |
| SVM using PSSM model | CCCCCCCCCCCCCCCCCCCCCCCCCCCCCCCCCCCCCCCCCCCCCCCCC |

**>GI|67472489|REF|XP_652048.1| CALMODULIN [ENTAMOEBA HISTOLYTICA HM-1:IMSS]**

**CAL-EF-AFi’s Prediction**

| *EF-LOOP1 15 DGDGDGYLTLNE SVM Score = 0.9560923* |
| --- |
| EF-LOOP2 86 DKDKKGFIPAIE SVM Score = 0.8996835 |

**PFAM based HMM model prediction**

| *gi|67472489|ref|XP_652048.1| 7 DLKESFLLF****DGDGDGYLTLNE****FESLVR 33* | *1.60E-08* |
| --- | --- |
| gi|67472489|ref|XP_652048.1| 78 EIKTAINVL**DKDKKGFIPAIE**LRRILST 105 | 3.40E-06 |
| gi|67472489|ref|XP_652048.1| 114 EITDLFTFMGIDEQGVVKVDDFIN 137 | 1.2 |

**CalPred’s predictions**

| *Sequence* | *MQKHNEDLKESFLLFDGDGDGYLTLNEFESLVRVLGVVMETSAIASTYNSNSKVRGMSYE* |
| --- | --- |
| SVM using PSSM model | CCCCCCCCCCCCCCCCCCCCCCCCCCCCCCCCCCCCCCCCCCCCCCCCCCCCCCCCCCCC |
| Sequence | LFTSCFSQLKTKSFNKDEIKTAINVLDKDKKGFIPAIELRRILSTIGDNMEQKEITDLFT |
| SVM using PSSM model | CCCCCCCCCCCCCCCCCCCCCCCCCCCCCCCCCCCCCCCCCCCCCCCCCCCCCCCCCCCC |
| Sequence | FMGIDEQGVVKVDDFINQLMTVFK |
| SVM using PSSM model | CCCCCCCCCCCCCCCCCCCCCCCC |

**>GI|67480815|REF|XP_655757.1| CALMODULIN [ENTAMOEBA HISTOLYTICA HM-1:IMSS]**

**CAL-EF-AFi’s Prediction**

| *EF-LOOP1 22 DRDYDGKIDVKQ SVM Score = 0.8325622* |
| --- |
| EF-LOOP2 95 DQDKDGKIKASD SVM Score = 0.788838 |

**PFAM based HMM model prediction**

| *gi|67480815|ref|XP_655757.1| 15 IRDCFNFY****DRDYDGKIDVKQ****LGTLIRS 41* | *5.50E-07* |
| --- | --- |
| gi|67480815|ref|XP_655757.1| 88 LRKAFEVF**DQDKDGKIKASD**LA 109 | 2.00E-07 |

**CalPred’s predictions**

| *Sequence* | *MSMEIEAPNANTQKIRDCFNFYDRDYDGKIDVKQLGTLIRSLGCAPTEDEVNSYIKEFAI* |
| --- | --- |
| SVM using PSSM model | CCCCCCCCCCCCCCCCCCCCCCCCCCCCCCCCCCCCCCCCCCCCCCCCCCCCCCCCCCCC |
| Sequence | EGETFQIEQFELIMEREQSKPDTREIKLRKAFEVFDQDKDGKIKASDLAHNLTTVGDKMT |
| SVM using PSSM model | CCCCCCCCCCCCCCCCCCCCCCCCCCCCCCCCCCCCCCCCCCCCCCCCCCCCCCCCCCCC |
| Sequence | KEEVEKVFSILGITMESDIDLATFLKLVAL |
| SVM using PSSM model | CCCCCCCCCCCCCCCCCCCCCCCCCCCCCC |

**>GI|67473196|REF|XP_652365.1| CALMODULIN [ENTAMOEBA HISTOLYTICA HM-1:IMSS]**

CAL-EF-AFi’s Prediction

| *EF-LOOP1 19 DKDKDGQITFEE SVM Score = 0.98883979* |
| --- |
| **No Hit Found** |
| **No Hit Found** |
| **No Hit Found** |

**PFAM based HMM model prediction**

| *gi|67473196|ref|XP_652365.1| 12 FKATFKHY****DKDKDGQITFEE****LGQILRG 38* | *1.80E-11* |
| --- | --- |
| gi|67473196|ref|XP_652365.1| 81 EIQKAFDTF 89 | 4.3 |
| gi|67473196|ref|XP_652365.1| 96 TITSDEFKAMMME 108 | 0.0045 |
| gi|67473196|ref|XP_652365.1| 117 EADELIKDIGLNKEGCIDVKAFID 140 | 0.059 |

**CalPred’s predictions**

| *Sequence* | *MSSLTEEQKKKFKATFKHYDKDKDGQITFEELGQILRGMGRNTTDVEIYQMQQIHGSDKI* |
| --- | --- |
| SVM using PSSM model | CCCCCCCCCCCCCCCCCCCCCCCCCCCCCCCCCCCCCCCCCCCCCCCCCCCCCCCCCCCC |
| Sequence | DEATYLALLAQKLQEPDSVEEIQKAFDTFFGPGKTTITSDEFKAMMMEFGERVSEDEADE |
| SVM using PSSM model | CCCCCCCCCCCCCCCCCCCCCCCCCCCCCCCCCCCCCCCCCCCCCCCCCCCCCCCCCCCC |
| Sequence | LIKDIGLNKEGCIDVKAFIDHVFESK |
| SVM using PSSM model | CCCCCCCCCCCCCCCCCCCCCCCCCC |

**>GI|67480135|REF|XP_655426.1| CALCIUM-BINDING PROTEIN [ENTAMOEBA HISTOLYTICA HM-1:IMSS]**

CAL-EF-AFi’s Prediction

| ***No Hit Found*** |
| --- |
| EF-LOOP1 496 DEDKDGYLKVRE SVM Score = 0.799528 |
| EF-LOOP2 568 DQNKIGSITLTQ SVM Score = 0.263562 |
| EF-LOOP3 604 DYDKSKTLSYKE SVM Score = 0.035242 |

**PFAM based HMM model prediction**

| *gi|67480135|ref|XP_655426.1| 164 IYDILDIDKKGFL 176* | *1.5* |
| --- | --- |
| gi|67480135|ref|XP_655426.1| 489 IESIFNRF**DEDKDGYLKVRE**FRTAI 513 | 8.10E-07 |
| gi|67480135|ref|XP_655426.1| 564 IYNFI**DQNKIGSITLTQ**FINFA 585 | 0.077 |
| gi|67480135|ref|XP_655426.1| 596 ELTNLFNSF**DYDKSKTLSYKE**FYRMIQ 622 | 1.40E-05 |

**CalPred’s predictions**

| *Sequence* | *MSEERYDEIQLALQHIEVNWNEMGQRIQSFNSYIEQANTTKEFKKIAGEASDLIGDLDES* |
| --- | --- |
| SVM using PSSM model | CCCCCCCCCCCCCCCCCCCCCCCCCCCCCCCCCCCCCCCCCCCCCCCCCCCCCCCCCCCC |
| Sequence | EKLRTFISMTKIDIELTILEAKNLAVSDLKRSDPYVVFMANKEKYKTKVIENVLDPVWNE |
| SVM using PSSM model | CCCCCCCCCCCCCCCCCCCCCCCCCCCCCCCCCCCCCCCCCCCCCCCCCCCCCCCCCCCC |
| Sequence | SFQIKVEVGDKLMLQIMDKDVGKKDDENGVCYWKIPSMYSGQIIYDILDIDKKGFLYIKA |
| SVM using PSSM model | CCCCCCCCCCCCCCCCCCCCCCCCCCCCCCCCCCCCCCCCCCCCCCCCCCCCCCCCCCCC |
| Sequence | VCNNSPLQKRLMPFDYEKITLLEVCIISVNGFYNSLISNTCIKDITKPKVLKHLKYTMEL |
| SVM using PSSM model | CCCCCCCCCCCCCCCCCCCCCCCCCCCCCCCCCCCCCCCCCCCCCCCCCCCCCCCCCCCC |
| Sequence | TTSKTKCQRLIKKKTVEGQQYGNYVFFDQRIFVKGTVNDEIKISFLAKEKHQKKFKVITN |
| SVM using PSSM model | CCCCCCCCCCCCCCCCCCCCCCCCCCCCCCCCCCCCCCCCCCCCCCCCCCCCCCCCCCCC |
| Sequence | SSFIIPDFLPNEIMTLPIPLSESGSLQLKITCLESVYTNVYPTVIPTADMVIEKKGNVVM |
| SVM using PSSM model | CCCCCCCCCCCCCCCCCCCCCCCCCCCCCCCCCCCCCCCCCCCCCCCCCCCCCCCCCCCC |
| Sequence | KLTKSNGFGDFLAPYATVQFGPLLITTSPIQHSGEPVIFNESFLALVETGTKAIVRMYNK |
| SVM using PSSM model | CCCCCCCCCCCCCCCCCCCCCCCCCCCCCCCCCCCCCCCCCCCCCCCCCCCCCCCCCCCC |
| Sequence | DMKNPSKEGKKLCEGKFEVPEIKNKPVKVVVRFNSISQLEIEFFLIRTTSSVFDELEKTN |
| SVM using PSSM model | CCCCCCCCCCCCCCCCCCCCCCCCCCCCCCCCCCCCCCCCCCCCCCCCCCCCCCCCCCCC |
| Sequence | RIEKRNQKIESIFNRFDEDKDGYLKVREFRTAIQFVNSTLTRSLSVFLFPCFAVNNQINL |
| SVM using PSSM model | CCCCCCCCCCCCCCCCCCCCCCCCCCCCCCCCCCCCCCCCCCCCCCCCCCCCCCCCCCCC |
| Sequence | TIFKEVTEVLASFNNSHEAITKYIYNFIDQNKIGSITLTQFINFAHQTQIADSDKELTNL |
| SVM using PSSM model | CCCCCCCCCCCCCCCCCCCCCCCCCCCCCCCCCCCCCCCCCCCCCCCCCCCCCCCCCCCC |
| Sequence | FNSFDYDKSKTLSYKEFYRMIQCVVID |
| SVM using PSSM model | CCCCCCCCCCCCCCCCCCCCCCCCCCC |

**>GI|183233359|REF|XP_001913849.1| GRAININ 2 [ENTAMOEBA HISTOLYTICA HM-1:IMSS]**

CAL-EF-AFi’s Prediction

| *EF-LOOP1 4 DIDMSGSIGFFE SVM Score = 0.867242* |
| --- |
| EF-LOOP2 34 DADKSGSLDVNE SVM Score = 0.780676 |
| **No Hit Found** |
| **No Hit Found** |

**PFAM based HMM model prediction**

| *gi|183233359|ref|XP_001913849.1| 1 MRVF****DIDMSGSIGFFE****FLALW 21* | *3.20E-05* |
| --- | --- |
| gi|183233359|ref|XP_001913849.1| 27 CNETFKHF**DADKSGSLDVNE**LIKAL 51 | 1.70E-08 |
| gi|183233359|ref|XP_001913849.1| 75 GSKKVSKNQFI 85 | 7.2 |
| gi|183233359|ref|XP_001913849.1| 109 IDNAEFDK 116 | 8.7 |

**CalPred’s predictions**

**All The residues Predicted as Ca^2+^binding residues**

**>GI|67483988|REF|XP_657214.1| CALMODULIN [ENTAMOEBA HISTOLYTICA HM-1:IMSS]**

CAL-EF-AFi’s Prediction

| *EF-LOOP1 19 DKDKDGQITFEE SVM Score = 0.98883979* |
| --- |
| **No Hit Found** |
| **No Hit Found** |
| **No Hit Found** |

**PFAM based HMM model prediction**

| *gi|67483988|ref|XP_657214.1| 12 FKATFKHY****DKDKDGQITFEE****LGQILRG 38* | *1.80E-11* |
| --- | --- |
| gi|67483988|ref|XP_657214.1| 81 EIQKAFDTF 89 | 4.3 |
| gi|67483988|ref|XP_657214.1| 96 TITSDEFKAMMME 108 | 0.0045 |
| gi|67483988|ref|XP_657214.1| 117 EADELIKDTGLNKEGCIDVKAFID 140 | 0.33 |

**CalPred’s predictions**

**All The residues Predicted as Ca^2+^binding residues**

**>GI|67484714|REF|XP_657577.1| CALMODULIN [ENTAMOEBA HISTOLYTICA HM-1:IMSS]**

CAL-EF-AFi’s Prediction

| *EF-LOOP1 14 DIDHDKKISRDQ SVM Score = 0.32915* |
| --- |
| EF-LOOP2 86 DKEENGQIHEAE SVM Score = 0.690808 |
| **No Hit Found** |

**PFAM based HMM model prediction**

| *gi|67484714|ref|XP_657577.1| 7 IDDYFAIL****DIDHDKKISRDQ****VIELLRA 33* | *1.50E-05* |
| --- | --- |
| gi|67484714|ref|XP_657577.1| 82 VFTAL**DKEENGQIHEAE**LRQILS 104 | 0.00018 |
| gi|67484714|ref|XP_657577.1| 128 GYVDYKEFADMLVK 141 | 0.0022 |

**CalPred’s predictions**

**All The residues Predicted as Ca^2+^binding residues**

**>GI|67481165|REF|XP_655932.1| CALCIUM BINDING FAMILY PROTEIN [ENTAMOEBA HISTOLYTICA HM-1:IMSS]**

CAL-EF-AFi’s Prediction

| *EF-LOOP1 14 DIDHDKKISRDQ SVM Score = 0.32915* |
| --- |
| EF-LOOP2 86 DKEENGQIHEAE SVM Score = 0.690808 |
| **No Hit Found** |

| *gi|67481165|ref|XP_655932.1| 7 IDDYFSIL****DIDHDKKISRDQ****VIELLRA 33* | *1.50E-05* |
| --- | --- |
| gi|67481165|ref|XP_655932.1| 82 VFTAL**DKEENGQIHEAE**LRQILS 104 | 0.00018 |
| gi|67481165|ref|XP_655932.1| 128 GYVDYKEFADMLVK 141 | 0.0022 |

**CalPred’s predictions**

**All The residues Predicted as Ca^2+^binding residues**

**>GI|183232824|REF|XP_652247.2| HYPOTHETICAL PROTEIN [ENTAMOEBA HISTOLYTICA HM-1:IMSS]**

CAL-EF-AFi’s Prediction

| *EF-LOOP1 203 DKDHSGTLEIDE SVM Score = 0.8342437* |
| --- |
| EF-LOOP2 239 DTSRTGHLNIYE SVM Score = 0.086124 |

**PFAM based HMM model prediction**

| *gi|183232824|ref|XP_652247.2| 197 IAWFNQV****DKDHSGTLEIDE****I* *216* | *2.30E-05* |
| --- | --- |
| gi|183232824|ref|XP_652247.2| 234 RFMRIF**DTSRTGHLNIYE**FIAIY 256 | 0.00015 |

**CalPred’s predictions**

**All The residues Predicted as Ca^2+^binding residues**

**>GI|183230263|REF|XP_654169.2| HYPOTHETICAL PROTEIN [ENTAMOEBA HISTOLYTICA HM-1:IMSS]**

CAL-EF-AFi’s Prediction

| *EF-LOOP1 107 DKKKEGGEEEEE SVM Score = 0.178468* |
| --- |
| EF-LOOP2 714 DKDHSGTLEIDE SVM Score = 0.8342437 |
| EF-LOOP3 750 DTSRTGHLNIYE SVM Score = 0.086124 |

**PFAM based HMM model prediction**

| ***No Hit Found*** |  |
| --- | --- |
| gi|183230263|ref|XP_654169.2| 708 IAWFNQV**DKDHSGTLEIDE**I 727 | 6.60E-05 |
| gi|183230263|ref|XP_654169.2| 745 RFMRIF**DTSRTGHLNIYE**FIAIY 767 | 0.00044 |

**CalPred’s predictions**

**All The residues Predicted as Ca^2+^binding residues**

**>GI|183232460|REF|XP_655839.2| EF-HAND CALCIUM-BINDING DOMAIN CONTAINING PROTEIN [ENTAMOEBA HISTOLYTICA HM-1:IMSS]**

CAL-EF-AFi’s Prediction

| *EF-LOOP1 15 DEEHTGYIDISE SVM Score = 0.7852* |
| --- |
| **No Hit Found** |
| **No Hit Found** |

**PFAM based HMM model prediction**

| *gi|183232460|ref|XP_655839.2| 10 AVFNEL****DEEHTGYIDISE****YYA 30* | *0.00013* |
| --- | --- |
| gi|183232460|ref|XP_655839.2| 45 KALFKISDIQKNNRIDLKQFTELV 68 | 0.0036 |
| gi|183232460|ref|XP_655839.2| 129 KLNYQEYMSCA 139 | 5.9 |

**CalPred’s predictions**

**All The residues Predicted as Ca^2+^binding residues**

**>GI|67475126|REF|XP_653283.1| CALPONIN HOMOLOGY DOMAIN PROTEIN [ENTAMOEBA HISTOLYTICA HM-1:IMSS]**

**CAL-EF-AFi’s Prediction**

EF-LOOP1 496 DGNHDGILDKLE SVM Score = 0.768098

**PFAM based HMM model prediction**

| *gi|67475126|ref|XP_653283.1| 488 EFKQSFDAF****DGNHDGILDKLE****FRSCLSSM 516* | *1.10E-06* |
| --- | --- |

**CalPred’s predictions**

**All The residues Predicted as Ca^2+^binding residues**

**>GI|67463294|REF|XP_648304.1| HYPOTHETICAL PROTEIN [ENTAMOEBA HISTOLYTICA HM-1:IMSS]**

CAL-EF-AFi’s Prediction

| *EF-LOOP1 214 DRTGAGTFLRKE SVM Score = 0.297374* |
| --- |
| EF-LOOP2 420 DDDKDGFLSFNG SVM Score = 0.573178 |

**PFAM based HMM model prediction**

| *gi|67463294|ref|XP_648304.1| 207 LKYFFDKF****DRTGAGT*** *221* | *0.69* |
| --- | --- |
| gi|67463294|ref|XP_648304.1| 413 LKTLFSKL**DDDKDGFLSFNG**FIKIFEK 439 | 1.10E-05 |

**CalPred’s predictions**

**All The residues Predicted as Ca^2+^binding residues**

**>GI|183233916|REF|XP_652322.2| HYPOTHETICAL PROTEIN [ENTAMOEBA HISTOLYTICA HM-1:IMSS]**

CAL-EF-AFi’s Prediction

| *EF-LOOP1 18 DFDGDGKIGMDD SVM Score = 0.9000438* |
| --- |
| EF-LOOP2 151 DIDRDSQLNIIE SVM Score = 0.16314 |

**PFAM based HMM model prediction**

| *gi|183233916|ref|XP_652322.2| 11 VDFLFKIA****DFDGDGKIGMDD****AQ 32* | *0.0004* |
| --- | --- |
| gi|183233916|ref|XP_652322.2| 144 IDELMKLS**DIDRDSQLNIIE**FFIAMA 169 | 0.0076 |

**CalPred’s predictions**

**All The residues Predicted as Ca^2+^ binding residues**

**>GI|67471097|REF|XP_651503.1| HYPOTHETICAL PROTEIN [ENTAMOEBA HISTOLYTICA HM-1:IMSS]**

**CAL-EF-AFi’s Prediction**

EF-LOOP1 128 DINKDNALNFDE SVM Score = 0.287632

**PFAM based HMM model prediction**

| *gi|67471097|ref|XP_651503.1| 126 LKF****DINKDNALNFDE****WGK 143* | *0.00073* |
| --- | --- |

**CalPred’s predictions**

**All The residues Predicted as Ca^2+^binding residues**

**>GI|67480015|REF|XP_655377.1| RAB FAMILY GTPASE [ENTAMOEBA HISTOLYTICA HM-1:IMSS]**

CAL-EF-AFi’s Prediction

| *EF-LOOP1 122 DLDNSRVITKEE SVM Score = 0.627094* |
| --- |
| EF-LOOP2 168 PRNEKGLIDPDE SVM Score = 0.368722 |

**PFAM based HMM model prediction**

| *gi|67480015|ref|XP_655377.1| 121 KC****DLDNSRVITKEE*** *134* | *0.058* |
| --- | --- |
| gi|67480015|ref|XP_655377.1| 161 FDQIARKL**PRNEKGLIDPDE**V 181 | 0.071 |

**CalPred’s predictions**

**All The residues Predicted as Ca^2+^binding residues**

**Supplementary** **References**

1. Moeschler HJ, Schaer JJ, Cox JA (1980) A thermodynamic analysis of the binding of calcium and magnesium ions to parvalbumin. Eur J Biochem 111: 73-78.

2. Linse S, Helmersson A, Forsen S (1991) Calcium binding to calmodulin and its globular domains. J Biol Chem 266: 8050-8054.

3. Weber C, Lee VD, Chazin WJ, Huang B (1994) High level expression in Escherichia coli and characterization of the EF-hand calcium-binding protein caltractin. J Biol Chem 269: 15795-15802.

4. Veeraraghavan S, Fagan PA, Hu H, Lee V, Harper JF, et al. (2002) Structural independence of the two EF-hand domains of caltractin. J Biol Chem 277: 28564-28571.

5. Rhyner JA, Koller M, Durussel-Gerber I, Cox JA, Strehler EE (1992) Characterization of the human calmodulin-like protein expressed in Escherichia coli. Biochemistry 31: 12826-12832.

6. Linse S, Johansson C, Brodin P, Grundstrom T, Drakenberg T, et al. (1991) Electrostatic contributions to the binding of Ca2+ in calbindin D9k. Biochemistry 30: 154-162.

7. Dell'Angelica EC, Schleicher CH, Santome JA (1994) Primary structure and binding properties of calgranulin C, a novel S100-like calcium-binding protein from pig granulocytes. J Biol Chem 269: 28929-28936.

8. Lu G, Sehnke PC, Ferl RJ (1994) Phosphorylation and calcium binding properties of an Arabidopsis GF14 brain protein homolog. Plant Cell 6: 501-510.

9. Di Pietro SM, Santome JA (2002) Structural and biochemical characterization of calhepatin, an S100-like calcium-binding protein from the liver of lungfish (Lepidosiren paradoxa). Eur J Biochem 269: 3433-3441.

10. Gopal B, Swaminathan CP, Bhattacharya S, Bhattacharya A, Murthy MR, et al. (1997) Thermodynamics of metal ion binding and denaturation of a calcium binding protein from Entamoeba histolytica. Biochemistry 36: 10910-10916.

11. Rout AK, Padhan N, Barnwal RP, Bhattacharya A, Chary KV (2010) Calmodulin-like Protein from Entamoeba histolytica: Solution Structure and Calcium-Binding Properties of a Partially Folded Protein. Biochemistry.

12. Boguta G, Stepkowski D, Bierzynski A (1988) Theoretical estimation of the calcium-binding constants for proteins from the troponin C superfamily based on a secondary structure prediction method. I. Estimation procedure. J Theor Biol 135: 41-61.

13. Baudier J, Glasser N, Gerard D (1986) Ions binding to S100 proteins. I. Calcium- and zinc-binding properties of bovine brain S100 alpha alpha, S100a (alpha beta), and S100b (beta beta) protein: Zn2+ regulates Ca2+ binding on S100b protein. J Biol Chem 261: 8192-8203.

14. Baudier J, Glasser N, Haglid K, Gerard D (1984) Purification, characterization and ion binding properties of human brain S100b protein. Biochim Biophys Acta 790: 164-173.

15. Baudier J, Labourdette G, Gerard D (1985) Rat brain S100b protein: purification, characterization, and ion binding properties. A comparison with bovine S100b protein. J Neurochem 44: 76-84.

16. Benzonana G, Capony JP, Pechere JF (1972) The binding of calcium to muscular parvalbumins. Biochim Biophys Acta 278: 110-116.

17. Haiech J, Derancourt J, Pechere JF, Demaille JG (1979) Magnesium and calcium binding to parvalbumins: evidence for differences between parvalbumins and an explanation of their relaxing function. Biochemistry 18: 2752-2758.

18. Permyakov EA, Medvedkin VN, Kalinichenko LP, Burstein EA (1983) Comparative study of physiochemical properties of two pike parvalbumins by means of their intrinsic tyrosyl and phenylalanyl fluorescence. Arch Biochem Biophys 227: 9-20.

19. Williams TC, Corson DC, Oikawa K, McCubbin WD, Kay CM, et al. (1986) 1H NMR spectroscopic studies of calcium-binding proteins. 3. Solution conformations of rat apo-alpha-parvalbumin and metal-bound rat alpha-parvalbumin. Biochemistry 25: 1835-1846.

20. Burtnick LD, Kay CM (1977) The calcium-binding properties of bovine cardiac troponin C. FEBS Lett 75: 105-110.

21. Leavis PC, Kraft EL (1978) Calcium binding to cardiac troponin C. Arch Biochem Biophys 186: 411-415.

22. Barskaya NV, Gusev NB (1982) Biological activities of bovine cardiac-muscle troponin C C-terminal peptide (residues 84-161). Biochem J 207: 185-192.

23. Takagi T, Konishi K, Cox JA (1986) Amino acid sequence of two sarcoplasmic calcium-binding proteins from the protochordate Amphioxus. Biochemistry 25: 3585-3592.

24. Cox JA, Stein EA (1981) Characterization of a new sarcoplasmic calcium-binding protein with magnesium-induced cooperativity in the binding of calcium. Biochemistry 20: 5430-5436.

25. Bagshaw CR (1977) On the location of the divalent metal binding sites and the light chain subunits of vertebrate myosin. Biochemistry 16: 59-67.

26. Sugden EA, Nihei T (1969) The effects of calcium and magnesium ions on the adenosine triphosphatase and inosine triphosphatase activities of myosin A. Biochem J 113: 821-827.

27. Okamoto Y, Yagi K (1977) Inhibition by Mg2+ of the interaction of Ca2+ with spin-labeled g2 bound to myosin. J Biochem 82: 835-837.

28. Alexis MN, Gratzer WB (1978) Interaction of skeletal myosin light chains with calcium ions. Biochemistry 17: 2319-2325.

29. Morita F, Kondo S, Tomari K, Minowa O, Ikura M, et al. (1985) Calcium binding and conformation of regulatory light chains of smooth muscle myosin of scallop. J Biochem 97: 553-561.

30. Shimomura O, Johnson FH (1970) Calcium binding, quantum yield, and emitting molecule in aequorin bioluminescence. Nature 227: 1356-1357.

31. Allen DG, Blinks JR, Prendergast FG (1977) Aequorin luminescence: relation of light emission to calcium concentration--a calcium-independent component. Science 195: 996-998.

32. Klee CB, Crouch TH, Krinks MH (1979) Calcineurin: a calcium- and calmodulin-binding protein of the nervous system. Proc Natl Acad Sci U S A 76: 6270-6273.

33. Cox JA (1986) Isolation and characterization of a new Mr 18,000 protein with calcium vector properties in amphioxus muscle and identification of its endogenous target protein. J Biol Chem 261: 13173-13178.

34. Fraga H, Faria TQ, Pinto F, Almeida A, Brito RM, et al. (2010) FH8--a small EF-hand protein from Fasciola hepatica. FEBS J 277: 5072-5085.

35. Babini E, Bertini I, Borsi V, Calderone V, Hu X, et al. (2011) Structural characterization of human S100A16, a low-affinity calcium binder. J Biol Inorg Chem 16: 243-256.

36. Celic A, Petri ET, Demeler B, Ehrlich BE, Boggon TJ (2008) Domain mapping of the polycystin-2 C-terminal tail using de novo molecular modeling and biophysical analysis. J Biol Chem 283: 28305-28312.

37. Kanuru M, Samuel JJ, Balivada LM, Aradhyam GK (2009) Ion-binding properties of Calnuc, Ca2+ versus Mg2+--Calnuc adopts additional and unusual Ca2+-binding sites upon interaction with G-protein. FEBS J 276: 2529-2546.

38. Cox JA, Tirone F, Durussel I, Firanescu C, Blouquit Y, et al. (2005) Calcium and magnesium binding to human centrin 3 and interaction with target peptides. Biochemistry 44: 840-850.

39. Durussel I, Blouquit Y, Middendorp S, Craescu CT, Cox JA (2000) Cation- and peptide-binding properties of human centrin 2. FEBS Lett 472: 208-212.

40. Miron S, Durand D, Chilom C, Perez J, Craescu CT (2011) Binding of calcium, magnesium, and target peptides to Cdc31, the centrin of yeast Saccharomyces cerevisiae. Biochemistry 50: 6409-6422.

41. Yu L, Sun C, Mendoza R, Wang J, Matayoshi ED, et al. (2007) Solution structure and calcium-binding properties of EF-hands 3 and 4 of calsenilin. Protein Sci 16: 2502-2509.

**Supplementary Table S7** Calcium binding EF-hand proteins sequences in FASTA format at 60% sequence redundancy WITH EF hand Loop region RESIDUES LABELLED IN lower case letters. **(D1)** The sequences were taken from Uniprot (Keyword search) / PFAM (alignment) based and then cross validated using RCSB PDB database.

| >CABP1_HUMAN/200-227  DIEEIIRDVdlngdgrvdfeeFVRMMSR  >CABP1_HUMAN/163-191  ELRDAFREFdtngdgeistseLREAMRKL  >CABP1_HUMAN/86-114  ELREAFREFdkdkdgyincrdLGNCMRTM  >CALB1_RAT/190-218  EFNKAFELYdqdgngyideneLDALLKDL  >CALB1_RAT/102-130  EFMKTWRKYdtdhsgfieteeLKNFLKDL  >CALBP_ENTHI/1-29  MAEALFKEIdvngdgavsyeeVKAFVSKK  >CALL3_HUMAN/121-149  EVDEMIRAAdtdgdgqvnyeeFVRVLVSK  >CALL3_HUMAN/48-76  ELRDMMSEIdrdgngtvdfpeFLGMMARK  >CALL3_HUMAN/12-40  EFKEAFSLF dkdgdgcittreLGTVMRSL  >CALL3_HUMAN/85-113  EIREAFRVFdkdgngfvsaaeLRHVMTRL  >CALL5_HUMAN/118-146  ELDAMIREAdvdqdgrvnyeeFARMLAQE  >CALL5_HUMAN/82-110  DLQVAFRAFdqdgdghitvdeLRRAMAGL  >CALM2_SOYBN/85-113  ELKEAFRVFdkdqngfisaaeLRHVMTNL  >CALM2_SOYBN/121-149  EVDEMIREAdvdgdgqinyeeFVKVMMAK  >CALM_BOVIN/85-113  EIREAFRVFdkdgngyisaaeLRHVMTNL  >CALM_BOVIN/121-149  EVDEMIREAdidgdgqvnyeeFVQMMTAK  >CALM_PARTE/121-149  EVDEMIREAdidgdghinyeeFVRMMVSK  >CALM_PARTE/12-40  EFKEAFALFdkdgdgtittkeLGTVMRSL  >CALM_PARTE/85-113  ELIEAFKVFdrdgnglisaaeLRHVMTNL  >CALM_YEAST/12-40  EFKEAFALFdkdnngsissseLATVMRSL  >CALM_YEAST/85-113  ELLEAFKVFdkngdglisaaeLKHVLTSI  >CANB1_BOVIN/132-160  IVDKTIINAdkdgdgrisfeeFCAVVGGL  >CANB1_BOVIN/54-82  LVQRVIDIFdtdgngevdfkeFIEGVSQF  >CANB1_BOVIN/91-119  KLRFAFRIYdmdkdgyisngeLFQVLKMM  >CATR_CHLRE/29-57  EIREAFDLFdtdgsgtidakeLKVAMRAL  >CATR_CHLRE/102-130  EILKAFRLFdddnsgtitikdLRRVAKEL  >CATR_CHLRE/138-166  ELQEMIAEAdrnddneidedeFIRIMKKT  >CATR_CHLRE/65-93  EIKKMISEIdkdgsgtidfeeFLTMMTAK  >CAVP_BRALA/90-118  EILRAFKVFdangdgvidfdeFKFIMQKV  >CAVP_BRALA/127-155  EVEEAMKEAdedgngvidipeFMDLIKKS  >CBP_SACER/138-166  EAAEAFNQVdtngngelsldeLLTAVRDF  >CDC31_YEAST/133-161  ELRAMIEEFdldgdgeineneFIAICTDS  >CDC31_YEAST/24-52  EIYEAFSLFdmnndgfldyheLKVAMKAL  >CDPK1_ARATH/527-555  HLFAAFTYFdkdgsgyitpdeLQQACEEF  >CDPK1_ARATH/455-483  GLKEMFNMIdadksgqitfeeLKAGLKRV  >CDPK1_ARATH/561-589  RIEELMRDVdqdndgridyneFVAMMQKG  >CDPK_SOYBN/445-473  HIDDMIKEIdqdndgqidygeFAAMMRKG  >CDPK_SOYBN/411-439  NLVSAFSYFdkdgsgyitldeIQQACKDF  >CDPK_SOYBN/339-367  GLKELFKMIdtdnsgtitfdeLKDGLKRV  >CETN2_HUMAN/141-169  ELQEMIDEAdrdgdgevseqeFLRIMKKT  >CETN2_HUMAN/32-60  EIREAFDLFdadgtgtidvkeLKVAMRAL  >CHP1_HUMAN/114-142  KLHFAFRLYdldkdekisrdeLLQVLRMM  >CHP2_HUMAN/115-143  KLHYAFQLYdldrdgkisrheMLQVLRLM  >CLSS_HAEMA/50-78  ASAKLIKMAdknsdgkiskeeFLNANAEL  >CLSS_HAEMA/8-36  ELEAAFKKLdangdgyvtaleLQTFMVTL  >CSEN_HUMAN/166-194  KLKWAFNLYdinkdgyitkeeMLAIMKSI  >CSEN_HUMAN/214-242  HVERFFEKMdrnqdgvvtieeFLEACQKD  >CSEN_MOUSE/214-242  HVERFFQKMdrnqdgvvtideFLETCQKD  >GUC1A_CHICK/54-82  YVEQMFETFdfnkdgyidfmeYVAALSLV  >GUC1B_BOVIN/60-88  YVEAMFRAFdtngdntidfleYVAALNLV | >GUC1B_BOVIN/96-124  KLKWTFKIYdkdrngcidrqeLLDIVESI  >GUC1C_HUMAN/92-120  KLKWYFKLYdadgngsidkneLLDMFMAV  >KCIP1_HUMAN/137-165  KLRWTFNLYdinkdgyinkeeMMDIVKAI  >KCIP4_MOUSE/160-188  KLNWAFNLYdinkdgyitkeeMLDIMKAI  >MLR_AEQIR/20-48  EMKEAFSMIdvdrdgfvskedIKAISEQL  >MLR_PHYPO/6-34  QIQECFQIFdkdndgkvsieeLGSALRSL  >MLR_TODPA/17-45  ELKEAFTMIdqdrdgfigmedLKDMFSSL  >NCALD_BOVIN/148-176  RTEKIFRQMdtnrdgklsleeFIRGAKSD  >NCALD_BOVIN/64-92  FAEHVFRTFdangdgtidfreFIIALSVT  >NCALD_BOVIN/100-128  KLKWAFSMYdldgngyiskaeMLEIVQAI  >NCS1_HUMAN/64-92  FATFVFNVFdenkdgriefseFIQALSVT  >NCS1_HUMAN/100-128  KLRWAFKLYdldndgyitrneMLDIVDAI  >NCS1_HUMAN/148-176  RVDRIFAMMdknadgkltlqeFQEGSKAD  >NCS1_YEAST/100-128  KLSWAFELYdlnhdgyitfdeMLTIVASV  >NCS1_YEAST/148-176  RVKKIFKLMdknedgyitldeFREGSKVD  >NCS1_YEAST/64-92  FANHLFTVFdkdnngfihfeeFITVLSTT  >OBL_OBELO/116-142  --DAVFDIFdkdgsgtitldeWKAYGKIS  >ONCO_RAT/82-109  ETKSLMDAAdndgdgkigadeFQEMVHS-  >PDCD6_HUMAN/94-122  DWQNVFRTYdrdnsgmidkneLKQALSGF  >POLC3_CHEAL/47-75  EVRRMMAEIdtdgdgfisfdeFTDFARAN  >POLC3_CHEAL/12-40  DRERIFKRFdtngdgkissseLGDALKTL  >POLC4_BETVE/11-39  ERERIFKRFdangdgkisaaeLGEALKTL  >POLC4_BETVE/46-74  EVKHMMAEIdtdgdgfisfqeFTDFGRAN  >POLC7_PHLPR/4-32  DMERIFKRFdtngdgkislseLTDALRTL  >POLC7_PHLPR/39-67  EVQRMMAEIdtdgdgfidfneFISFCNAN  >PRVA_ESOLU/41-69  DVKKVFKAIdadasgfieeeeLKFVLKSF  >PRVA_HUMAN/43-71  DVKKVFHMLdkdksgfieedeLGFILKGF  >PRVA_TRISE/42-70  QVKEVFEILdkdqsgfieeeeLKGVLKGF  >PRVB_CYPCA/42-70  DVKKAFAIIdqdksgfieedeLKLFLQNF  >Q26068_PLAMG/20-48  EMKEAFTMIdqnrdgfidindLKEMFSSL  >Q39890_SOYBN/121-149  EVEQMIKEAdldgdgqvnyeeFVKMMMTV  >Q39890_SOYBN/48-76  ELQDMISEVdadgngtiefdeFLSLMAKK  >Q39890_SOYBN/12-40  DFKEAFGLFdkdgdgcitveeLATVIRSL  >Q39890_SOYBN/85-113  ELKEAFKVFdkdqngyisaseLRHVMINL  >Q7ZZB9_ONCMY/56-84  ELQEMIDEVdedgsgtvdfdeFLVMMVRC  >Q868D4_9HEMI/128-156  DLDAMIDEIdadgsgtvdfeeFMGVMTGG  >Q868D4_9HEMI/92-120  ELREAFRLYdkegngyistdvMREILAEL  >Q8WSQ4_PHYPO/56-84  AFNEMFNEAdatgngkiqfpeFLSMMGRR  >Q9XZV2_EUPOC/28-56  EIKEAFDLFdtnktgsidyheLKVAMRAL  >Q9XZV2_EUPOC/64-92  EILELMNEYdregngyigfddFLDIMTEK  >RECO_BOVIN/101-129  KLEWAFSLYdvdgngtiskneVLEIVTAI  >S100B_BOVIN/53-81  VVDKVMETLdsdgdgecdfqeFMAFVAMI  >S10A1_BOVIN/54-82  AVDKVMKELdengdgevdfqeYVVLVAAL  >S10AB_PIG/57-85  VLDRMMKKLdldsdgqldfqeFLNLIGGL  >TNNC1_CHICK/96-124  ELSDLFRMFdknadgyidleeLKIMLQAT  >TNNC2_CHICK/98-126  ELANCFRIFdknadgfidieeLGEILRAT  >TNNC2_CHICK/58-86  ELDAIIEEVdedgsgtidfeeFLVMMVRQ  >TNNC2_CHICK/134-162  DIEDLMKDSdknndgridfdeFLKMMEGV  >TNNC2_RABIT/95-123  ELAECFRIFdrnadgyidaeeLAEIFRAS  >TNNC2_RABIT/131-159  EIESLMKDGdknndgridfdeFLKMMEGV |
| --- | --- |

**Supplementary Table S8** The list of 12-mer Sequences from non-binding region of Calcium Binding EF-hand proteins with 60% sequence redundancy. The sequences are taken from Uniprot (Keyword search) / PFAM (alignment) based and then cross validated using RCSB PDB database. (D2)

| DIEEIIRDVFVR | FRVFLRHVMTNL | VFFKFIMQKVEV | MLQVLRLMASAK | DMFSSLRTEKIF |
| --- | --- | --- | --- | --- |
| MMSRLRDAFREF | EVDEMIREAFVQ | EEAMKEAFMDLI | LIKMAFLNANAE | RQMFIRGAKSDF |
| LREAMRKLELRE | MMTAKEVDEMIR | KKSEAAEAFNQV | LELEAAFKKLLQ | AEHVFRTFFIIA |
| AFREFLGNCMRT | EAFVRMMVSKEF | LLTAVRDFELRA | TFMVTLKLKWAF | LSVTKLKWAFSM |
| MEFNKAFELYDA | KEAFALFLGTVM | MIEEFFIAICTD | NLYMLAIMKSIH | YMLEIVQAIFAT |
| LLKDLEFMKTWR | RSLELIEAFKVF | SEIYEAFSLFLK | VERFFEKMFLEA | FVFNVFFIQALS |
| KYLKNFLKDLMA | LRHVMTNLEFKE | VAMKALHLFAAF | CQKDHVERFFQK | VTKLRWAFKLYM |
| EALFKEIVKAFV | AFALFLATVMRS | TYFLQQACEEFG | MFLETCQKDYVE | LDIVDAIRVDRI |
| SKKEVDEMIRAA | LELLEAFKVFLK | LKEMFNMILKAG | QMFETFYVAALS | FAMMFQEGSKAD |
| FVRVLVSKELRD | HVLTSIIVDKTI | LKRVRIEELMRD | LVYVEAMFRAFY | KLSWAFELYMLT |
| MMSEIFLGMMAR | INAFCAVVGGLL | VFVAMMQKGHID | VAALNLVKLKWT | IVASVRVKKIFK |
| KEFKEAFSLFLG | VQRVIDIFFIEG | DMIKEIFAAMMR | FKIYLLDIVESI | LMFREGSKVDFA |
| TVMRSLEIREAF | VSQFKLRFAFRI | KGNLVSAFSYFI | KLKWYFKLYLLD | NHLFTVFFITVL |
| RVFLRHVMTRLE | YLFQVLKMMEIR | QQACKDFGLKEL | MFMAVKLRWTFN | STTDAVFDIFWK |
| LDAMIREAFARM | EAFDLFLKVAMR | FKMILKDGLKRV | LYMMDIVKAIKL | AYGKISETKSLM |
| LAQEDLQVAFRA | ALEILKAFRLFL | ELQEMIDEAFLR | NWAFNLYMLDIM | DAAFQEMVHSDW |
| FLRRAMAGLELK | RRVAKELELQEM | IMKKTEIREAFD | KAIEMKEAFSMI | QNVFRTYLKQAL |
| EAFRVFLRHVMT | IAEAFIRIMKKT | LFLKVAMRALKL | IKAISEQLQIQE | SGFEVRRMMAEI |
| NLEVDEMIREAF | EIKKMISEIFLT | HFAFRLYLLQVL | CFQIFLGSALRS | FTDFARANDRER |
| VKVMMAKEIREA | MMTAKEILRAFK | RMMKLHYAFQLY | LELKEAFTMILK | IFKRFLGDALKT |
| LERERIFKRFLG | GFILKGFQVKEV | AFGLFLATVIRS | RREIKEAFDLFL | FLNLIGGLELSD |
| EALKTLEVKHMM | FEILLKGVLKGF | LELKEAFKVFLR | KVAMRALEILEL | LFRMFLKIMLQA |
| AEIFTDFGRAND | DVKKAFAIILKL | HVMINLELQEMI | MNEYFLDIMTEK | TELANCFRIFLG |
| MERIFKRFLTDA | FLQNFEMKEAFT | DEVFLVMMVRCD | KLEWAFSLYVLE | EILRATELDAII |
| LRTLEVQRMMAE | MILKEMFSSLEV | LDAMIDEIFMGV | IVTAIVVDKVME | EEVFLVMMVRQD |
| IFISFCNANDVK | EQMIKEAFVKMM | MTGGELREAFRL | TLFMAFVAMIAV | IEDLMKDSFLKM |
| KVFKAILKFVLK | MTVELQDMISEV | YMREILAELAFN | DKVMKELYVVLV | MEGVELAECFRI |
| SFDVKKVFHMLL | FLSLMAKKDFKE | EMFNEAFLSMMG | AALVLDRMMKKL | FLAEIFRASEIE |

**Supplementary Table S9** The training data used for estimation of binding affinity were taken from RCSB based on PSSM scores obtained from the EF hand loop region. The positive dataset (**D3**) consisted of one hundred forty four 12-mer sequences and there were 124 sequences in the negative dataset (**D4**).

| **HIGH BINDERS (D3)** | **PSSM SCORE** |  | **LOW BINDERS (D4)** | **PSSM SCORE** |
| --- | --- | --- | --- | --- |
| DRDGDGYISADE | 6.77 |  | DADNSGDISLRE | 4.89 |
| DKNGDGYIDLEE | 6.67 |  | DTDGNGFLDSSE | 4.88 |
| DTDGDGYISYQE | 6.66 |  | DANNDGRITIDE | 4.87 |
| DKDGNGYITVEE | 6.55 |  | DSDGNGFLDKSE | 4.87 |
| DKDGSGYITVDE | 6.56 |  | DQNKSGFIEVEE | 4.85 |
| DKDGSGYITLDE | 6.54 |  | DADSNGNIEFKE | 4.84 |
| DEDGDGYISARE | 6.48 |  | DRDRDGEVNVEE | 4.84 |
| DKDGSGYITIDE | 6.48 |  | DENGDGEVDFQE | 4.84 |
| DKDGSGYITIDE | 6.48 |  | DFNKDGHIDINE | 4.82 |
| DKDGSGYITPDE | 6.47 |  | DLNGDGKVDLNE | 4.81 |
| DIDGDGYISNGE | 6.40 |  | DEDSNGSIDHTE | 4.80 |
| DVDGDGYITRSE | 6.38 |  | DDNQDGKIDIRE | 4.79 |
| DTNGDGYIDRDE | 6.38 |  | DADHSGTINSYE | 4.79 |
| DKDGDGKIDVDE | 6.36 |  | DENKDGAIEFHE | 4.78 |
| DVDGDGEIDYEE | 6.36 |  | DKDKDGRVNALE | 4.77 |
| DLDGNGYISREE | 6.31 |  | DKDKNGFLTREE | 4.76 |
| DQDGNGYIDENE | 6.29 |  | DLDNSGKLDVDE | 4.75 |
| DQDGSGYITRDE | 6.28 |  | DEDGGGDVDFQE | 4.74 |
| DKDGNGYITAQE | 6.27 |  | DADHSGKLSFEE | 4.72 |
| DVDNDGYITREE | 6.25 |  | DKNHDSQIDYEE | 4.72 |
| DTDGNGYISFNE | 6.23 |  | DNDGSGKLGLKE | 4.72 |
| DKDGDGRISFEE | 6.22 |  | DKNCDGRLDFDE | 4.70 |
| DKDGDGRISFEE | 6.22 |  | DLDGNGQVEFPE | 4.70 |
| DLDQDGYISQEE | 6.22 |  | DRDRDGEVNMDE | 4.68 |
| DKDGNGYIEGTE | 6.21 |  | DKDKNGELDENE | 4.67 |
| DKDGDGKIGVEE | 6.18 |  | DEDGQGFIPEDY | 4.66 |
| DKNGDGYITVNE | 6.19 |  | DKNSDGHVDEDE | 4.65 |
| DKDRSGYIEEEE | 6.17 |  | DMRNDGAIDFGE | 4.64 |
| DKNADGYIDLDE | 6.16 |  | DANKDGFVEFDE | 4.63 |
| DKNADGYIDGEE | 6.15 |  | DASHDGGIDVTE | 4.62 |
| DKDGDGKIGVDE | 6.13 |  | DEDKSGRLEFEE | 4.62 |
| DKDNSGYITKEE | 6.13 |  | DENGDGSVNFKE | 4.60 |
| DEDGDGKISFEE | 6.10 |  | DCDGNGELSNKE | 4.59 |
| DKDGNGYILPQE | 6.10 |  | DTEGDGVLTVEE | 4.59 |
| DKDASGYITIEE | 6.07 |  | DENGDGQLSLNE | 4.58 |
| DQDGDGRIDYNE | 6.06 |  | DADNSGDVDFQE | 4.57 |
| DKDGDGKIGIDE | 6.05 |  | DGDNDGELEENE | 4.56 |
| DKDGDGKISFQE | 6.05 |  | DREGQGFISGAE | 4.55 |
| DQDKSGYIEEEE | 6.05 |  | DKDNSGQVSMKE | 4.52 |
| DADKNGYIDFKE | 6.04 |  | DKDNDGKVSVED | 4.51 |
| DVDGDGVIDYSE | 6.05 |  | DKNGTGSVTFDE | 4.50 |
| DVDGNGTIDYYE | 6.05 |  | DQNRDGFIDKED | 4.48 |
| DLNGDGYIQREE | 6.04 |  | DEDGDHQVDFKE | 4.47 |
| DTDGDGFIDFNE | 6.03 |  | DRDHSGTLGPEE | 4.47 |
| DKDGDGCITVDE | 6.02 |  | DKNSDGTVTWDE | 4.46 |
| DKDGDGMIGVDE | 6.02 |  | DEKKNGVIEFEE | 4.45 |
| DQDGDGFITVEE | 6.02 |  | DKNKDRKIDFSE | 4.44 |
| DQDKSGYIEEDE | 6.01 |  | DKNMDGRLSIDE | 4.44 |
| DRDKSGYIEEDE | 6.00 |  | DANSDGTLDFKE | 4.44 |
| DTDGDGKIGVEE | 6.00 |  | DADKDGIIGKND | 4.42 |
| DTDGDGKIGVEE | 6.00 |  | DINNSGDIDHYE | 4.41 |
| DADGDGYVSLQE | 5.99 |  | DGDGNSYITTDE | 4.41 |
| DMDGDGSIDYLE | 5.98 |  | DIDNDGGLNNQE | 4.40 |
| DKDGSGAIDFDE | 5.98 |  | DTDGTQSIDPKE | 4.39 |
| DTDNSGYIEADE | 5.98 |  | DTNADGVVDFQE | 4.39 |
| DKDGDGKITAAE | 5.96 |  | DFDKDGAITRKD | 4.39 |
| DKDSSGYITIDE | 5.97 |  | DADKSGTMSTYE | 4.38 |
| DRNMDGYIDAEE | 5.97 |  | DINNDGELTLEE | 4.36 |
| DKDASGYISSAE | 5.94 |  | DLDKNGKISPDD | 4.36 |
| DLDGDGTIDFPE | 5.93 |  | DKNADGKLTLQE | 4.35 |
| DMDNDGYISNGE | 5.93 |  | DSDKSGQLEEKE | 4.35 |
| DLDGDGFIDFRE | 5.92 |  | DANSDGVVTFDE | 4.34 |
| DTDGDGKITSEE | 5.93 |  | DANNDGKLSEKE | 4.33 |
| DYDRDGTVSLEE | 5.93 |  | DKNKDDQITLDE | 4.32 |
| DKNEDGYITLDE | 5.92 |  | DEDEDGLISRGD | 4.31 |
| DADGNGLIDYDE | 5.90 |  | DINSDGQLDFQE | 4.29 |
| DGDQSGYIEVEE | 5.90 |  | DKDNNELIDKQE | 4.28 |
| DIDGDGFITPEE | 5.91 |  | DKNSDQEIDFKE | 4.27 |
| DEDGSGTIDFEE | 5.89 |  | DCNNDGQVNYEE | 4.26 |
| DEDGSGTIDFEE | 5.89 |  | DKDGSRPVDFSE | 4.25 |
| DKDGDGKITTKE | 5.90 |  | DRDGSRSLDADE | 4.25 |
| DTDGDGFISFQE | 5.90 |  | DKNGNGTISSLD | 4.23 |
| DVDGNGVIDYDE | 5.89 |  | DQDGDKQLSLPE | 4.22 |
| DADGDGHITFDE | 5.88 |  | DIDHNKKIDFTE | 4.21 |
| DYDNDGIVSFDE | 5.87 |  | DLNSDGEVDMAE | 4.20 |
| DLDGSGTIDFEE | 5.87 |  | DLNKDNKISWEE | 4.19 |
| DTDGDGKISAAE | 5.87 |  | DQNRDGFIDIND | 4.19 |
| DNDGDGKIGADE | 5.85 |  | DINSNGQINLNE | 4.16 |
| DADGDGTISFSE | 5.85 |  | DEDDSGFITFAN | 4.16 |
| DKNGDGFIDKDE | 5.85 |  | DPNATGNINKDE | 4.15 |
| DRDGDGEINEEE | 5.85 |  | DQRGNHQIDFDE | 4.14 |
| DVDGDGHISQEE | 5.85 |  | DINRSGFVDFTE | 4.12 |
| DVDNDGYLDYGE | 5.85 |  | DVNCDGRMQFDE | 4.12 |
| DKDNDGRIDYSE | 5.82 |  | DGNHDGGLNREE | 4.11 |
| DADGNGEIDFEE | 5.81 |  | DVDRSGTMNSYE | 4.11 |
| DNDNSGYITMEE | 5.81 |  | DLNKDGVLSRSE | 4.10 |
| DFNKDGYIDFME | 5.81 |  | DPNRDGHVSLQE | 4.07 |
| DKDQNGYISPSE | 5.80 |  | DVDRDGFVNKDD | 4.07 |
| DRDGDGFISPAE | 5.80 |  | DMNNDGRMDQLE | 4.05 |
| DSDGDGAITEDE | 5.80 |  | DINTDGAVNFQE | 4.04 |
| DTDGDGVINYEE | 5.81 |  | DVNSDNAINFEE | 4.02 |
| DTDKDGKISYEE | 5.80 |  | DGNGDGFVCFDD | 4.01 |
| DVDGDGQINYEE | 5.81 |  | DINSDNAINFEE | 4.01 |
| DKDEDGKISFDE | 5.79 |  | DTSGSGMIDLND | 3.99 |
| DKDGSGTIDTKE | 5.78 |  | DKNRTGRLSPEE | 3.98 |
| DKDGNGTISKDE | 5.77 |  | DLNDDGRVQFNE | 3.98 |
| DADGDGMIGIDE | 5.76 |  | DCDRDGLVTYDD | 3.97 |
| DLNHDGYITFDE | 5.75 |  | DKDNDRFVTKCE | 3.97 |
| DKNGDGKISVDE | 5.74 |  | DSNKNGTLDPSE | 3.95 |
| DKDGDGAITRSE | 5.73 |  | DFDDDGTLNRED | 3.94 |
| DFDGDGMINYEE | 5.72 |  | DLNQDGVLTSQE | 3.93 |
| DVDKDGYLDVNE | 5.72 |  | DADKDGVVTVND | 3.92 |
| DEDGSGKIEFEE | 5.70 |  | DKDGNNTMNIKE | 3.88 |
| DKNKSGYIEIEE | 5.70 |  | DTNRSGTITYEQ | 3.87 |
| DLDKDGKISFEE | 5.70 |  | DANGDNKLDQLE | 3.85 |
| DFDKNGYIEYSE | 5.70 |  | DKNKDDKLTFDE | 3.85 |
| DKNGDGLISVEE | 5.68 |  | EQDHDGRVDFFE | 3.83 |
| DKDGNGFISAAE | 5.68 |  | DQDKSDFVEEDE | 3.82 |
| DIDGNGKISVEE | 5.66 |  | DQNRDGIICKAD | 3.81 |
| DKDGSGHITKEE | 5.67 |  | DSNCSGTLSKKE | 3.81 |
| DVDGNGSIDYVE | 5.65 |  | DLNKNGQVELNE | 3.78 |
| DANGDGVIDFDE | 5.65 |  | DRDDDGVVSRGD | 3.76 |
| DEDGSGEIEFEE | 5.63 |  | DRNASDTISCDE | 3.75 |
| DEDGSGQIEFEE | 5.62 |  | DKNNDDLLSVDE | 3.73 |
| DEDGSGTIDFNE | 5.62 |  | DRNRSGTLEPHE | 3.73 |
| DKDQDGLISKDE | 5.60 |  | DKNNDAQLTLEE | 3.69 |
| DQDNDGRIDYGE | 5.61 |  | DKNKDNKMSFKE | 3.68 |
| DSDNDGRIDYSE | 5.60 |  | DVNHDGVVSFDD | 3.68 |
| DIDGDGQITSKE | 5.58 |  | DKNNDEAVDKKE | 3.66 |
| DKDCDGNIDFQE | 5.58 |  | DIDNNGFLDQND | 3.65 |
| DCDGDGKINRKE | 5.56 |  | DTNSDGKVEEDD | 3.63 |
| DKDGNGTISIKE | 5.56 |  | DTNQDNQLSFEE | 3.62 |
| DANGDGYFTLEE | 5.56 |  | DCNKDNEVDFQE | 3.55 |
| DVDGNGKIDFGE | 5.56 |  | DANQDEQVDFQE | 3.52 |
| DIDGNGTIDEKE | 5.55 |  |  | |
| DKNGDGRITKEE | 5.55 |  |  | |
| DADGSGYLEGKE | 5.53 |  |  | |
| DTDGDGKIAPSE | 5.53 |  |  | |
| DKDNSGYLTVDE | 5.53 |  |  | |
| DKDKDGFIEKME | 5.52 |  |  | |
| DADEKGYIEEKE | 5.50 |  |  | |
| DLDNDGKIDFSE | 5.51 |  |  | |
| DKDGDGCVTVEE | 5.49 |  |  | |
| DIDGSGSIDASE | 5.48 |  |  | |
| DHDRDGFISQEE | 5.47 |  |  | |
| DKDGNGLITAAE | 5.47 |  |  | |
| DLDQDGRISFDE | 5.45 |  |  | |
| DEDGSGTIDPVE | 5.45 |  |  | |
| DADGNGSIDKNE | 5.44 |  |  | |
| DKDKNGKISPEE | 5.42 |  |  | |
| DHDHDGYISQED | 5.42 |  |  | |
| DTNGDGSIDFRE | 5.41 |  |  | |
| DRDNDGYLSDTE | 5.39 |  |  | |
| DANGDGKISAAE | 5.38 |  |  | |

**Supplementary Table S10** The redundant set of PDB ids of EF hand containing calcium binding proteins. The sequences taken from RCSB were further processed using CD-HIT and the list if the sequences with different threshold are listed in supplementary sheet5.

| 1A03; 1A29; 1A2X; 1A75; 1AHR; 1AJ4; 1AJ5; 1AK8; 1ALV; 1ALW; 1AP4; 1AUI; 1AVS; 1B1G; 1B4C; 1B7T; 1B8C; 1B8L; 1B8R; 1B9A; 1BJF; 1BLQ; 1BMO; 1BOC; 1BOD; 1BU3; 1C07; 1C7V; 1C7W; 1CB1; 1CDL; 1CDM; 1CDN; 1CDP; 1CFC; 1CFD; 1CFF; 1CFP; 1CKK; 1CLB; 1CLL; 1CLM; 1CM1; 1CM4; 1CMF; 1CMG; 1CNP; 1CTA; 1CTD; 1CTR; 1DEG; 1DF0; 1DFK; 1DFL; 1DGU; 1DGV; 1DJG; 1DJH; 1DJI; 1DJW; 1DJX; 1DJY; 1DJZ; 1DMO; 1DT7; 1DTL; 1DVI; 1EH2; 1EJ3; 1EL4; 1EXR; 1F4O; 1F4Q; 1F54; 1F55; 1F70; 1F71; 1F8H; 1FF1; 1FI5; 1FI6; 1FPW; 1FW4; 1G33; 1G4Y; 1G8I; 1GGW; 1GGZ; 1GJY; 1H4B; 1HQV; 1HT9; 1I84; 1IG5; 1IGV; 1IH0; 1IJ5; 1IJ6; 1IKU; 1IQ3; 1IQ5; 1IRJ; 1IWQ; 1J1D; 1J1E; 1J7O; 1J7P; 1JBA; 1JC2; 1JF0; 1JF2; 1JFJ; 1JFK; 1JSA; 1JUO; 1JWD; 1K2H; 1K8U; 1K90; 1K93; 1K94; 1K95; 1K96; 1K9K; 1K9P; 1K9U; 1KCY; 1KFU; 1KFX; 1KK7; 1KK8; 1KQM; 1KQV; 1KSM; 1KWO; 1L2O; 1L7Z; 1LA0; 1LA3; 1LIN; 1LKJ; 1LVC; 1LXF; 1M31; 1M39; 1M63; 1M8Q; 1MF8; 1MHO; 1MQ1; 1MR8; 1MUX; 1MVW; 1MWN; 1MXE; 1MXL; 1N0Y; 1N65; 1NCX; 1NCY; 1NCZ; 1NIW; 1NP8; 1NPQ; 1NSH; 1NUB; 1NWD; 1NX0; 1NX1; 1NX2; 1NX3; 1NYA; 1O18; 1O19; 1O1A; 1O1B; 1O1C; 1O1D; 1O1E; 1O1F; 1O1G; 1OHZ; 1OMD; 1OMR; 1OMV; 1OOJ; 1OQP; 1OSA; 1OZS; 1PAL; 1PK0; 1PON; 1PRW; 1PSB; 1PSR; 1PVA; 1PVB; 1Q80; 1QIV; 1QIW; 1QLK; 1QLS; 1QV0; 1QV1; 1QVI; 1QX2; 1QX5; 1QX7; 1QXP; 1REC; 1RFJ; 1RJV; 1RK9; 1RRO; 1RTP; 1RWY; 1S1E; 1S26; 1S36; 1S3P; 1S5G; 1S6C; 1S6I; 1S6J; 1SBJ; 1SCM; 1SCV; 1SK6; 1SKT; 1SL7; 1SL8; 1SL9; 1SMG; 1SNL; 1SPY; 1SR6; 1SRA; 1SW8; 1SY9; 1SYM; 1TCF; 1TCO; 1TIZ; 1TN4; 1TNP; 1TNQ; 1TNW; 1TNX; 1TOP; 1TRF; 1TTX; 1U5I; 1UHH; 1UHI; 1UHJ; 1UHK; 1UP5; 1UWO; 1WDC; 1WRK; 1WRL; 1WRZ; 1X02; 1XA5; 1XFU; 1XFV; 1XFW; 1XFX; 1XFY; 1XFZ; 1XK4; 1XO5; 1XVJ; 1XYD; 1Y0V; 1Y1A; 1Y6W; 1YR5; 1YRT; 1YRU; 1YTZ; 1YV0; 1YX7; 1YX8; 1ZAC; 1ZFS; 1ZMZ; 1ZOT; 1ZUZ; 2A4J; 2AAO; 2AMI; 2B1U; 2B59; 2BBM; 2BBN; 2BCA; 2BCB; 2BCX; 2BE4; 2BE6; 2BEC; 2BKH; 2BKI; 2BL0; 2CCL; 2CNP; 2COL; 2CT9; 2CTN; 2D8N; 2DFS; 2DOQ; 2E6W; 2F2O; 2F2P; 2F33; 2F3Y; 2F3Z; 2F8P; 2FOT; 2G9B; 2GGM; 2GGZ; 2GV5; 2H61; 2HET; 2HF5; 2HPS; 2HQ8; 2HQW; 2I08; 2I18; 2I2R; 2I94; 2ISD; 2IX7; 2JC2; 2JPT; 2JQ6; 2JT0; 2JT3; 2JT8; 2JTT; 2JTZ; 2JU0; 2JUL; 2JWW; 2JXC; 2JXL; 2JZI; 2K0E; 2K0F; 2K0J; 2K2F; 2K2I; 2K3S; 2K61; 2K7B; 2K7C; 2K7D; 2K7O; 2KAX; 2KAY; 2KBM; 2KDH; 2KDU; 2KFF; 2KFG; 2KFH; 2KFX; 2KGB; 2KGR; 2KHN; 2KNE; 2KQY; 2KRD; 2KSP; 2KUG; 2KUH; 2KXW; 2KYC; 2KYF; 2KZ2; 2L0P; 2L1R; 2L2E; 2L4H; 2L4I; 2L50; 2L51; 2L53; 2L7L; 2L98; 2LAN; 2LAP; 2LCP; 2LGF; 2LHH; 2LHI; 2LHL; 2LL6; 2LL7; 2LLO; 2LLQ; 2LLS; 2LLT; 2LLU; 2LM5; 2LMT; 2LMU; 2LMV; 2LNK; 2LP2; 2LP3; 2LQC; 2LQP; 2LUC; 2LUX; 2LV6; 2LV7; 2LVI; 2LVJ; 2LVK; 2LVV; 2M3S; 2M55; 2M7K; 2M7M; 2M7N; 2MA2; 2MAZ; 2MYS; 2NLN; 2NXQ; 2NZ0; 2O5G; 2O60; 2OBH; 2OPO; 2P6B; 2PAL; 2PAS; 2PMY; 2PQ3; 2PRU; 2PSR; 2PVB; 2Q4U; 2Q91; 2QPT; 2R28; 2R2I; 2RGI; 2RO9; 2RRT; 2SAS; 2SCP; 2TN4; 2V01; 2V02; 2V53; 2VAS; 2VAY; 2VB6; 2VN5; 2VN6; 2VRG; 2W49; 2W490; 2W4A; 2W4G; 2W4H; 2W4T; 2W4U; 2W4U0; 2W4V; 2W4W; 2W73; 2WEL; 2WND; 2WOR; 2WOS; 2X0G; 2X51; 2Y4V; 2YGG; 2ZN8; 2ZN9; 2ZND; 2ZNE; 2ZRS; 2ZRT; 3A4U; 3A8R; 3AAJ; 3AAK; 3B32; 3BOW; 3BXK; 3BXL; 3BYA; 3C1V; 3CGA; 3CLN; 3CR2; 3CR4; 3CR5; 3CS1; 3CTN; 3CZT; 3D0Y; 3D10; 3DD4; 3DF0; 3DVE; 3DVJ; 3DVK; 3DVM; 3E3R; 3EK4; 3EK7; 3EK8; 3EKH; 3EVR; 3EVU; 3EVV; 3EWT; 3EWV; 3F45; 3FS7; 3FWB; 3FWC; 3G43; 3GK1; 3GK2; 3GK4; 3GN4; 3GOF; 3GP2; 3H4S; 3HCM; 3HR4; 3I5F; 3I5G; 3I5H; 3I5I; 3ICB; 3IF7; 3IFK; 3IQO; 3IQQ; 3J04; 3J41; 3JTD; 3JVT; 3K21; 3KCP; 3KF9; 3KO0; 3L9I; 3LCP; 3LI6; 3LK0; 3LK1; 3LL8; 3LLE; 3M0W; 3NXA; 3O77; 3O78; 3OX5; 3OX6; 3OXQ; 3PAL; 3PAT; 3PM8; 3PSR; 3PX1; 3QJK; 3QRX; 3RLZ; 3RM1; 3RV5; 3SG2; 3SG3; 3SG4; 3SG5; 3SG6; 3SG7; 3SJQ; 3SUI; 3UCT; 3UCW; 3UCY; 3ULG; 3WFN; 3ZWH; 4ANJ; 4AQI; 4AQJ; 4AQR; 4CLN; 4CPV; 4DBP; 4DBQ; 4DCK; 4DIR; 4DJC; 4DS7; 4DUQ; 4E50; 4E53; 4EHQ; 4ETO; 4F0Z; 4FL4; 4FQO; 4G27; 4G28; 4GGF; 4GOW; 4HEX; 4HSZ; 4I2Y; 4I5J; 4I5K; 4I5L; 4I5N; 4ICB; 4IL1; 4J9Y; 4J9Z; 4L9M; 4PAL; 4TNC; 5CPV; 5PAL; 5TNC |
| --- |

**Supplementary Table S11** The sequence wise classification of data obtained from PROSITE and RCSB. The data was further processed by using CD-HIT at 90%, 70%, 60%, 50% sequence redundancy cutoff for classification of EF-hand loop Ca^2+^-binding and non-binding region.

| **ALL SEQ** | **SEQ WITH 90% IDENTITY** | **SEQ WITH 60% IDENTITY** | **SEQ WITH 50% IDENTITY** |
| --- | --- | --- | --- |
| >CABP1_HUMAN/200-227 | >CABP1_HUMAN/200-227 | >CABP1_HUMAN/200-227 | >CABP1_HUMAN/163-191 |
| DIEEIIRDVDLNGDGRVDFEEFVRMM-SR | DIEEIIRDVDLNGDGRVDFEEFVRMM-SR | DIEEIIRDVDLNGDGRVDFEEFVRMM-SR | ELRDAFREFDTNGDGEISTSELREAMRKL |
| >CABP1_HUMAN/163-191 | >CABP1_HUMAN/163-191 | >CABP1_HUMAN/163-191 | >CALB1_RAT/15-43 |
| ELRDAFREFDTNGDGEISTSELREAMRKL | ELRDAFREFDTNGDGEISTSELREAMRKL | ELRDAFREFDTNGDGEISTSELREAMRKL | QFFEIWLHFDADGSGYLEGKELQNLIQEL |
| >CABP1_HUMAN/86-114 | >CABP1_HUMAN/86-114 | >CABP1_HUMAN/86-114 | >CALB1_RAT/190-218 |
| ELREAFREFDKDKDGYINCRDLGNCMRTM | ELREAFREFDKDKDGYINCRDLGNCMRTM | ELREAFREFDKDKDGYINCRDLGNCMRTM | EFNKAFELYDQDGNGYIDENELDALLKDL |
| >CALB1_RAT/15-43 | >CALB1_RAT/15-43 | >CALB1_RAT/15-43 | >CALB1_RAT/102-130 |
| QFFEIWLHFDADGSGYLEGKELQNLIQEL | QFFEIWLHFDADGSGYLEGKELQNLIQEL | QFFEIWLHFDADGSGYLEGKELQNLIQEL | EFMKTWRKYDTDHSGFIETEELKNFLKDL |
| >CALB1_RAT/190-218 | >CALB1_RAT/190-218 | >CALB1_RAT/190-218 | >CALB1_RAT/146-174 |
| EFNKAFELYDQDGNGYIDENELDALLKDL | EFNKAFELYDQDGNGYIDENELDALLKDL | EFNKAFELYDQDGNGYIDENELDALLKDL | YTDLMLKLFDSNNDGKLELTEMARLLPVQ |
| >CALB1_RAT/102-130 | >CALB1_RAT/102-130 | >CALB1_RAT/102-130 | >CALBP_ENTHI/37-65 |
| EFMKTWRKYDTDHSGFIETEELKNFLKDL | EFMKTWRKYDTDHSGFIETEELKNFLKDL | EFMKTWRKYDTDHSGFIETEELKNFLKDL | LLQLIFKSIDADGNGEIDQNEFAKFYGSI |
| >CALB1_RAT/146-174 | >CALB1_RAT/146-174 | >CALB1_RAT/146-174 | >CALBP_ENTHI/108-134 |
| YTDLMLKLFDSNNDGKLELTEMARLLPVQ | YTDLMLKLFDSNNDGKLELTEMARLLPVQ | YTDLMLKLFDSNNDGKLELTEMARLLPVQ | KVAEQVMKADANGDGYITLEEFLEF..SL |
| >CALBP_ENTHI/37-65 | >CALBP_ENTHI/37-65 | >CALBP_ENTHI/37-65 | >CALBP_ENTHI/76-104 |
| LLQLIFKSIDADGNGEIDQNEFAKFYGSI | LLQLIFKSIDADGNGEIDQNEFAKFYGSI | LLQLIFKSIDADGNGEIDQNEFAKFYGSI | GLKVLYKLMDVDGDGKLTKEEVTSFFKKH |
| >CALBP_ENTHI/37-65 | >CALBP_ENTHI/108-134 | >CALBP_ENTHI/108-134 | >CALBP_ENTHI/1-29 |
| LLQLIFKSIDADGNGEIDQNEFAKFYGSI | KVAEQVMKADANGDGYITLEEFLEF..SL | KVAEQVMKADANGDGYITLEEFLEF..SL | MAEALFKEIDVNGDGAVSYEEVKAFVSKK |
| >CALBP_ENTHI/108-134 | >CALBP_ENTHI/76-104 | >CALBP_ENTHI/76-104 | >CALL3_HUMAN/121-149 |
| KVAEQVMKADANGDGYITLEEFLEF..SL | GLKVLYKLMDVDGDGKLTKEEVTSFFKKH | GLKVLYKLMDVDGDGKLTKEEVTSFFKKH | EVDEMIRAADTDGDGQVNYEEFVRVLVSK |
| >CALBP_ENTHI/76-104 | >CALBP_ENTHI/1-29 | >CALBP_ENTHI/1-29 | >CALL3_HUMAN/48-76 |
| GLKVLYKLMDVDGDGKLTKEEVTSFFKKH | MAEALFKEIDVNGDGAVSYEEVKAFVSKK | MAEALFKEIDVNGDGAVSYEEVKAFVSKK | ELRDMMSEIDRDGNGTVDFPEFLGMMARK |
| >CALBP_ENTHI/1-29 | >CALL3_HUMAN/121-149 | >CALL3_HUMAN/121-149 | >CALL3_HUMAN/12-40 |
| MAEALFKEIDVNGDGAVSYEEVKAFVSKK | EVDEMIRAADTDGDGQVNYEEFVRVLVSK | EVDEMIRAADTDGDGQVNYEEFVRVLVSK | EFKEAFSLFDKDGDGCITTRELGTVMRSL |
| >CALBP_ENTHI/108-132 | >CALL3_HUMAN/48-76 | >CALL3_HUMAN/48-76 | >CALL5_HUMAN/12-40 |
| KVAEQVMKADANGDGYITLEEFLEF---- | ELRDMMSEIDRDGNGTVDFPEFLGMMARK | ELRDMMSEIDRDGNGTVDFPEFLGMMARK | QYKKAFSAVDTDGNGTINAQELGAALKAT |
| >CALL3_HUMAN/121-149 | >CALL3_HUMAN/12-40 | >CALL3_HUMAN/12-40 | >CALL5_HUMAN/48-76 |
| EVDEMIRAADTDGDGQVNYEEFVRVLVSK | EFKEAFSLFDKDGDGCITTRELGTVMRSL | EFKEAFSLFDKDGDGCITTRELGTVMRSL | QLRKLISEVDSDGDGEISFQEFLTAARKA |
| >CALL3_HUMAN/48-76 | >CALL3_HUMAN/85-113 | >CALL3_HUMAN/85-113 | >CALM_PARTE/85-113 |
| ELRDMMSEIDRDGNGTVDFPEFLGMMARK | EIREAFRVFDKDGNGFVSAAELRHVMTRL | EIREAFRVFDKDGNGFVSAAELRHVMTRL | ELIEAFKVFDRDGNGLISAAELRHVMTNL |
| >CALL3_HUMAN/12-40 | >CALL5_HUMAN/12-40 | >CALL5_HUMAN/12-40 | >CALM_YEAST/121-147 |
| EFKEAFSLFDKDGDGCITTRELGTVMRSL | QYKKAFSAVDTDGNGTINAQELGAALKAT | QYKKAFSAVDTDGNGTINAQELGAALKAT | EVDDMLREV-SDGSGEINIQQFAALLSK- |
| >CALL3_HUMAN/85-113 | >CALL5_HUMAN/48-76 | >CALL5_HUMAN/48-76 | >CANB1_BOVIN/132-160 |
| EIREAFRVFDKDGNGFVSAAELRHVMTRL | QLRKLISEVDSDGDGEISFQEFLTAARKA | QLRKLISEVDSDGDGEISFQEFLTAARKA | IVDKTIINADKDGDGRISFEEFCAVVGGL |
| >CALL5_HUMAN/12-40 | >CALL5_HUMAN/118-146 | >CALL5_HUMAN/82-110 | >CANB1_BOVIN/54-82 |
| QYKKAFSAVDTDGNGTINAQELGAALKAT | ELDAMIREADVDQDGRVNYEEFARMLAQE | DLQVAFRAFDQDGDGHITVDELRRAMAGL | LVQRVIDIFDTDGNGEVDFKEFIEGVSQF |
| >CALL5_HUMAN/48-76 | >CALL5_HUMAN/82-110 | >CALM_YEAST/48-76 | >CANB1_BOVIN/91-119 |
| QLRKLISEVDSDGDGEISFQEFLTAARKA | DLQVAFRAFDQDGDGHITVDELRRAMAGL | EVNDLMNEIDVDGNHQIEFSEFLALMSRQ | KLRFAFRIYDMDKDGYISNGELFQVLKMM |
| >CALL5_HUMAN/118-146 | >CALM2_SOYBN/48-76 | >CALM_YEAST/121-147 | >CANB1_BOVIN/22-50 |
| ELDAMIREADVDQDGRVNYEEFARMLAQE | ELQDMINEVDADGNGTIDFPEFLNLMARK | EVDDMLREV-SDGSGEINIQQFAALLSK- | RLGKRFKKLDLDNSGSLSVEEFMSLPELQ |
| >CALL5_HUMAN/82-110 | >CALM2_SOYBN/85-113 | >CANB1_BOVIN/132-160 | >CATR_CHLRE/102-130 |
| DLQVAFRAFDQDGDGHITVDELRRAMAGL | ELKEAFRVFDKDQNGFISAAELRHVMTNL | IVDKTIINADKDGDGRISFEEFCAVVGGL | EILKAFRLFDDDNSGTITIKDLRRVAKEL |
| >CALM2_SOYBN/48-76 | >CALM2_SOYBN/121-149 | >CANB1_BOVIN/54-82 | >CATR_CHLRE/138-166 |
| ELQDMINEVDADGNGTIDFPEFLNLMARK | EVDEMIREADVDGDGQINYEEFVKVMMAK | LVQRVIDIFDTDGNGEVDFKEFIEGVSQF | ELQEMIAEADRNDDNEIDEDEFIRIMKKT |
| >CALM2_SOYBN/12-40 | >CALM_BOVIN/85-113 | >CANB1_BOVIN/91-119 | >CAVP_BRALA/90-118 |
| EFKEAFSLFDKDGDGCITTKELGTVMRSL | EIREAFRVFDKDGNGYISAAELRHVMTNL | KLRFAFRIYDMDKDGYISNGELFQVLKMM | EILRAFKVFDANGDGVIDFDEFKFIMQKV |
| >CALM2_SOYBN/85-113 | >CALM_BOVIN/121-149 | >CANB1_BOVIN/22-50 | >CAVP_BRALA/127-155 |
| ELKEAFRVFDKDQNGFISAAELRHVMTNL | EVDEMIREADIDGDGQVNYEEFVQMMTAK | RLGKRFKKLDLDNSGSLSVEEFMSLPELQ | EVEEAMKEADEDGNGVIDIPEFMDLIKKS |
| >CALM2_SOYBN/121-149 | >CALM_PARTE/121-149 | >CATR_CHLRE/29-57 | >CBP_SACER/104-132 |
| EVDEMIREADVDGDGQINYEEFVKVMMAK | EVDEMIREADIDGDGHINYEEFVRMMVSK | EIREAFDLFDTDGSGTIDAKELKVAMRAL | VVKGTWGMCDKNADGQINADEFAAWLTAL |
| >CALM5_SOLTU/121-149 | >CALM_PARTE/12-40 | >CATR_CHLRE/102-130 | >CBP_SACER/138-166 |
| EVDEMIREADVDGDGQINYDEFVKVMMAK | EFKEAFALFDKDGDGTITTKELGTVMRSL | EILKAFRLFDDDNSGTITIKDLRRVAKEL | EAAEAFNQVDTNGNGELSLDELLTAVRDF |
| >CALM5_SOLTU/48-76 | >CALM_PARTE/85-113 | >CATR_CHLRE/138-166 | >CDC31_YEAST/133-161 |
| ELQDMINEVDADGNGTIDFPEFLNLMARK | ELIEAFKVFDRDGNGLISAAELRHVMTNL | ELQEMIAEADRNDDNEIDEDEFIRIMKKT | ELRAMIEEFDLDGDGEINENEFIAICTDS |
| >CALM5_SOLTU/12-40 | >CALM_YEAST/48-76 | >CATR_CHLRE/65-93 | >CDC31_YEAST/24-52 |
| EFKEAFSLFDKDGDGCITTKELGTVMRSL | EVNDLMNEIDVDGNHQIEFSEFLALMSRQ | EIKKMISEIDKDGSGTIDFEEFLTMMTAK | EIYEAFSLFDMNNDGFLDYHELKVAMKAL |
| >CALM5_SOLTU/85-113 | >CALM_YEAST/121-147 | >CAVP_BRALA/90-118 | >CDPK1_ARATH/527-555 |
| ELKEAFRVFDKDQNGFISAAELRHVMTNL | EVDDMLREV-SDGSGEINIQQFAALLSK- | EILRAFKVFDANGDGVIDFDEFKFIMQKV | HLFAAFTYFDKDGSGYITPDELQQACEEF |
| >CALM_BOVIN/48-76 | >CALM_YEAST/12-40 | >CAVP_BRALA/127-155 | >CDPK1_ARATH/455-483 |
| ELQDMINEVDADGNGTIDFPEFLTMMARK | EFKEAFALFDKDNNGSISSSELATVMRSL | EVEEAMKEADEDGNGVIDIPEFMDLIKKS | GLKEMFNMIDADKSGQITFEELKAGLKRV |
| >CALM_BOVIN/12-40 | >CALM_YEAST/85-113 | >CBP_SACER/104-132 | >CDPK1_ARATH/491-519 |
| EFKEAFSLFDKDGDGTITTKELGTVMRSL | ELLEAFKVFDKNGDGLISAAELKHVLTSI | VVKGTWGMCDKNADGQINADEFAAWLTAL | EILDLMQAADVDNSGTIDYKEFIAATLHL |
| >CALM_BOVIN/85-113 | >CANB1_BOVIN/132-160 | >CBP_SACER/138-166 | >CDPK1_ARATH/561-589 |
| EIREAFRVFDKDGNGYISAAELRHVMTNL | IVDKTIINADKDGDGRISFEEFCAVVGGL | EAAEAFNQVDTNGNGELSLDELLTAVRDF | RIEELMRDVDQDNDGRIDYNEFVAMMQKG |
| >CALM_BOVIN/121-149 | >CANB1_BOVIN/54-82 | >CDC31_YEAST/133-161 | >CETN2_HUMAN/68-96 |
| EVDEMIREADIDGDGQVNYEEFVQMMTAK | LVQRVIDIFDTDGNGEVDFKEFIEGVSQF | ELRAMIEEFDLDGDGEINENEFIAICTDS | EIKKMISEIDKEGTGKMNFGDFLTVMTQK |
| >CALM_CAEEL/48-76 | >CANB1_BOVIN/91-119 | >CDPK1_ARATH/527-555 | >CHP2_HUMAN/30-58 |
| ELQDMINEVDADGNGTIDFPEFLTMMARK | KLRFAFRIYDMDKDGYISNGELFQVLKMM | HLFAAFTYFDKDGSGYITPDELQQACEEF | RLHHRFRALDRNKKGYLSRMDLQQIGALA |
| >CALM_CAEEL/12-40 | >CANB1_BOVIN/22-50 | >CDPK1_ARATH/455-483 | >CHP2_HUMAN/156-184 |
| EFKEAFSLFDKDGDGTITTKELGTVMRSL | RLGKRFKKLDLDNSGSLSVEEFMSLPELQ | GLKEMFNMIDADKSGQITFEELKAGLKRV | IADRTVQEADEDGDGAVSFVEFTKSLEKM |
| >CALM_CAEEL/85-113 | >CATR_CHLRE/29-57 | >CDPK1_ARATH/491-519 | >CHP2_HUMAN/115-143 |
| EIREAFRVFDKDGNGFISAAELRHVMTNL | EIREAFDLFDTDGSGTIDAKELKVAMRAL | EILDLMQAADVDNSGTIDYKEFIAATLHL | KLHYAFQLYDLDRDGKISRHEMLQVLRLM |
| >CALM_CAEEL/121-149 | >CATR_CHLRE/102-130 | >CDPK1_ARATH/561-589 | >CLSS_HAEMA/50-78 |
| EVDEMIREADIDGDGQVNYEEFVTMMTTK | EILKAFRLFDDDNSGTITIKDLRRVAKEL | RIEELMRDVDQDNDGRIDYNEFVAMMQKG | ASAKLIKMADKNSDGKISKEEFLNANAEL |
| >CALM_CHICK/48-76 | >CATR_CHLRE/138-166 | >CDPK_SOYBN/445-473 | >CLSS_HAEMA/8-36 |
| ELQDMINEVDADGNGTIDFPEFLTMMARK | ELQEMIAEADRNDDNEIDEDEFIRIMKKT | HIDDMIKEIDQDNDGQIDYGEFAAMMRKG | ELEAAFKKLDANGDGYVTALELQTFMVTL |
| >CALM_CHICK/12-40 | >CATR_CHLRE/65-93 | >CHP1_HUMAN/155-183 | >CSEN_HUMAN/130-158 |
| EFKEAFSLFDKDGDGTITTKELGTVMRSL | EIKKMISEIDKDGSGTIDFEEFLTMMTAK | IADRTIQEADQDGDSAISFTEFVKVLEKV | YAHFLFNAFDADGNGAIHFEDFVVGLSIL |
| >CALM_CHICK/85-113 | >CAVP_BRALA/90-118 | >CHP1_HUMAN/114-142 | >CSEN_HUMAN/166-194 |
| EIREAFRVFDKDGNGYISAAELRHVMTNL | EILRAFKVFDANGDGVIDFDEFKFIMQKV | KLHFAFRLYDLDKDEKISRDELLQVLRMM | KLKWAFNLYDINKDGYITKEEMLAIMKSI |
| >CALM_CHICK/121-149 | >CAVP_BRALA/127-155 | >CHP2_HUMAN/30-58 | >CSEN_HUMAN/214-242 |
| EVDEMIREADIDGDGQVNYEEFVQMMTAK | EVEEAMKEADEDGNGVIDIPEFMDLIKKS | RLHHRFRALDRNKKGYLSRMDLQQIGALA | HVERFFEKMDRNQDGVVTIEEFLEACQKD |
| >CALM_DROME/12-40 | >CBP_SACER/104-132 | >CLSS_HAEMA/50-78 | >GUC1A_CHICK/54-82 |
| EFKEAFSLFDKDGDGTITTKELGTVMRSL | VVKGTWGMCDKNADGQINADEFAAWLTAL | ASAKLIKMADKNSDGKISKEEFLNANAEL | YVEQMFETFDFNKDGYIDFMEYVAALSLV |
| >CALM_DROME/85-113 | >CBP_SACER/138-166 | >CLSS_HAEMA/8-36 | >GUC1A_CHICK/90-118 |
| EIREAFRVFDKDGNGFISAAELRHVMTNL | EAAEAFNQVDTNGNGELSLDELLTAVRDF | ELEAAFKKLDANGDGYVTALELQTFMVTL | KLRWYFKLYDVDGNGCIDRGELLNIIKAI |
| >CALM_DROME/121-149 | >CDC31_YEAST/133-161 | >CSEN_HUMAN/130-158 | >GUC1A_CHICK/133-161 |
| EVDEMIREADIDGDGQVNYEEFVTMMTSK | ELRAMIEEFDLDGDGEINENEFIAICTDS | YAHFLFNAFDADGNGAIHFEDFVVGLSIL | FTNMVFDKIDINGDGELSLEEFMEGVQKD |
| >CALM_DROME/48-76 | >CDC31_YEAST/24-52 | >CSEN_HUMAN/166-194 | >GUC1B_BOVIN/149-177 |
| ELQDMINEVDADGNGTIDFPEFLTMMARK | EIYEAFSLFDMNNDGFLDYHELKVAMKAL | KLKWAFNLYDINKDGYITKEEMLAIMKSI | VVDRIFLLVDENGDGQLSLNEFVEGARRD |
| >CALM_HUMAN/85-113 | >CDC31_YEAST/97-125 | >CSEN_HUMAN/214-242 | >GUC1C_HUMAN/56-84 |
| EIREAFRVFDKDGNGYISAAELRHVMTNL | EIKRAFQLFDDDHTGKISIKNLRRVAKEL | HVERFFEKMDRNQDGVVTIEEFLEACQKD | HIDQVYNTFDTNKDGFVDFLEFIAAVNLI |
| >CALM_HUMAN/121-149 | >CDPK1_ARATH/527-555 | >GUC1A_CHICK/54-82 | >MLR_AEQIR/20-48 |
| EVDEMIREADIDGDGQVNYEEFVQMMTAK | HLFAAFTYFDKDGSGYITPDELQQACEEF | YVEQMFETFDFNKDGYIDFMEYVAALSLV | EMKEAFSMIDVDRDGFVSKEDIKAISEQL |
| >CALM_HUMAN/48-76 | >CDPK1_ARATH/455-483 | >GUC1A_CHICK/90-118 | >MLR_AEQIR/89-117 |
| ELQDMINEVDADGNGTIDFPEFLTMMARK | GLKEMFNMIDADKSGQITFEELKAGLKRV | KLRWYFKLYDVDGNGCIDRGELLNIIKAI | TIRNAFAMFDEQETKKLNIEYIKDLLENM |
| >CALM_HUMAN/12-40 | >CDPK1_ARATH/491-519 | >GUC1A_CHICK/133-161 | >MLR_PHYPO/6-34 |
| EFKEAFSLFDKDGDGTITTKELGTVMRSL | EILDLMQAADVDNSGTIDYKEFIAATLHL | FTNMVFDKIDINGDGELSLEEFMEGVQKD | QIQECFQIFDKDNDGKVSIEELGSALRSL |
| >CALM_MOUSE/48-76 | >CDPK1_ARATH/561-589 | >GUC1B_BOVIN/149-177 | >MLR_PHYPO/113-141 |
| ELQDMINEVDADGNGTIDFPEFLTMMARK | RIEELMRDVDQDNDGRIDYNEFVAMMQKG | VVDRIFLLVDENGDGQLSLNEFVEGARRD | EVEELMKEVSVSGDGAINYESFVDMLVTG |
| >CALM_MOUSE/12-40 | >CDPK_SOYBN/375-403 | >GUC1C_HUMAN/56-84 | >MLR_PHYPO/77-105 |
| EFKEAFSLFDKDGDGTITTKELGTVMRSL | EIKDLMDAADIDKSGTIDYGEFIAATVHL | HIDQVYNTFDTNKDGFVDFLEFIAAVNLI | EMLDAFRALDKEGNGTIQEAELRQLLLNL |
| >CALM_MOUSE/85-113 | >CDPK_SOYBN/445-473 | >KCIP1_HUMAN/185-213 | >NCALD_BOVIN/148-176 |
| EIREAFRVFDKDGNGYISAAELRHVMTNL | HIDDMIKEIDQDNDGQIDYGEFAAMMRKG | HVDVFFQKMDKNKDGIVTLDEFLESCQED | RTEKIFRQMDTNRDGKLSLEEFIRGAKSD |
| >CALM_MOUSE/121-149 | >CDPK_SOYBN/411-439 | >MLR_AEQIR/20-48 | >NCS1_HUMAN/64-92 |
| EVDEMIREADIDGDGQVNYEEFVQMMTAK | NLVSAFSYFDKDGSGYITLDEIQQACKDF | EMKEAFSMIDVDRDGFVSKEDIKAISEQL | FATFVFNVFDENKDGRIEFSEFIQALSVT |
| >CALM_PARTE/121-149 | >CDPK_SOYBN/339-367 | >MLR_AEQIR/89-117 | >NCS1_YEAST/148-176 |
| EVDEMIREADIDGDGHINYEEFVRMMVSK | GLKELFKMIDTDNSGTITFDELKDGLKRV | TIRNAFAMFDEQETKKLNIEYIKDLLENM | RVKKIFKLMDKNEDGYITLDEFREGSKVD |
| >CALM_PARTE/48-76 | >CETN2_HUMAN/141-169 | >MLR_PHYPO/6-34 | >OBL_OBELO/150-171 |
| ELQDMINEVDADGNGTIDFPEFLSLMARK | ELQEMIDEADRDGDGEVSEQEFLRIMKKT | QIQECFQIFDKDNDGKVSIEELGSALRSL | DCEATFRHCDLDNSGDLDVDEM------- |
| >CALM_PARTE/12-40 | >CETN2_HUMAN/68-96 | >MLR_PHYPO/113-141 | >OBL_OBELO/116-142 |
| EFKEAFALFDKDGDGTITTKELGTVMRSL | EIKKMISEIDKEGTGKMNFGDFLTVMTQK | EVEELMKEVSVSGDGAINYESFVDMLVTG | --DAVFDIFDKDGSGTITLDEWKAYGKIS |
| >CALM_PARTE/85-113 | >CETN2_HUMAN/32-60 | >MLR_PHYPO/77-105 | >OBL_OBELO/21-49 |
| ELIEAFKVFDRDGNGLISAAELRHVMTNL | EIREAFDLFDADGTGTIDVKELKVAMRAL | EMLDAFRALDKEGNGTIQEAELRQLLLNL | RHKHMFDFLDINGNGKITLDEIVSKASDD |
| >CALM_RAT/48-76 | >CETN2_HUMAN/105-133 | >MLR_TODPA/17-45 | >ONCO_RAT/43-71 |
| ELQDMINEVDADGNGTIDFPEFLTMMARK | EILKAFKLFDDDETGKISFKNLKRVAKEL | ELKEAFTMIDQDRDGFIGMEDLKDMFSSL | QVKDIFRFIDNDQSGYLDGDELKYFLQKF |
| >CALM_RAT/12-40 | >CHP1_HUMAN/155-183 | >MLR_TODPA/86-114 | >PDCD6_HUMAN/94-122 |
| EFKEAFSLFDKDGDGTITTKELGTVMRSL | IADRTIQEADQDGDSAISFTEFVKVLEKV | ALRNAFSMFDEDGQGFIPEDYLKDLLENM | DWQNVFRTYDRDNSGMIDKNELKQALSGF |
| >CALM_RAT/85-113 | >CHP1_HUMAN/114-142 | >NCALD_BOVIN/148-176 | >PDCD6_HUMAN/27-55 |
| EIREAFRVFDKDGNGYISAAELRHVMTNL | KLHFAFRLYDLDKDEKISRDELLQVLRMM | RTEKIFRQMDTNRDGKLSLEEFIRGAKSD | FLWNVFQRVDKDRSGVISDTELQQALSNG |
| >CALM_RAT/121-149 | >CHP2_HUMAN/30-58 | >NCALD_BOVIN/64-92 | >PDCD6_HUMAN/160-176 |
| EVDEMIREADIDGDGQVNYEEFVQMMTAK | RLHHRFRALDRNKKGYLSRMDLQQIGALA | FAEHVFRTFDANGDGTIDFREFIIALSVT | RLTDIFRRYDTDQDGWI------------ |
| >CALM_XENLA/48-76 | >CHP2_HUMAN/156-184 | >NCALD_BOVIN/100-128 | >PDCD6_HUMAN/130-158 |
| ELQDMINEVDADGNGTIDFPEFLTMMARK | IADRTVQEADEDGDGAVSFVEFTKSLEKM | KLKWAFSMYDLDGNGYISKAEMLEIVQAI | FHDILIRKFDRQGRGQIAFDDFIQGCIVL |
| >CALM_XENLA/12-40 | >CHP2_HUMAN/115-143 | >NCS1_HUMAN/148-176 | >POLC3_CHEAL/47-75 |
| EFKEAFSLFDKDGDGTITTKELGTVMRSL | KLHYAFQLYDLDRDGKISRHEMLQVLRLM | RVDRIFAMMDKNADGKLTLQEFQEGSKAD | EVRRMMAEIDTDGDGFISFDEFTDFARAN |
| >CALM_XENLA/85-113 | >CLSS_HAEMA/50-78 | >NCS1_YEAST/64-92 | >PRVA_ESOLU/80-108 |
| EIREAFRVFDKDGNGYISAAELRHVMTNL | ASAKLIKMADKNSDGKISKEEFLNANAEL | FANHLFTVFDKDNNGFIHFEEFITVLSTT | ETKAFLKAADKDGDGKIGIDEFETLVHEA |
| >CALM_XENLA/121-149 | >CLSS_HAEMA/8-36 | >OBL_OBELO/150-171 | >PRVA_HUMAN/43-71 |
| EVDEMIREADIDGDGQVNYEEFVQMMTAK | ELEAAFKKLDANGDGYVTALELQTFMVTL | DCEATFRHCDLDNSGDLDVDEM------- | DVKKVFHMLDKDKSGFIEEDELGFILKGF |
| >CALM_YEAST/48-76 | >CSEN_HUMAN/130-158 | >OBL_OBELO/116-142 | >Q7ZZB9_ONCMY/96-124 |
| EVNDLMNEIDVDGNHQIEFSEFLALMSRQ | YAHFLFNAFDADGNGAIHFEDFVVGLSIL | --DAVFDIFDKDGSGTITLDEWKAYGKIS | ELADLFCMFDKNADGYIDLQELKVMLEAT |
| >CALM_YEAST/121-147 | >CSEN_HUMAN/166-194 | >OBL_OBELO/21-49 | >Q8WSQ4_PHYPO/56-84 |
| EVDDMLREV-SDGSGEINIQQFAALLSK- | KLKWAFNLYDINKDGYITKEEMLAIMKSI | RHKHMFDFLDINGNGKITLDEIVSKASDD | AFNEMFNEADATGNGKIQFPEFLSMMGRR |
| >CALM_YEAST/12-40 | >CSEN_HUMAN/214-242 | >ONCO_RAT/43-71 | >Q8WSQ4_PHYPO/93-121 |
| EFKEAFALFDKDNNGSISSSELATVMRSL | HVERFFEKMDRNQDGVVTIEEFLEACQKD | QVKDIFRFIDNDQSGYLDGDELKYFLQKF | ILRQAFRTFDPEGTGYIPKAALQDALLNL |
| >CALM_YEAST/85-113 | >CSEN_MOUSE/214-242 | >PDCD6_HUMAN/94-122 | >Q9XZV2_EUPOC/64-92 |
| ELLEAFKVFDKNGDGLISAAELKHVLTSI | HVERFFQKMDRNQDGVVTIDEFLETCQKD | DWQNVFRTYDRDNSGMIDKNELKQALSGF | EILELMNEYDREGNGYIGFDDFLDIMTEK |
| >CANB1_BOVIN/132-160 | >GUC1A_CHICK/54-82 | >PDCD6_HUMAN/27-55 | >RECO_BOVIN/65-93 |
| IVDKTIINADKDGDGRISFEEFCAVVGGL | YVEQMFETFDFNKDGYIDFMEYVAALSLV | FLWNVFQRVDKDRSGVISDTELQQALSNG | YAQHVFRSFDANSDGTLDFKEYVIALHMT |
| >CANB1_BOVIN/54-82 | >GUC1A_CHICK/90-118 | >PDCD6_HUMAN/160-176 | >S100B_BOVIN/53-81 |
| LVQRVIDIFDTDGNGEVDFKEFIEGVSQF | KLRWYFKLYDVDGNGCIDRGELLNIIKAI | RLTDIFRRYDTDQDGWI------------ | VVDKVMETLDSDGDGECDFQEFMAFVAMI |
| >CANB1_BOVIN/91-119 | >GUC1A_CHICK/133-161 | >PDCD6_HUMAN/130-158 | >S100G_BOVIN/49-77 |
| KLRFAFRIYDMDKDGYISNGELFQVLKMM | FTNMVFDKIDINGDGELSLEEFMEGVQKD | FHDILIRKFDRQGRGQIAFDDFIQGCIVL | TLDELFEELDKNGDGEVSFEEFQVLVKKI |
| >CANB1_BOVIN/22-50 | >GUC1B_BOVIN/60-88 | >POLC3_CHEAL/47-75 | >S10AB_PIG/57-85 |
| RLGKRFKKLDLDNSGSLSVEEFMSLPELQ | YVEAMFRAFDTNGDNTIDFLEYVAALNLV | EVRRMMAEIDTDGDGFISFDEFTDFARAN | VLDRMMKKLDLDSDGQLDFQEFLNLIGGL |
| >CANB1_HUMAN/54-82 | >GUC1B_BOVIN/96-124 | >POLC3_CHEAL/12-40 | >TNNC2_CHICK/58-86 |
| LVQRVIDIFDTDGNGEVDFKEFIEGVSQF | KLKWTFKIYDKDRNGCIDRQELLDIVESI | DRERIFKRFDTNGDGKISSSELGDALKTL | ELDAIIEEVDEDGSGTIDFEEFLVMMVRQ |
| >CANB1_HUMAN/91-119 | >GUC1B_BOVIN/149-177 | >PRVA_ESOLU/80-108 |  |
| KLRFAFRIYDMDKDGYISNGELFQVLKMM | VVDRIFLLVDENGDGQLSLNEFVEGARRD | ETKAFLKAADKDGDGKIGIDEFETLVHEA |  |
| >CANB1_HUMAN/22-50 | >GUC1C_HUMAN/56-84 | >PRVA_ESOLU/41-69 |  |
| RLGKRFKKLDLDNSGSLSVEEFMSLPELQ | HIDQVYNTFDTNKDGFVDFLEFIAAVNLI | DVKKVFKAIDADASGFIEEEELKFVLKSF |  |
| >CANB1_HUMAN/132-160 | >GUC1C_HUMAN/92-120 | >Q7ZZB9_ONCMY/132-160 |  |
| IVDKTIINADKDGDGRISFEEFCAVVGGL | KLKWYFKLYDADGNGSIDKNELLDMFMAV | DIEELMKDGDKNNDGKIDYDEFLEFMKGV |  |
| >CATR_CHLRE/29-57 | >GUC1C_HUMAN/134-162 | >Q7ZZB9_ONCMY/96-124 |  |
| EIREAFDLFDTDGSGTIDAKELKVAMRAL | FINLVFHKIDINNDGELTLEEFINGMAKD | ELADLFCMFDKNADGYIDLQELKVMLEAT |  |
| >CATR_CHLRE/102-130 | >KCIP1_HUMAN/101-129 | >Q868D4_9HEMI/128-156 |  |
| EILKAFRLFDDDNSGTITIKDLRRVAKEL | YAHYLFNAFDTTQTGSVKFEDFVTALSIL | DLDAMIDEIDADGSGTVDFEEFMGVMTGG |  |
| >CATR_CHLRE/138-166 | >KCIP1_HUMAN/137-165 | >Q868D4_9HEMI/92-120 |  |
| ELQEMIAEADRNDDNEIDEDEFIRIMKKT | KLRWTFNLYDINKDGYINKEEMMDIVKAI | ELREAFRLYDKEGNGYISTDVMREILAEL |  |
| >CATR_CHLRE/65-93 | >KCIP1_HUMAN/185-213 | >Q8WSQ4_PHYPO/56-84 |  |
| EIKKMISEIDKDGSGTIDFEEFLTMMTAK | HVDVFFQKMDKNKDGIVTLDEFLESCQED | AFNEMFNEADATGNGKIQFPEFLSMMGRR |  |
| >CAVP_BRALA/90-118 | >KCIP4_MOUSE/160-188 | >Q8WSQ4_PHYPO/20-48 |  |
| EILRAFKVFDANGDGVIDFDEFKFIMQKV | KLNWAFNLYDINKDGYITKEEMLDIMKAI | EFKEAFELFDSERTGFITKEGLQTVLKQF |  |
| >CAVP_BRALA/127-155 | >KCIP4_MOUSE/208-236 | >Q8WSQ4_PHYPO/93-121 |  |
| EVEEAMKEADEDGNGVIDIPEFMDLIKKS | HVETFFQKMDKNKDGVVTIDEFIESCQKD | ILRQAFRTFDPEGTGYIPKAALQDALLNL |  |
| >CBP_SACER/104-132 | >KCIP4_MOUSE/124-152 | >Q9XZV2_EUPOC/64-92 |  |
| VVKGTWGMCDKNADGQINADEFAAWLTAL | YAHFLFNAFDTDHNGAVSFEDFIKGLSIL | EILELMNEYDREGNGYIGFDDFLDIMTEK |  |
| >CBP_SACER/138-166 | >MLR_AEQIR/20-48 | >Q9XZV2_EUPOC/137-165 |  |
| EAAEAFNQVDTNGNGELSLDELLTAVRDF | EMKEAFSMIDVDRDGFVSKEDIKAISEQL | ELQAMIDEFDKDQDGEISEQEFLNIMKQT |  |
| >CDC31_YEAST/133-161 | >MLR_AEQIR/89-117 | >RECO_HUMAN/65-93 |  |
| ELRAMIEEFDLDGDGEINENEFIAICTDS | TIRNAFAMFDEQETKKLNIEYIKDLLENM | YAQHVFRSFDSNLDGTLDFKEYVIALHMT |  |
| >CDC31_YEAST/24-52 | >MLR_PHYPO/6-34 | >S100B_BOVIN/53-81 |  |
| EIYEAFSLFDMNNDGFLDYHELKVAMKAL | QIQECFQIFDKDNDGKVSIEELGSALRSL | VVDKVMETLDSDGDGECDFQEFMAFVAMI |  |
| >CDC31_YEAST/97-125 | >MLR_PHYPO/113-141 | >S100G_BOVIN/49-77 |  |
| EIKRAFQLFDDDHTGKISIKNLRRVAKEL | EVEELMKEVSVSGDGAINYESFVDMLVTG | TLDELFEELDKNGDGEVSFEEFQVLVKKI |  |
| >CDPK1_ARATH/527-555 | >MLR_PHYPO/77-105 | >S10A1_BOVIN/54-82 |  |
| HLFAAFTYFDKDGSGYITPDELQQACEEF | EMLDAFRALDKEGNGTIQEAELRQLLLNL | AVDKVMKELDENGDGEVDFQEYVVLVAAL |  |
| >CDPK1_ARATH/455-483 | >MLR_TODPA/17-45 | >S10AB_PIG/57-85 |  |
| GLKEMFNMIDADKSGQITFEELKAGLKRV | ELKEAFTMIDQDRDGFIGMEDLKDMFSSL | VLDRMMKKLDLDSDGQLDFQEFLNLIGGL |  |
| >CDPK1_ARATH/491-519 | >MLR_TODPA/86-114 | >TNNC2_RABIT/95-123 |  |
| EILDLMQAADVDNSGTIDYKEFIAATLHL | ALRNAFSMFDEDGQGFIPEDYLKDLLENM | ELAECFRIFDRNADGYIDAEELAEIFRAS |  |
| >CDPK1_ARATH/561-589 | >NCALD_BOVIN/148-176 | >CABP1_HUMAN/200-227 |  |
| RIEELMRDVDQDNDGRIDYNEFVAMMQKG | RTEKIFRQMDTNRDGKLSLEEFIRGAKSD | DIEEIIRDVDLNGDGRVDFEEFVRMM-SR |  |
| >CDPK_SOYBN/375-403 | >NCALD_BOVIN/64-92 | >CABP1_HUMAN/163-191 |  |
| EIKDLMDAADIDKSGTIDYGEFIAATVHL | FAEHVFRTFDANGDGTIDFREFIIALSVT | ELRDAFREFDTNGDGEISTSELREAMRKL |  |
| >CDPK_SOYBN/445-473 | >NCALD_BOVIN/100-128 | >CABP1_HUMAN/86-114 |  |
| HIDDMIKEIDQDNDGQIDYGEFAAMMRKG | KLKWAFSMYDLDGNGYISKAEMLEIVQAI | ELREAFREFDKDKDGYINCRDLGNCMRTM |  |
| >CDPK_SOYBN/411-439 | >NCS1_HUMAN/64-92 | >CALB1_RAT/15-43 |  |
| NLVSAFSYFDKDGSGYITLDEIQQACKDF | FATFVFNVFDENKDGRIEFSEFIQALSVT | QFFEIWLHFDADGSGYLEGKELQNLIQEL |  |
| >CDPK_SOYBN/339-367 | >NCS1_HUMAN/100-128 | >CALB1_RAT/190-218 |  |
| GLKELFKMIDTDNSGTITFDELKDGLKRV | KLRWAFKLYDLDNDGYITRNEMLDIVDAI | EFNKAFELYDQDGNGYIDENELDALLKDL |  |
| >CETN2_HUMAN/141-169 | >NCS1_HUMAN/148-176 | >CALB1_RAT/102-130 |  |
| ELQEMIDEADRDGDGEVSEQEFLRIMKKT | RVDRIFAMMDKNADGKLTLQEFQEGSKAD | EFMKTWRKYDTDHSGFIETEELKNFLKDL |  |
| >CETN2_HUMAN/68-96 | >NCS1_YEAST/100-128 | >CALB1_RAT/146-174 |  |
| EIKKMISEIDKEGTGKMNFGDFLTVMTQK | KLSWAFELYDLNHDGYITFDEMLTIVASV | YTDLMLKLFDSNNDGKLELTEMARLLPVQ |  |
| >CETN2_HUMAN/32-60 | >NCS1_YEAST/148-176 | >CALBP_ENTHI/37-65 |  |
| EIREAFDLFDADGTGTIDVKELKVAMRAL | RVKKIFKLMDKNEDGYITLDEFREGSKVD | LLQLIFKSIDADGNGEIDQNEFAKFYGSI |  |
| >CETN2_HUMAN/105-133 | >NCS1_YEAST/64-92 | >CALBP_ENTHI/108-134 |  |
| EILKAFKLFDDDETGKISFKNLKRVAKEL | FANHLFTVFDKDNNGFIHFEEFITVLSTT | KVAEQVMKADANGDGYITLEEFLEF..SL |  |
| >CHP1_HUMAN/155-183 | >OBL_OBELO/150-171 | >CALBP_ENTHI/76-104 |  |
| IADRTIQEADQDGDSAISFTEFVKVLEKV | DCEATFRHCDLDNSGDLDVDEM------- | GLKVLYKLMDVDGDGKLTKEEVTSFFKKH |  |
| >CHP1_HUMAN/114-142 | >OBL_OBELO/116-142 | >CALBP_ENTHI/1-29 |  |
| KLHFAFRLYDLDKDEKISRDELLQVLRMM | --DAVFDIFDKDGSGTITLDEWKAYGKIS | MAEALFKEIDVNGDGAVSYEEVKAFVSKK |  |
| >CHP1_HUMAN/155-183 | >OBL_OBELO/21-49 | >CALL3_HUMAN/121-149 |  |
| IADRTIQEADQDGDSAISFTEFVKVLEKV | RHKHMFDFLDINGNGKITLDEIVSKASDD | EVDEMIRAADTDGDGQVNYEEFVRVLVSK |  |
| >CHP2_HUMAN/30-58 | >ONCO_RAT/82-109 | >CALL3_HUMAN/48-76 |  |
| RLHHRFRALDRNKKGYLSRMDLQQIGALA | ETKSLMDAADNDGDGKIGADEFQEMVHS- | ELRDMMSEIDRDGNGTVDFPEFLGMMARK |  |
| >CHP2_HUMAN/156-184 | >ONCO_RAT/43-71 | >CALL3_HUMAN/12-40 |  |
| IADRTVQEADEDGDGAVSFVEFTKSLEKM | QVKDIFRFIDNDQSGYLDGDELKYFLQKF | EFKEAFSLFDKDGDGCITTRELGTVMRSL |  |
| >CHP2_HUMAN/115-143 | >PDCD6_HUMAN/94-122 | >CALL3_HUMAN/85-113 |  |
| KLHYAFQLYDLDRDGKISRHEMLQVLRLM | DWQNVFRTYDRDNSGMIDKNELKQALSGF | EIREAFRVFDKDGNGFVSAAELRHVMTRL |  |
| >CLSS_HAEMA/50-78 | >PDCD6_HUMAN/27-55 | >CALL5_HUMAN/12-40 |  |
| ASAKLIKMADKNSDGKISKEEFLNANAEL | FLWNVFQRVDKDRSGVISDTELQQALSNG | QYKKAFSAVDTDGNGTINAQELGAALKAT |  |
| >CLSS_HAEMA/8-36 | >PDCD6_HUMAN/160-176 | >CALL5_HUMAN/48-76 |  |
| ELEAAFKKLDANGDGYVTALELQTFMVTL | RLTDIFRRYDTDQDGWI------------ | QLRKLISEVDSDGDGEISFQEFLTAARKA |  |
| >CSEN_HUMAN/130-158 | >PDCD6_HUMAN/130-158 | >CALL5_HUMAN/82-110 |  |
| YAHFLFNAFDADGNGAIHFEDFVVGLSIL | FHDILIRKFDRQGRGQIAFDDFIQGCIVL | DLQVAFRAFDQDGDGHITVDELRRAMAGL |  |
| >CSEN_HUMAN/166-194 | >POLC3_CHEAL/47-75 | >CALM_YEAST/48-76 |  |
| KLKWAFNLYDINKDGYITKEEMLAIMKSI | EVRRMMAEIDTDGDGFISFDEFTDFARAN | EVNDLMNEIDVDGNHQIEFSEFLALMSRQ |  |
| >CSEN_HUMAN/214-242 | >POLC3_CHEAL/12-40 | >CALM_YEAST/121-147 |  |
| HVERFFEKMDRNQDGVVTIEEFLEACQKD | DRERIFKRFDTNGDGKISSSELGDALKTL | EVDDMLREV-SDGSGEINIQQFAALLSK- |  |
| >CSEN_MOUSE/130-158 | >POLC4_BETVE/11-39 | >CANB1_BOVIN/132-160 |  |
| YAHFLFNAFDADGNGAIHFEDFVVGLSIL | ERERIFKRFDANGDGKISAAELGEALKTL | IVDKTIINADKDGDGRISFEEFCAVVGGL |  |
| >CSEN_MOUSE/166-194 | >POLC4_BETVE/46-74 | >CANB1_BOVIN/54-82 |  |
| KLKWAFNLYDINKDGCITKEEMLAIMKSI | EVKHMMAEIDTDGDGFISFQEFTDFGRAN | LVQRVIDIFDTDGNGEVDFKEFIEGVSQF |  |
| >CSEN_MOUSE/214-242 | >POLC7_PHLPR/4-32 | >CANB1_BOVIN/91-119 |  |
| HVERFFQKMDRNQDGVVTIDEFLETCQKD | DMERIFKRFDTNGDGKISLSELTDALRTL | KLRFAFRIYDMDKDGYISNGELFQVLKMM |  |
| >GUC1A_CHICK/54-82 | >POLC7_PHLPR/39-67 |  |  |
| YVEQMFETFDFNKDGYIDFMEYVAALSLV | EVQRMMAEIDTDGDGFIDFNEFISFCNAN |  |  |
| >GUC1A_CHICK/90-118 | >PRVA_ESOLU/80-108 |  |  |
| KLRWYFKLYDVDGNGCIDRGELLNIIKAI | ETKAFLKAADKDGDGKIGIDEFETLVHEA |  |  |
| >GUC1A_CHICK/133-161 | >PRVA_ESOLU/41-69 |  |  |
| FTNMVFDKIDINGDGELSLEEFMEGVQKD | DVKKVFKAIDADASGFIEEEELKFVLKSF |  |  |
| >GUC1B_BOVIN/60-88 | >PRVA_HUMAN/82-110 |  |  |
| YVEAMFRAFDTNGDNTIDFLEYVAALNLV | ETKMLMAAGDKDGDGKIGVDEFSTLVAES |  |  |
| >GUC1B_BOVIN/96-124 | >PRVA_HUMAN/43-71 |  |  |
| KLKWTFKIYDKDRNGCIDRQELLDIVESI | DVKKVFHMLDKDKSGFIEEDELGFILKGF |  |  |
| >GUC1B_BOVIN/149-177 | >PRVA_TRISE/81-109 |  |  |
| VVDRIFLLVDENGDGQLSLNEFVEGARRD | ETKALLAAGDSDHDGKIGADEFAKMVAQA |  |  |
| >GUC1C_HUMAN/56-84 | >PRVA_TRISE/42-70 |  |  |
| HIDQVYNTFDTNKDGFVDFLEFIAAVNLI | QVKEVFEILDKDQSGFIEEEELKGVLKGF |  |  |
| >GUC1C_HUMAN/92-120 | >PRVB_CYPCA/81-108 |  |  |
| KLKWYFKLYDADGNGSIDKNELLDMFMAV | ETKTFLKAGDSDGDGKIGVDEFTALVKA- |  |  |
| >GUC1C_HUMAN/134-162 | >PRVB_CYPCA/42-70 |  |  |
| FINLVFHKIDINNDGELTLEEFINGMAKD | DVKKAFAIIDQDKSGFIEEDELKLFLQNF |  |  |
| >KCIP1_HUMAN/101-129 | >PRVB_ESOLU/80-107 |  |  |
| YAHYLFNAFDTTQTGSVKFEDFVTALSIL | ETKAFLADGDKDGDGMIGVDEFAAMIKA- |  |  |
| >KCIP1_HUMAN/137-165 | >Q26068_PLAMG/20-48 |  |  |
| KLRWTFNLYDINKDGYINKEEMMDIVKAI | EMKEAFTMIDQNRDGFIDINDLKEMFSSL |  |  |
| >KCIP1_HUMAN/185-213 | >Q39890_SOYBN/121-149 |  |  |
| HVDVFFQKMDKNKDGIVTLDEFLESCQED | EVEQMIKEADLDGDGQVNYEEFVKMMMTV |  |  |
| >KCIP1_RAT/101-129 | >Q39890_SOYBN/48-76 |  |  |
| YAHYLFNAFDTTQTGSVKFEDFVTALSIL | ELQDMISEVDADGNGTIEFDEFLSLMAKK |  |  |
| >KCIP1_RAT/137-165 | >Q39890_SOYBN/12-40 |  |  |
| KLRWTFNLYDINKDGYINKEEMMDIVKAI | DFKEAFGLFDKDGDGCITVEELATVIRSL |  |  |
| >KCIP1_RAT/185-213 | >Q39890_SOYBN/85-113 |  |  |
| HVDVFFQKMDKNKDGIVTLDEFLESCQED | ELKEAFKVFDKDQNGYISASELRHVMINL |  |  |
| >KCIP4_MOUSE/160-188 | >Q7ZZB9_ONCMY/56-84 |  |  |
| KLNWAFNLYDINKDGYITKEEMLDIMKAI | ELQEMIDEVDEDGSGTVDFDEFLVMMVRC |  |  |
| >KCIP4_MOUSE/208-236 | >Q7ZZB9_ONCMY/132-160 |  |  |
| HVETFFQKMDKNKDGVVTIDEFIESCQKD | DIEELMKDGDKNNDGKIDYDEFLEFMKGV |  |  |
| >KCIP4_MOUSE/124-152 | >Q7ZZB9_ONCMY/96-124 |  |  |
| YAHFLFNAFDTDHNGAVSFEDFIKGLSIL | ELADLFCMFDKNADGYIDLQELKVMLEAT |  |  |
| >MLR_AEQIR/20-48 | >Q868D4_9HEMI/128-156 |  |  |
| EMKEAFSMIDVDRDGFVSKEDIKAISEQL | DLDAMIDEIDADGSGTVDFEEFMGVMTGG |  |  |
| >MLR_AEQIR/89-117 | >Q868D4_9HEMI/92-120 |  |  |
| TIRNAFAMFDEQETKKLNIEYIKDLLENM | ELREAFRLYDKEGNGYISTDVMREILAEL |  |  |
| >MLR_PHYPO/6-34 | >Q8WSQ4_PHYPO/56-84 |  |  |
| QIQECFQIFDKDNDGKVSIEELGSALRSL | AFNEMFNEADATGNGKIQFPEFLSMMGRR |  |  |
| >MLR_PHYPO/113-141 | >Q8WSQ4_PHYPO/20-48 |  |  |
| EVEELMKEVSVSGDGAINYESFVDMLVTG | EFKEAFELFDSERTGFITKEGLQTVLKQF |  |  |
| >MLR_PHYPO/77-105 | >Q8WSQ4_PHYPO/93-121 |  |  |
| EMLDAFRALDKEGNGTIQEAELRQLLLNL | ILRQAFRTFDPEGTGYIPKAALQDALLNL |  |  |
| >MLR_TODPA/17-45 | >Q9XZV2_EUPOC/28-56 |  |  |
| ELKEAFTMIDQDRDGFIGMEDLKDMFSSL | EIKEAFDLFDTNKTGSIDYHELKVAMRAL |  |  |
| >MLR_TODPA/86-114 | >Q9XZV2_EUPOC/101-129 |  |  |
| ALRNAFSMFDEDGQGFIPEDYLKDLLENM | EILKAFKVFDEDNSGKISLRNLKRVAKEL |  |  |
| >NCALD_BOVIN/148-176 | >Q9XZV2_EUPOC/64-92 |  |  |
| RTEKIFRQMDTNRDGKLSLEEFIRGAKSD | EILELMNEYDREGNGYIGFDDFLDIMTEK |  |  |
| >NCALD_BOVIN/64-92 | >Q9XZV2_EUPOC/137-165 |  |  |
| FAEHVFRTFDANGDGTIDFREFIIALSVT | ELQAMIDEFDKDQDGEISEQEFLNIMKQT |  |  |
| >NCALD_BOVIN/100-128 | >RECO_BOVIN/101-129 |  |  |
| KLKWAFSMYDLDGNGYISKAEMLEIVQAI | KLEWAFSLYDVDGNGTISKNEVLEIVTAI |  |  |
| >NCS1_HUMAN/64-92 | >RECO_BOVIN/65-93 |  |  |
| FATFVFNVFDENKDGRIEFSEFIQALSVT | YAQHVFRSFDANSDGTLDFKEYVIALHMT |  |  |
| >NCS1_HUMAN/100-128 | >S100B_BOVIN/53-81 |  |  |
| KLRWAFKLYDLDNDGYITRNEMLDIVDAI | VVDKVMETLDSDGDGECDFQEFMAFVAMI |  |  |
| >NCS1_HUMAN/148-176 | >S100G_BOVIN/49-77 |  |  |
| RVDRIFAMMDKNADGKLTLQEFQEGSKAD | TLDELFEELDKNGDGEVSFEEFQVLVKKI |  |  |
| >NCS1_YEAST/100-128 | >S10A1_BOVIN/54-82 |  |  |
| KLSWAFELYDLNHDGYITFDEMLTIVASV | AVDKVMKELDENGDGEVDFQEYVVLVAAL |  |  |
| >NCS1_YEAST/148-176 | >S10AB_PIG/57-85 |  |  |
| RVKKIFKLMDKNEDGYITLDEFREGSKVD | VLDRMMKKLDLDSDGQLDFQEFLNLIGGL |  |  |
| >NCS1_YEAST/64-92 | >TNNC1_CHICK/96-124 |  |  |
| FANHLFTVFDKDNNGFIHFEEFITVLSTT | ELSDLFRMFDKNADGYIDLEELKIMLQAT |  |  |
| >OBL_OBELO/150-171 | >TNNC2_CHICK/22-50 |  |  |
| DCEATFRHCDLDNSGDLDVDEM------- | EFKAAFDMFDADGGGDISTKELGTVMRML |  |  |
| >OBL_OBELO/116-142 | >TNNC2_CHICK/98-126 |  |  |
| --DAVFDIFDKDGSGTITLDEWKAYGKIS | ELANCFRIFDKNADGFIDIEELGEILRAT |  |  |
| >OBL_OBELO/21-49 | >TNNC2_CHICK/58-86 |  |  |
| RHKHMFDFLDINGNGKITLDEIVSKASDD | ELDAIIEEVDEDGSGTIDFEEFLVMMVRQ |  |  |
| >ONCO_RAT/82-109 | >TNNC2_CHICK/134-162 |  |  |
| ETKSLMDAADNDGDGKIGADEFQEMVHS- | DIEDLMKDSDKNNDGRIDFDEFLKMMEGV |  |  |
| >ONCO_RAT/43-71 | >TNNC2_RABIT/95-123 |  |  |
| QVKDIFRFIDNDQSGYLDGDELKYFLQKF | ELAECFRIFDRNADGYIDAEELAEIFRAS |  |  |
| >PDCD6_HUMAN/94-122 | >TNNC2_RABIT/131-159 |  |  |
| DWQNVFRTYDRDNSGMIDKNELKQALSGF | EIESLMKDGDKNNDGRIDFDEFLKMMEGV |  |  |
| >PDCD6_HUMAN/27-55 |  |  |  |
| FLWNVFQRVDKDRSGVISDTELQQALSNG |  |  |  |
| >PDCD6_HUMAN/160-176 |  |  |  |
| RLTDIFRRYDTDQDGWI------------ |  |  |  |
| >PDCD6_HUMAN/130-158 |  |  |  |
| FHDILIRKFDRQGRGQIAFDDFIQGCIVL |  |  |  |
| >PDCD6_MOUSE/94-122 |  |  |  |
| DWQNVFRTYDRDNSGMIDKNELKQALSGF |  |  |  |
| >PDCD6_MOUSE/27-55 |  |  |  |
| FLWNVFQRVDKDRSGVISDNELQQALSNG |  |  |  |
| >PDCD6_MOUSE/160-176 |  |  |  |
| RLTDIFRRYDTDQDGWI------------ |  |  |  |
| >PDCD6_MOUSE/130-158 |  |  |  |
| FHDILIRKFDRQGRGQIAFDDFIQGCIVL |  |  |  |
| >POLC3_CHEAL/47-75 |  |  |  |
| EVRRMMAEIDTDGDGFISFDEFTDFARAN |  |  |  |
| >POLC3_CHEAL/12-40 |  |  |  |
| DRERIFKRFDTNGDGKISSSELGDALKTL |  |  |  |
| >POLC4_BETVE/11-39 |  |  |  |
| ERERIFKRFDANGDGKISAAELGEALKTL |  |  |  |
| >POLC4_BETVE/46-74 |  |  |  |
| EVKHMMAEIDTDGDGFISFQEFTDFGRAN |  |  |  |
| >POLC7_PHLPR/4-32 |  |  |  |
| DMERIFKRFDTNGDGKISLSELTDALRTL |  |  |  |
| >POLC7_PHLPR/39-67 |  |  |  |
| EVQRMMAEIDTDGDGFIDFNEFISFCNAN |  |  |  |
| >POLC7_PHLPR/4-32 |  |  |  |
| DMERIFKRFDTNGDGKISLSELTDALRTL |  |  |  |
| >PRVA_ESOLU/80-108 |  |  |  |
| ETKAFLKAADKDGDGKIGIDEFETLVHEA |  |  |  |
| >PRVA_ESOLU/41-69 |  |  |  |
| DVKKVFKAIDADASGFIEEEELKFVLKSF |  |  |  |
| >PRVA_HUMAN/82-110 |  |  |  |
| ETKMLMAAGDKDGDGKIGVDEFSTLVAES |  |  |  |
| >PRVA_HUMAN/43-71 |  |  |  |
| DVKKVFHMLDKDKSGFIEEDELGFILKGF |  |  |  |
| >PRVA_RAT/43-71 |  |  |  |
| DVKKVFHILDKDKSGFIEEDELGSILKGF |  |  |  |
| >PRVA_RAT/82-110 |  |  |  |
| ETKTLMAAGDKDGDGKIGVEEFSTLVAES |  |  |  |
| >PRVA_TRISE/81-109 |  |  |  |
| ETKALLAAGDSDHDGKIGADEFAKMVAQA |  |  |  |
| >PRVA_TRISE/42-70 |  |  |  |
| QVKEVFEILDKDQSGFIEEEELKGVLKGF |  |  |  |
| >PRVB_CYPCA/81-108 |  |  |  |
| ETKTFLKAGDSDGDGKIGVDEFTALVKA- |  |  |  |
| >PRVB_CYPCA/42-70 |  |  |  |
| DVKKAFAIIDQDKSGFIEEDELKLFLQNF |  |  |  |
| >PRVB_ESOLU/80-107 |  |  |  |
| ETKAFLADGDKDGDGMIGVDEFAAMIKA- |  |  |  |
| >PRVB_ESOLU/41-69 |  |  |  |
| DVKKAFYVIDQDKSGFIEEDELKLFLQNF |  |  |  |
| >Q26068_PLAMG/20-48 |  |  |  |
| EMKEAFTMIDQNRDGFIDINDLKEMFSSL |  |  |  |
| >Q26069_PLAMG/25-53 |  |  |  |
| EMKEAFTMIDQNRDGFIDINDLKEMFSSL |  |  |  |
| >Q39890_SOYBN/121-149 |  |  |  |
| EVEQMIKEADLDGDGQVNYEEFVKMMMTV |  |  |  |
| >Q39890_SOYBN/48-76 |  |  |  |
| ELQDMISEVDADGNGTIEFDEFLSLMAKK |  |  |  |
| >Q39890_SOYBN/12-40 |  |  |  |
| DFKEAFGLFDKDGDGCITVEELATVIRSL |  |  |  |
| >Q39890_SOYBN/85-113 |  |  |  |
| ELKEAFKVFDKDQNGYISASELRHVMINL |  |  |  |
| >Q6LEG8_SOYBN/48-76 |  |  |  |
| ELQDMINEVDADGNGTIDFPEFLNLMARK |  |  |  |
| >Q6LEG8_SOYBN/12-40 |  |  |  |
| EFKEAFSLFDKDGDGCITTKELGTVMRSL |  |  |  |
| >Q6LEG8_SOYBN/85-113 |  |  |  |
| ELKEAFRVFDKDQNGFISAAELRHVMTNL |  |  |  |
| >Q6LEG8_SOYBN/121-149 |  |  |  |
| EVDEMIREADVDGDGQINYEEFVKVMMAK |  |  |  |
| >Q7ZZB9_ONCMY/56-84 |  |  |  |
| ELQEMIDEVDEDGSGTVDFDEFLVMMVRC |  |  |  |
| >Q7ZZB9_ONCMY/132-160 |  |  |  |
| DIEELMKDGDKNNDGKIDYDEFLEFMKGV |  |  |  |
| >Q7ZZB9_ONCMY/96-124 |  |  |  |
| ELADLFCMFDKNADGYIDLQELKVMLEAT |  |  |  |
| >Q868D4_9HEMI/128-156 |  |  |  |
| DLDAMIDEIDADGSGTVDFEEFMGVMTGG |  |  |  |
| >Q868D4_9HEMI/92-120 |  |  |  |
| ELREAFRLYDKEGNGYISTDVMREILAEL |  |  |  |
| >Q8WSQ4_PHYPO/56-84 |  |  |  |
| AFNEMFNEADATGNGKIQFPEFLSMMGRR |  |  |  |
| >Q8WSQ4_PHYPO/20-48 |  |  |  |
| EFKEAFELFDSERTGFITKEGLQTVLKQF |  |  |  |
| >Q8WSQ4_PHYPO/93-121 |  |  |  |
| ILRQAFRTFDPEGTGYIPKAALQDALLNL |  |  |  |
| >Q9XZV2_EUPOC/28-56 |  |  |  |
| EIKEAFDLFDTNKTGSIDYHELKVAMRAL |  |  |  |
| >Q9XZV2_EUPOC/101-129 |  |  |  |
| EILKAFKVFDEDNSGKISLRNLKRVAKEL |  |  |  |
| >Q9XZV2_EUPOC/64-92 |  |  |  |
| EILELMNEYDREGNGYIGFDDFLDIMTEK |  |  |  |
| >Q9XZV2_EUPOC/137-165 |  |  |  |
| ELQAMIDEFDKDQDGEISEQEFLNIMKQT |  |  |  |
| >RECO_BOVIN/101-129 |  |  |  |
| KLEWAFSLYDVDGNGTISKNEVLEIVTAI |  |  |  |
| >RECO_BOVIN/65-93 |  |  |  |
| YAQHVFRSFDANSDGTLDFKEYVIALHMT |  |  |  |
| >RECO_HUMAN/65-93 |  |  |  |
| YAQHVFRSFDSNLDGTLDFKEYVIALHMT |  |  |  |
| >RECO_HUMAN/101-129 |  |  |  |
| KLEWAFSLYDVDGNGTISKNEVLEIVMAI |  |  |  |
| >S100B_BOVIN/53-81 |  |  |  |
| VVDKVMETLDSDGDGECDFQEFMAFVAMI |  |  |  |
| >S100G_BOVIN/49-77 |  |  |  |
| TLDELFEELDKNGDGEVSFEEFQVLVKKI |  |  |  |
| >S100G_PIG/49-77 |  |  |  |
| TLDDLFQELDKNGDGEVSFEEFQVLVKKI |  |  |  |
| >S10A1_BOVIN/54-82 |  |  |  |
| AVDKVMKELDENGDGEVDFQEYVVLVAAL |  |  |  |
| >S10A1_RAT/54-82 |  |  |  |
| AVDKIMKELDENGDGEVDFQEFVVLVAAL |  |  |  |
| >S10AB_PIG/57-85 |  |  |  |
| VLDRMMKKLDLDSDGQLDFQEFLNLIGGL |  |  |  |
| >S10AB_RABIT/56-84 |  |  |  |
| VLDRMMKKLDLNSDGQLDFQEFLNLIGGL |  |  |  |
| >TNNC1_CHICK/56-84 |  |  |  |
| ELQEMIDEVDEDGSGTVDFDEFLVMMVRC |  |  |  |
| >TNNC1_CHICK/132-160 |  |  |  |
| DIEELMKDGDKNNDGRIDYDEFLEFMKGV |  |  |  |
| >TNNC1_CHICK/96-124 |  |  |  |
| ELSDLFRMFDKNADGYIDLEELKIMLQAT |  |  |  |
| >TNNC1_HUMAN/132-160 |  |  |  |
| DIEELMKDGDKNNDGRIDYDEFLEFMKGV |  |  |  |
| >TNNC1_HUMAN/96-124 |  |  |  |
| ELSDLFRMFDKNADGYIDLDELKIMLQAT |  |  |  |
| >TNNC1_HUMAN/56-84 |  |  |  |
| ELQEMIDEVDEDGSGTVDFDEFLVMMVRC |  |  |  |
| >TNNC2_CHICK/22-50 |  |  |  |
| EFKAAFDMFDADGGGDISTKELGTVMRML |  |  |  |
| >TNNC2_CHICK/98-126 |  |  |  |
| ELANCFRIFDKNADGFIDIEELGEILRAT |  |  |  |
| >TNNC2_CHICK/58-86 |  |  |  |
| ELDAIIEEVDEDGSGTIDFEEFLVMMVRQ |  |  |  |
| >TNNC2_CHICK/134-162 |  |  |  |
|  |  |  |  |

**File S1** contains figures S1-S4 and Tables S1-S11 **Supplementary Figure S1** a) Plot of affinity vs. PSSM for the test data set (D5).The calculated correlation coefficient obtained was 0.61 using amino acid frequencies. **Supplementary Figure S2** The isothermal titration calorimetric analysis of Ca^2+^-binding to apo-EhCaBPs.ITC experiments were carried out as described under “Materials and Methods”. Plot of heat absorbed/released (In kcal mol^-1^) per injection of CaCl_2_ as a function of molar ratio of Ca^2+^: protein at 25°C is shown. For all titrations, the top panels represent the raw data (power: time) and the bottom panels represent integrated binding isotherms. The solid line represents the best nonlinear fit to the experimental data. Binding isotherm for A: EhCaBP3; B: EhCaBP4; C: EhCaBP5; D: EhCaBP6 and E: EhCaBP7. Thermodynamic parameters obtained are summarized in Table 1. **Supplementary Figure S3** ROC plots of AC&CC, AC&HC, AC&HC&HYC, AC&HYC&CC and AC&HYC for the datasets D5-D7 set. Receiver operating characteristic (ROC) plot used for depicting relative trade-offs between true positive and false positives. The corresponding AUC value of each model is shown in brackets. **Supplementary Figure S4** Schematic representation of the procedure for model development and feature selection for EF-hand loop region prediction and estimation of binding affinity and its web implementation. The procedure is explained in detail in the “Methods” section. A). A group of sequences with known EF-hand structural motifs were downloaded and further classified into two groups after removing redundant sequences using CD-HIT. The sequences were further converted into binary and amino acid composition (AAC) profiles for SVM input. Models were generated using LIBSVM and were tested on all the datasets (D3-D6) and further validated by scanning the E. *histolytica* proteome. B). Non-redundant sequences of EF-hand loops from known structures were classified into two groups on the basis of scores obtained from position-specific scoring metrics. The sequences were then converted into binary, AAC and different amino acid indices patterns. We have generated both standalone and combinations of features (2, 3, 4, 5) using a Perl script written in-house. The input vectors were trained using LIBSVM and cudized LIBSVM and selected on the basis of their performance on experimental datasets using 5-fold cross validation accuracy threshold > 70 %. The best performing models selected from screening were further validated using three different experimentally derived datasets on EF hand motifs. The final step involved web implementation of the best (AC&HC) model. **Supplementary Table S1** The χ2 value for each amino acid residue is estimated with one degree of freedom and signiﬁcance level P = 0.001. The Σχ2 values are estimated with 19 degrees of freedom and signiﬁcance level P < 0.001. The expected (Exp) and observed (Obs) values and the corresponding χ2 values for amino acid residues and the Σχ2 values for those positions that do not reach 10.8 and 43.8 (for one and 19 degrees of freedom, respectively) are given more significance. **Supplementary Table S2** Test Dataset: Summary of EF hand loops obtained from the literature and their macroscopic binding constant along with CAL-EF-AFi predictions (D5). The classification details with supportive binding constants are listed under “Author’s Note”. (Red-colored affinities are the false negative affinity predictions, and turquoise-colored sequences are the false negative EF loop predictions). **Supplementary Table S3** Independent dataset(D6) summary of EF hand loops obtained from Boguta, et al., 1988 . The table contains average binding constants of Ca^2+^ for troponin C superfamily (TnC) proteins from experimental data reported by various laboratories. The classification details with supportive binding constants are listed under “Author’s Note”. (Red-colored affinities are the false positive predictions). **Supplementary Table S4** Validation dataset summary of EF-hand loops obtained from ITC studies of CaBPs from *E. histolytica* and their macroscopic binding constant according to CAL-EF-AFi’s predictions (D7). The classification details with supportive binding constants are listed under “Author’s Note” (Red-colored affinities are the false positive predictions). **Supplementary Table S5** Predictions of putative EF hand-containing calcium-binding protein and their calcium-binding affinities from the *E. histolytica* proteome. **Supplementary Table S6** The performance and comparison of CAL-EF-AFi with PFAM and Calpred on the E. *histolytica* proteome. Listed are the sequences predicted by CAL-EF-AFi followed by PFAM-based HMM model prediction and CalPred’s predictions. (Legends for CAL-EF-AFi’s prediction: number of Ca^2+^-binding loop sequence prediction, residue number followed by sequence and SVM scores; Legends for PFAM predictions: red-colored region is the loop region predicted, followed by the E-value for the sequence; Legends for CalPred predictions: X: Non-Binding region C: Calcium Binding region). **Supplementary Table S7** Calcium-binding EF-hand protein sequences in FASTA format at 60% sequence redundancy with EF-hand loop region residues labeled in lower case letters **(D1). Supplementary Table S8** The list of 12-mer sequences from non-binding regions of calcium-binding EF-hand proteins greater than 60% sequence redundancy. **Supplementary Table S9** The training data used for estimation of binding affinity were taken from the RCSB based on PSSM scores obtained from the EF-hand loop region. The positive dataset (**D3**) consisted of one hundred forty four 12-mer sequences and there were 124 sequences in the negative dataset (**D4**). **Supplementary Table S10** The redundant set of PDB ids of EF hand-containing calcium-binding proteins. The sequences taken from the RCSB were further processed using CD-HIT and the list if the sequences with different threshold are listed in supplementary Table S11. **Supplementary Table S11** The sequence-wise classification of data obtained from PROSITE and RCSB**-** The data was further processed by using CD-HIT at 90%, 70%, 60%, 50% sequence redundancy cutofffor classification of EF-hand loop Ca^2+^-binding and non-binding region.
